# Beta-hydroxyphosphonate ribonucleoside analogues derived from 4-substituted-1,2,3-triazoles as IMP/GMP mimics: synthesis and biological evaluation

**DOI:** 10.3762/bjoc.12.144

**Published:** 2016-07-18

**Authors:** Tai Nguyen Van, Audrey Hospital, Corinne Lionne, Lars P Jordheim, Charles Dumontet, Christian Périgaud, Laurent Chaloin, Suzanne Peyrottes

**Affiliations:** 1 Institut des Biomolécules Max Mousseron (IBMM), UMR 5247 CNRS – Université de Montpellier - ENSCM, Campus Triolet, cc1705, Place Eugène Bataillon, 34095 Montpellier, France; 2 Centre d'études d'agents Pathogènes et Biotechnologies pour la Santé (CPBS), FRE 3689 CNRS - Université de Montpellier, 1919 route de Mende, 34293 Montpellier, France; 3 Université de Lyon, Université Claude Bernard Lyon 1, INSERM 1052, CNRS 5286, Centre Léon Bérard, Centre de Recherche en Cancérologie de Lyon, 69008 Lyon, France

**Keywords:** cancer, cN-II inhibitors, nucleotide, phosphonate, triazole

## Abstract

A series of seventeen β-hydroxyphosphonate ribonucleoside analogues containing 4-substituted-1,2,3-triazoles was synthesized and fully characterized. Such compounds were designed as potential inhibitors of the cytosolic 5’-nucleotidase II (cN-II), an enzyme involved in the regulation of purine nucleotide pools. NMR and molecular modelling studies showed that a few derivatives adopted similar structural features to IMP or GMP. Five derivatives were identified as modest inhibitors with 53 to 64% of cN-II inhibition at 1 mM.

## Introduction

**Figure 1:**
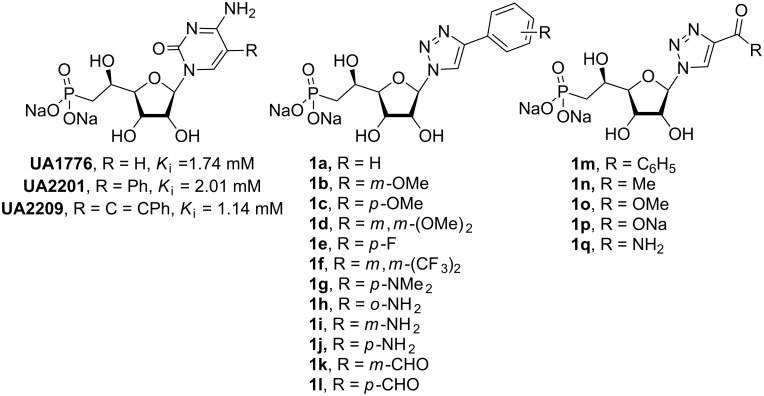
Previous (UA1776, UA2201 and UA2209 [7–8]) and new **1a–q** phosphonate derivatives designed as potential cN-II inhibitors.

Nucleotidases are an important family of enzymes involved in the metabolism of nucleotides [1]. In particular, human cytosolic 5’-nucleotidase II (cN-II) catalyses the dephosphorylation of purine 5’-monophosphate derivatives to their corresponding nucleosides [2–3]. Recent studies on patients affected by haematological malignancies, such as acute myeloid leukaemia (AML) and myelodysplasic syndrome (MS), have demonstrated the involvement of cN-II in the resistance to cytotoxic drugs such as mercaptopurines and suggested the effectiveness of cN-II inhibitors in the treatment of these diseases [4–5]. As a result of our interest in this area, we and others [6] have investigated a number of structure–activity relationships (SAR) [7–8] and various medicinal chemistry approaches [9–10] to identify potential cN-II inhibitors. As part of a research program on phosphonate derivatives of nucleosides as mimics of 5’-monophosphate nucleosides, we explored the SAR of various beta-hydroxyphosphonate analogues. Such derivatives have been extensively studied and exhibited *K*_i_ values in the same range as the known natural substrates (IMP, inosine 5’-monophosphate and GMP, guanosine 5’-monophosphate) [6–7]. In the particular context of cN-II, which is also known as High-*K*_m_ nucleotidase owing to the high substrate concentration required for activity (in the mM range), the *K*_i_ values of approximately 1 mM previously obtained for cytidine-containing derivatives are biologically interesting (Figure 1) [7]. In addition, molecular docking studies have been performed and highlighted the importance of three binding areas within the active site of the protein: a hydrophobic clamp (Phe157, His209 and Tyr210) interacting with the nucleobase, a hydrophilic pocket (Ser251 and Lys215) where the hydroxy groups of the sugar interact and a phosphonate binding site close to the magnesium ion located in the substrate binding site. Thus, as few cytosine-containing analogues were equipotent in terms of cN-II inhibition to their purine counterparts (Figure 1) [7], we were interested in studying the effect of replacing the nucleobase by a 1,2,3-triazole moiety, linked to various substituents, with the aim to retain or modulate essential elements for enzyme recognition. Indeed, the assembly of the triazole ring with various substituents confers to the final compound high flexibility. Initially reported by K. Seley-Radtke’s group, the replacement of the nucleobase by a “flexi-moiety” where the imidazole ring and a six-membered heterocycle is linked through a C–C bond lead to new derivatives behaving as nucleosides and even as enzyme inhibitors [11–13]. The inherent flexibility of the corresponding nucleobase-mimics allows to accommodate steric and electronic clashes encountered in protein binding sites and to interact with other amino acids. Another type of fleximers, obtained by a click chemistry approach, was developed by R. H. E. Hudson to obtain “click-fleximer” as expanded nucleobase mimicking the purine [14]. “Click-fleximer” nucleoside analogues are easily accessible derivatives using copper-catalysed alkyne–azide cycloaddition (CuAAC) and this synthetic methodology allows generating a small library of derivatives depending on the nature of the alkyne employed.

Herein, we report the results of the synthesis and in vitro biological evaluation on the purified human recombinant cN-II of a series of beta-hydroxyphosphonate ribonucleosides including as nucleobases 4-substituted-1,2,3-triazoles (Figure 1).

## Results and Discussion

### Chemistry

**Scheme 1:**
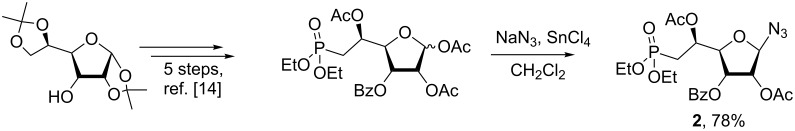
Synthesis of (1-azido-2,5-di-*O*-acetyl-3-*O*-benzoyl-6-deoxy-6-diethylphosphono)-β-ribo-(5*S*)-hexofuranose **2** from commercially available diacetone D-allofuranose [15].

The synthesis of 1,2,3-triazolonucleotides (**1a–o**, Figure 1) was envisaged using the Huisgen 1,3-dipolar cycloaddition of the azido-sugar-phosphonate key-intermediate **2** (Scheme 1) and selected commercially available alkynes. Thus, (1-azido-2,5-di-*O*-acetyl-3-*O*-benzoyl-6-deoxy-6-diethylphosphono)-β-ribo-(5*S*)-hexofuranose (**2**) was obtained in good yield from 1,2,5-tri-*O*-acetyl-3-*O*-benzoyl-6-deoxy-6-diethylphosphono-(α,β)-ribo-(5*S*)-hexofuranose [15] following a glycosylation procedure using sodium azide as nucleophilic entity and tin(IV) chloride as Lewis acid. Under these conditions, the reaction appeared highly stereoselective and only the β-anomer was observed, isolated and characterized on the basis of ^1^H NMR experiments (see section related to NMR studies).

The selection of alkynes was based on their commercial availability as well as the diversity of the chemical group(s) to be introduced. They can be divided in two categories: aromatic alkynes with various substituents (methoxy, amino, formyl…) in *ortho*, *meta* or *para* position, and short alkyne chains such as 3-butyn-2-one or methylpropiolate.

**Scheme 2:**
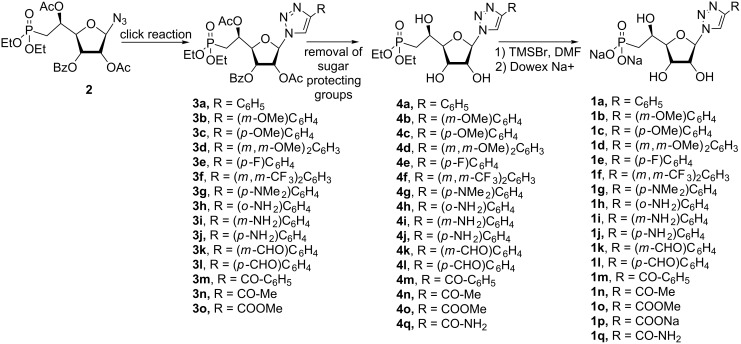
General synthetic pathway for the 1,2,3-triazolo-β-hydroxyphosphonate derivatives.

Starting from intermediate **2**, the CuAAC reaction was either catalysed by CuI or CuSO_4_ (Table 1) and gave rise to the fully-protected nucleotides **3a–o** (Scheme 2) in moderate to good yields. Removal of the sugar protecting groups (acetyl and benzoyl) in basic conditions resulted in the formation of the nucleotides **4a–q** (Scheme 2), which were then treated by trimethylsilyl bromide (TMSBr) to generate the corresponding phosphonic acids. Thus, nucleoside phosphonate analogues **1a–q** (Scheme 2) were isolated as their sodium salts with yields ranging from 21 to 77% over three steps. Structures of all final compounds were unambiguously confirmed on the basis of NMR (^1^H, ^13^C and ^31^P) and MS (MS and HRMS) data analysis (see Supporting Information File 1).

**Table 1:** Summary of the data for the 3 step synthesis of derivatives **1a–q**.

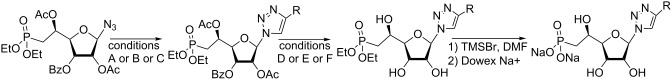			

Compound	R	Conditions^a^	Total yield (%)^b^

**1a**	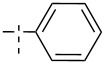	A, D	66
**1b**	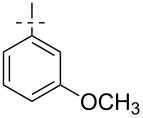	A, D	44
**1c**	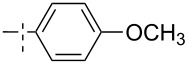	A, D	73
**1d**	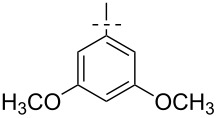	A, D	54
**1e**	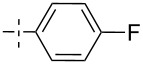	A, D	40^c^
**1f**	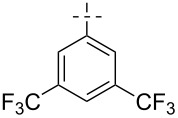	A, D	71
**1g**	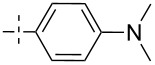	A, D	77
**1h**	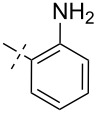	C, D	45^d^
**1i**	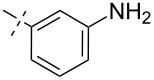	C, D	39^d^
**1j**	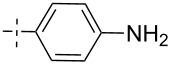	C, D	41^d^
**1k**	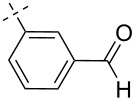	B, E	36^c,e^
**1l**	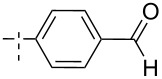	B, E	21^c,e^
**1m**	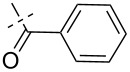	A, D	74
**1n**	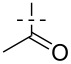	A, D	25
**1o**	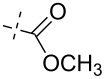	A, F	59
**1p**	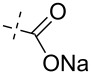	A, F	53^f^
**1q**	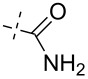	A, F	66

^a^Conditions for click reaction: (A) DIPEA, CuI, THF reflux, *N,N*-diethylenediamine; (B) CuI, CH_3_CN reflux; (C) CuSO_4_, sodium triascorbate, DMF; Conditions for removal of sugar protecting groups: (D) CH_3_OH, NH_3_; (E) Tetramethylguanine, CH_2_Cl_2_/CH_3_OH (1:1); (F) NaOMe/CH_3_OH. ^b^All intermediates and the final compounds were isolated after chromatographic purification; ^c^Conversion rate during the click reaction was not total; ^d^degradation was observed during purification step using RP-chromatography; ^e^due to side-reaction with ammonia, reaction conditions D were replaced by E; ^f^Compound **1p** was obtained by saponification of derivative **1o** in presence of sodium hydroxide.

### NMR studies

**Figure 2:**
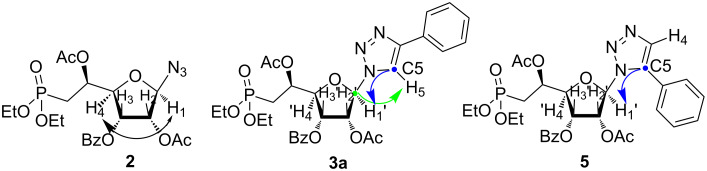
Black arrow indicates ^1^H,^1^H-COSY correlations for compound **2**. Green (C1’ and H5) and blue (H1’ and C5) arrows indicate ^1^H,^13^C-HMBC correlations for compounds **3a** and **5**.

To establish the stereochemistry and/or regiochemistry of the azidation and CuAAC steps, homo- or heteronuclear 2D NMR experiments (see Supporting Information File 2) were performed on compounds **2** and **3a** (Figure 2). In addition, we synthesized compound **5** (resulting from an 1,5-addition) through Ru-catalysed cycloaddition using an adapted procedure from the literature [16].

As the α- and β-anomers of furanoses are diastereomeric, in principle they show distinguishable NMR spectra. In consequence, the proportions of both anomers may be determined by NMR. In our case, the main compound isolated from the glycosylation reaction corresponds to a single anomer as only one signal for the anomeric proton was detectable in the ^1^H NMR spectra. The beta-configuration of the azido-sugar-phosphonate intermediate **2** was established on the basis of the ^1^H,^1^H NMR spectra showing a cross-peak between the anomeric proton H1 and the H4 (Figure 2). Consequently, both protons are located on the same face of the furanose ring and to the opposite face of the C4–C5 bond.

The formation of the triazole ring was confirmed through the presence of a single signal of the triazolyl proton at δ = 7.94 ppm and two signals for the olefin carbon atoms at δ = 148.4 ppm and δ = 119.3 ppm for the C4 and the C5, respectively. The regiochemistry of the cycloaddition was unambiguously established using heteronuclear multiple bond correlation (HMBC) experiments to identify long-range (generally 2- or 3-bonds) coupling between protons and carbons. In compound **3a**, the 1,4-substitution was proven by the fact that the H1‘(δ 6.26 ppm) signal shows a cross-peak with the C-5 (δ 119 ppm), and the C1’ (δ 89.8 ppm) cross-correlates with the signal of the H5 (δ 7.95 ppm). In the case of compound **5** (resulting from an 1,5-addition), the ^1^H NMR spectrum shows significant differences with the one of compound **3a**, especially an aromatic shielding effect on the H1’ signal. The corresponding HMBC 2D-experiment also exhibits a cross-peak between C-5 and H-1’ (Figure 2). Thus, comparative analysis of all NMR data for compounds **3a** and **5** demonstrates that cycloaddition of terminal alkynes catalysed by Cu(I) were highly regioselective and led to 4-substituted-1,2,3-triazoles in the β-hydroxyphosphonate series.

**Figure 3:**
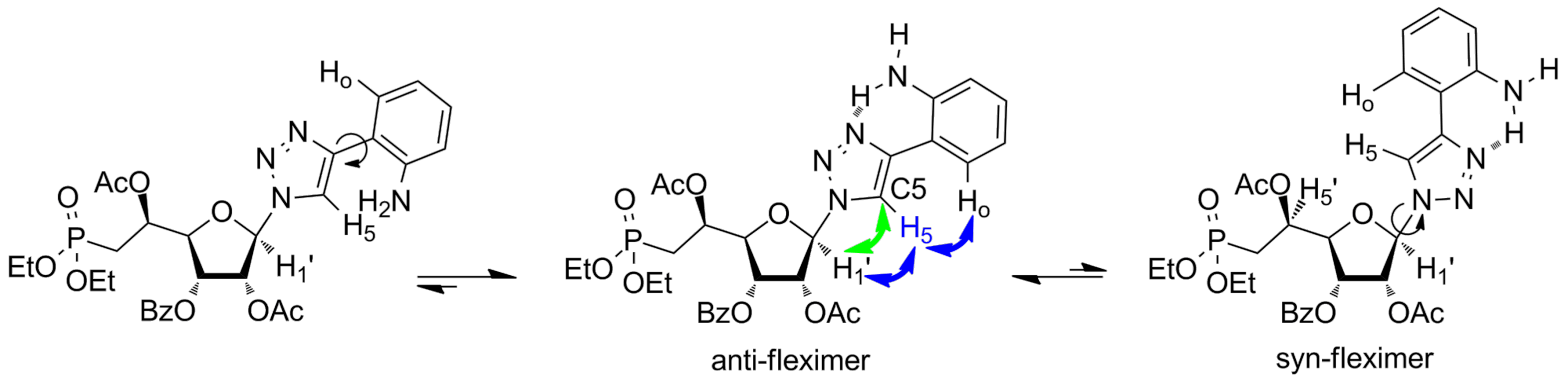
Arrows indicate ^1^H,^1^H-NOESY (blue) and ^1^H,^13^C-HMBC (green) correlations for compound **3h**.

Then, on the basis of the study reported by Hudson et al., we performed extensive 2D-NMR experiments on compounds **3h** and **3i**, respectively. Indeed, these two derivatives may be able to form an H-bond between the exocyclic amino group and the nitrogen atom in position 3 of the triazole ring, in particular for compound **3h**, where the amino group is in the ortho position (Figure 3 and Supporting Information File 2). Thus, the NOESY spectrum showed correlation between the H5 of the triazole ring and the aromatic proton in ortho (Ho), whereas rotation about the C–C bond linking the triazole and the aromatic rings would lead to the disappearance of this correlation peak. In addition, the HMBC spectrum is showing only one correlation peak between the C5 and the anomeric proton (H1’), indicating that rotation around the glycosidic bond may be limited. Therefore, we hypothesized that compound **3h** behaved as a favoured anti-fleximer.

**Figure 4:**
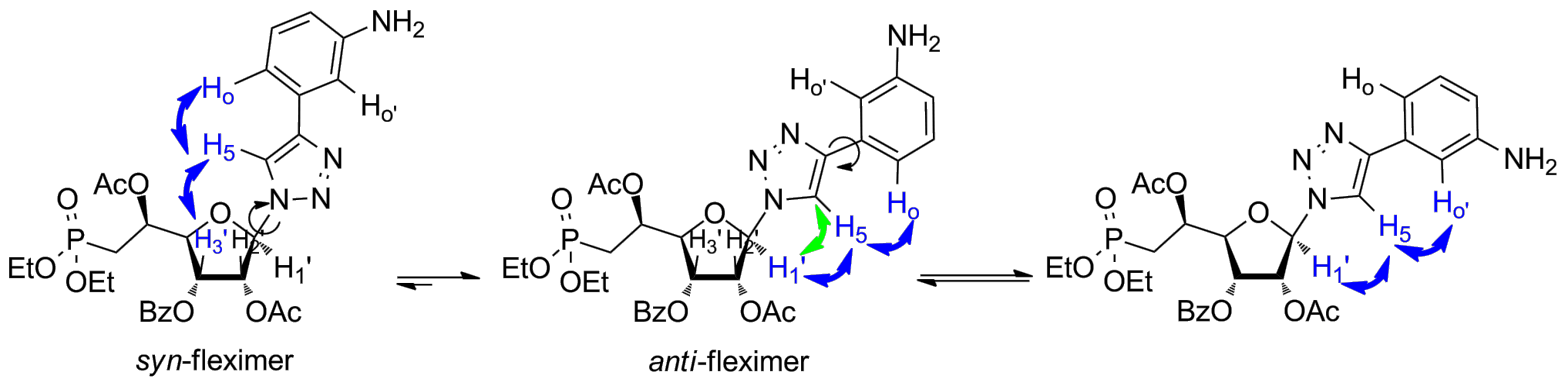
Arrows indicate ^1^H,^1^H-NOESY (blue) and ^1^H,^13^C-HMBC (green) correlations for compound **3i**.

Similar analysis of the NOESY and HMBC spectra of derivative **3i** indicates that this compound behaves as several conformers in solution (Figure 4). Indeed, the NOESY experiment shows correlations between the two protons in the ortho position (H_o_ and H_o’_) of the phenyl ring and the H5 of the triazole. In this case, the position of the exocyclic amino group in meta position does not allow the formation of a strong H-bond and the C–C bond linking the triazole and the aromatic rings is free to rotate. To our surprise, a cross-peak between the H5 of the triazole and the H3’ was observed (as well as with the anomeric proton), indicating that both syn- and anti-conformers may be present.

### Enzyme inhibition and molecular modelling studies

All derivatives were assayed for their ability to inhibit the human 5’-nucleotidase activity in comparison to previously published derivative (UA1776), using a rapid in vitro assay. Briefly, the enzyme catalyzes the hydrolysis of IMP into inosine and inorganic phosphate (IMP + H_2_O ↔ inosine + PO_4_^2−^). This latter product is detected by using a malachite green assay and was quantified by the absorbance according to the phosphate concentration range.

**Figure 5:**
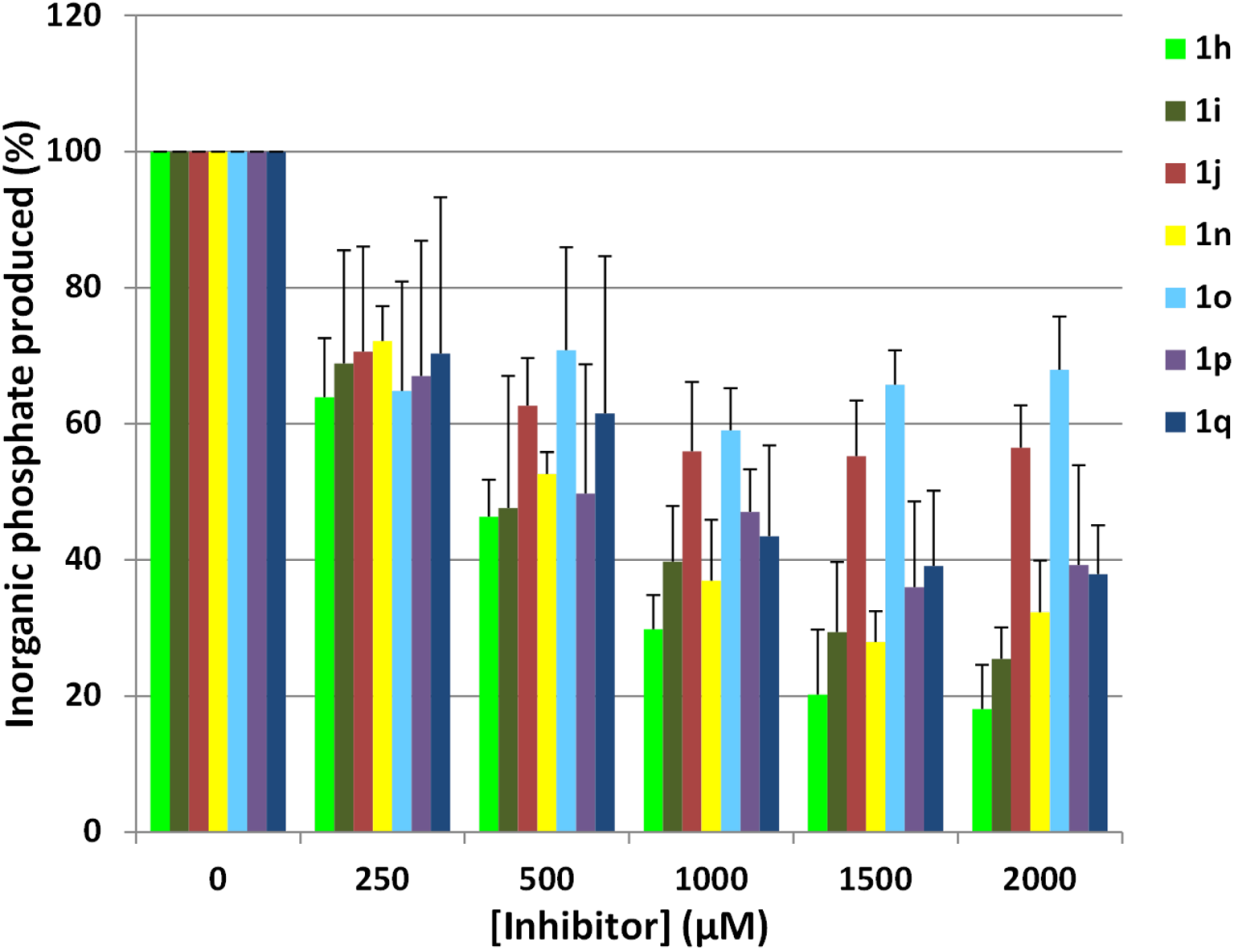
Inhibition of the nucleotidase activity in presence of representative triazole-based derivatives.

Most of the compounds were found to reduce the enzyme activity at high concentrations (>1 mM). However, only a moderate inhibition (Figure 5) was observed for these derivatives indicating that they may not bind very tightly to the enzyme binding site. Nevertheless, five compounds **1i**, **1h**, **1n**, **1o** and **1q** exhibited a more pronounced inhibition at 1 mM (Table 2) with at least 50% of inhibition. The nature, size and orientation of the substituent on the triazole ring seem to play a major role in the inhibitory activity as they should mimic a purine nucleobase, especially hypoxanthine for compounds **1n**, **1o** and **1q**.

**Table 2:** Summary of the in vitro inhibition assays performed on human recombinant cN-II in the presence of the various derivatives and docking scores obtained from molecular modelling calculation.

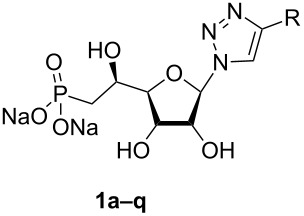					

Compound	R =	% of inhibition at 1 mM	Docking score /NHA^a^	NHA*	Docking score (crude)

**1a**	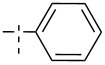	n.d. (precipitated)	6.32	25	158
**1b**	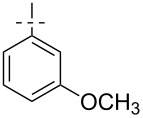	23 ± 19	5.91	27	159.6
**1c**	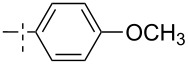	31 ± 3	5.64	27	153.4
**1d**	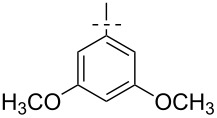	29 ± 13	5.45	29	158.2
**1e**	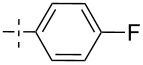	36 ± 1	5.85	26	152.1
**1f**	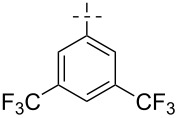	36 ± 14	4.71	33	155.6
**1g**	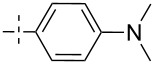	42 ± 0.5	5.73	28	160.5
**1h**	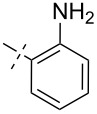	64 ± 7	5.96	26	155
**1i**	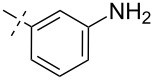	60 ± 8	5.94	26	154.6
**1j**	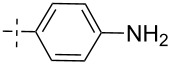	44 ± 10	5.68	26	147.6
**1k**	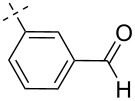	34 ± 1	5.97	27	161.2
**1l**	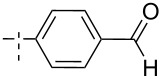	34 ± 0.5	5.85	27	157.9
**1m**	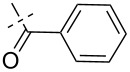	41 ± 0.5	5.78	27	156
**1n**	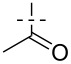	61 ± 9	7.04	22	155
**1o**	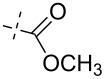	53 ± 6	6.69	23	153.9
**1p**	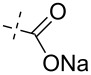	41 ± 6	7.09	22	155.9
**1q**	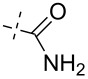	57 ± 13	6.88	22	151.4
**IMP**^b^		–	6.56	23	150.9
**UA1776**	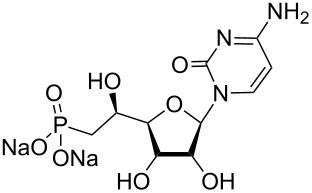	85 ± 5	6.64	22	146.1

^a^Number of heavy atoms (or non-hydrogen atoms); ^b^inosine 5’-monophosphate.

The synthesized compounds may also interact with allosteric binding sites present in cN-II. Indeed, two potential binding sites have been described in the literature [17–18]. According to the X-ray crystallography, kinetics and mutagenesis studies, four effectors have been identified for binding in the effector site 1. The activation of the site 2 is more questionable as solid data is still required to clearly ascertain the biological effect of such activation. However, both sites may be occupied by one common activator (Ap_4_P). One way to evaluate the effect of the synthesized ribonucleoside analogues on the inhibition of allosteric activation will be to include the Ap_4_A effector in the enzymatic assay. Although allosteric inhibition was not expected since these compounds bring only one phosphonate group (compared to more negatively charged activators) such enzymatic assay will be envisaged in future studies.

The most active compounds were further studied in details in order to decipher the mechanism by which they block the enzyme activity. For this purpose, a SAR study was carried out by molecular docking. Attempts to make a direct correlation between the inhibitory activity and the gold-computed docking score (Table 2) were unsuccessful. Indeed, all compounds exhibited a similar score with a value very close to that of IMP or to the previously characterized inhibitor, UA1776. This may be explained by the presence of a phosphonate chain in all compounds which binds to the protein by electrostatic interactions through the magnesium ion and thus largely contributing to the final score. As the substituent varies mainly in size, the docking score was normalized by dividing it by the number of heavy atoms (NHA, non-hydrogen atoms). By this mean and in respect to IMP (used as a control as it is the natural substrate of cN-II) one group of derivatives is predicted as good binder (**1n**, **1o**, **1p** and **1q**) with a normalized score above 6.5 (Table 2). This result is in good agreement with the activity for derivatives **1n**, **1o** and **1q,** even so for compounds **1i** and **1h** predictions were less favourable or over-estimated for derivative **1p**. This observation is not so surprising as docking is dedicated to predict binding affinity and not the activity.

**Figure 6:**
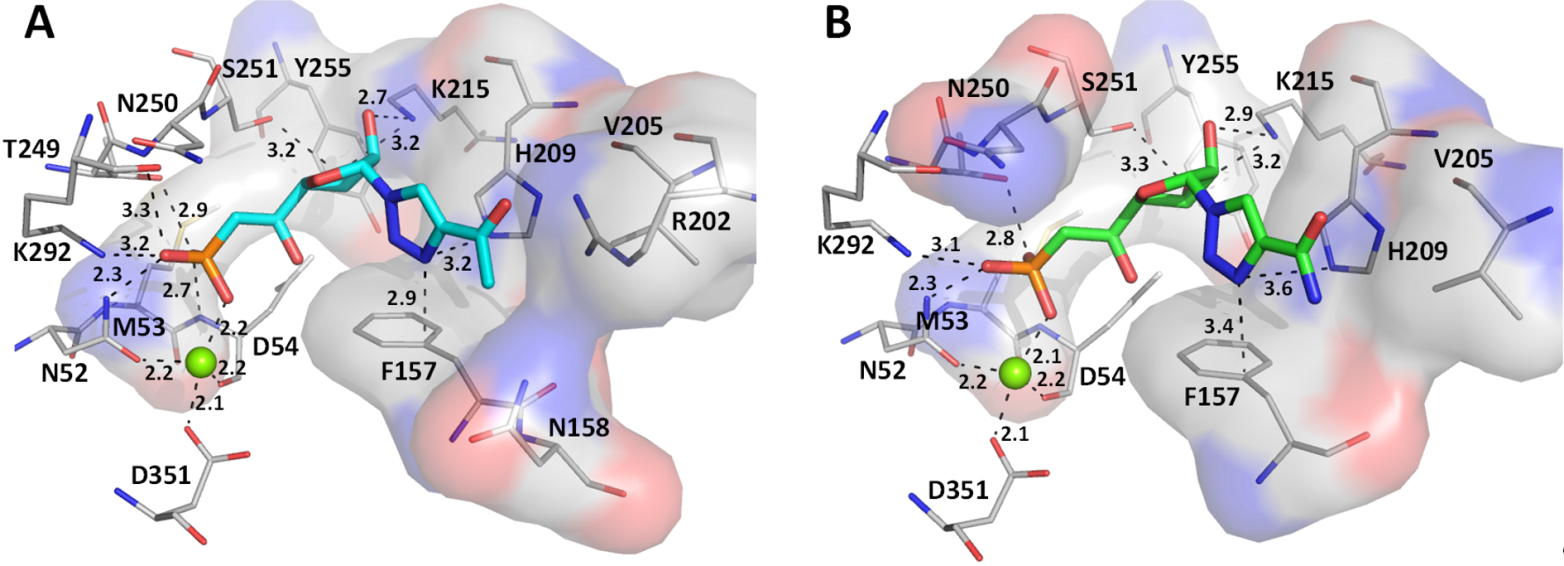
Comparison of the docking poses obtained for two active derivatives in the substrate binding site of cN-II. Main interactions between (A) derivative **1n** or (B) derivative **1q** and cN-II residues.

Nevertheless, we focused on the most active compounds and determined the main interactions with the target protein that will be required for lead optimization. We first analysed compounds with the smallest substituent on the triazole nucleobase (derivatives **1n–q**). As shown in Figure 6, the presence of amide function in position 4 of the triazole ring seems favourable for the inhibitory activity whereas a carboxylic or ester group is less advantageous. As expected, strong ionic interactions between the magnesium ion and the phosphonate oxygen were observed. In addition, our binding predictions for compounds **1n** and **1q** indicated that the ribose moiety is linked to Lys215 by hydrogen bonding between the hydroxy groups and the lysine terminal nitrogen. As the ribose is being stabilized, the triazole ring is correctly positioned between the surrounding hydrophobic residues Phe157 and His209 (at right angle to Phe157). Surprisingly, for these two derivatives the substituents in position 4 of the triazole ring (either an amide or an acetyl group) did not seem to interact with protein residues.

**Figure 7:**
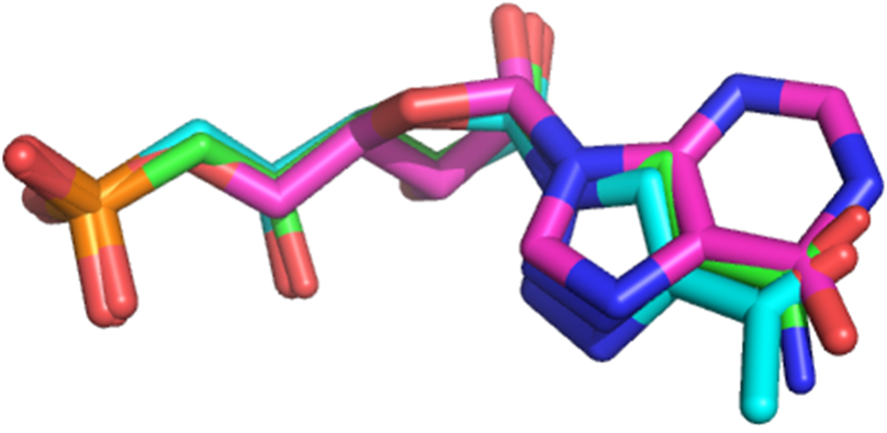
Superimposition of the docking poses obtained for IMP (pink sticks), derivatives **1n** (cyan sticks) and **1q** (green sticks) in the substrate binding site.

As the carbonyl group in position 6 of IMP is interacting with Arg202 in the crystal structure (distance of 3.0 Å in the crystal structure and 3.7 Å with derivative **1n**), we expected that this functionality would constitute a good mimic of the hypoxanthine nucleobase (for IMP) as shown below (Figure 7) with the superimposition of IMP (pink sticks) and derivatives **1n** (cyan sticks) and **1q** (green sticks) in the substrate binding site of cN-II.

**Figure 8:**
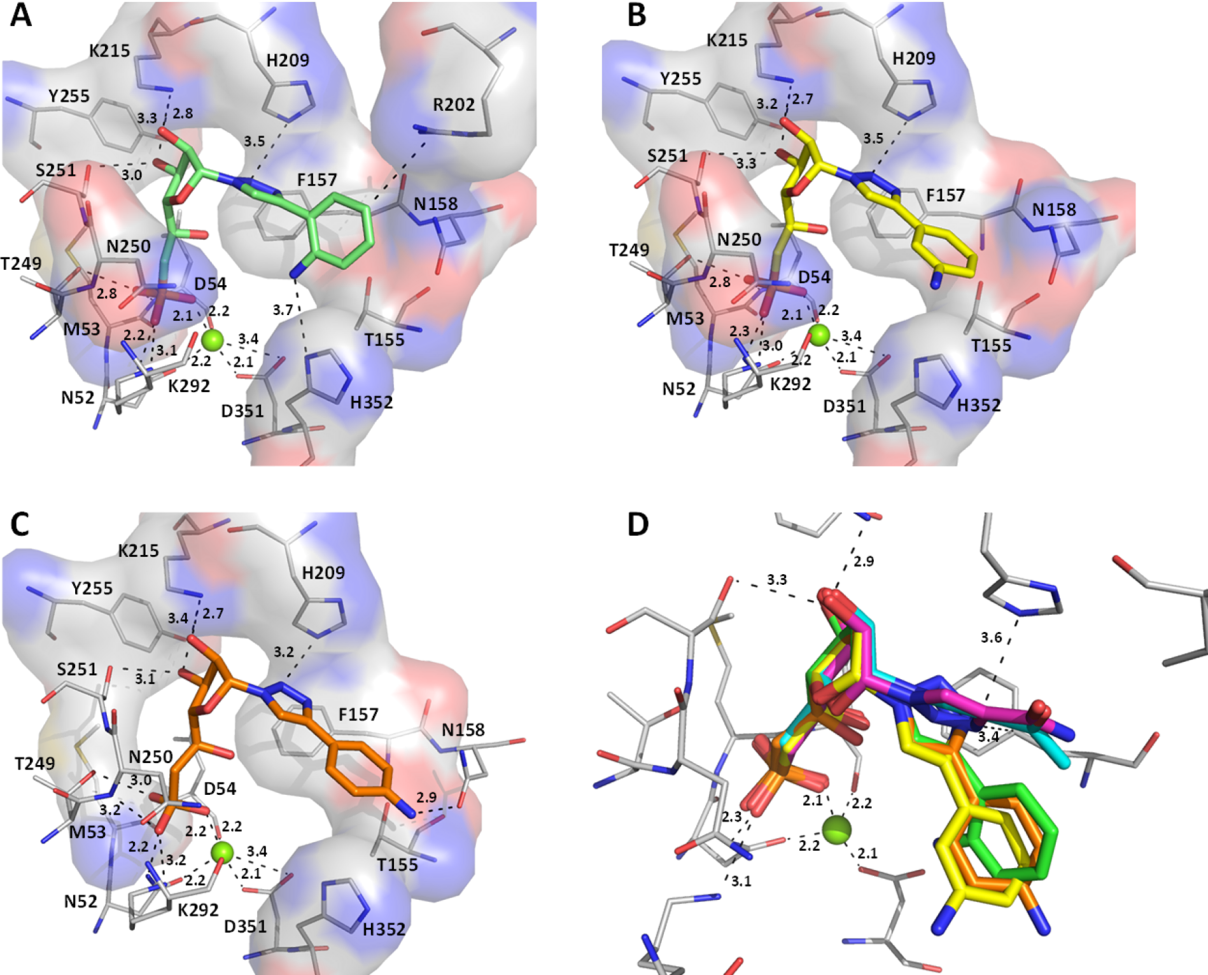
Comparison of the docking poses obtained for three active derivatives in the substrate binding site of cN-II. Main interactions between derivatives (A) **1h** (green stick) or (B) **1i** (yellow sticks) or (C) **1j** (orange sticks) and cN-II residues (depicted in thin stick representation). (D) Superimposition of the docking poses obtained for derivatives **1n** (cyan sticks), **1q** (pink sticks), **1h** (green stick), **1i** (yellow sticks), **1j** (orange sticks) in the substrate binding site.

We then compared the three analogues bearing an aminophenyl substituent on the triazole ring with all possible orientations (*ortho*, *meta* or *para*) for the amino group (derivatives **1h**, **1i** and **1j**). Interestingly, all of them showed very similar binding poses with respect to the positions of the oxygens linked to the phosphorus atom (strong ionic interactions with the magnesium ion), the ribose moiety (formation of hydrogen bonds between the hydroxy groups and Lys215) and the triazole ring oriented towards the hydrophobic residues Phe157 and His209 (Figure 8). However, the position of the phenyl group for derivative **1h** (amino group in the ortho position) is clearly different than the one of derivatives **1i** and **1j** (these last being very similar to each other) and the rotation of the phenyl group appears to be dependent on the orientation of the amino group. According to the inhibition results, derivative **1j** was less potent than expected (in view of the interaction of the *para*-amino phenyl with Asn158) and derivative **1h** was found to be more active. This last may be explained by the interaction of the *ortho*-aminophenyl with His352 residue of cN-II as it represents the only difference with the others (Figure 8A). One should note that in comparison to smallest substituents on the triazole ring (compounds **1n**, **1o** and **1q**) in compounds **1h**, **1i** and **1j** the position of the five-membered ring is rotated by 90° (Figure 8D).

## Conclusion

A small library of seventeen 1’-triazolyl beta-hydroxyphosphonate ribonucleoside analogues was synthesized using convenient Cu(I)-catalysed cycloaddition. These derivatives were evaluated as potential cN-II inhibitors on the purified enzyme. Two derivatives including either an aminophenyl or an amido-substituent in the 4-position of the triazole ring were identified as modest inhibitors. Based on this study and previous SARs on cN-II inhibitors, we believe that optimized derivatives should be able to interact at least with: Phe157 and His209 for the nucleobase, Ser251 and Lys215 for the hydroxy groups of the sugar, and finally with Met53 and Lys292 for the phosphonate group, within the IMP-nucleotide binding site of cN-II.

## Experimental

**General procedure A for click reaction:** The azido-sugar **2** (1 equiv) was dissolved in dry THF (45 mL/mmol) and the required alkyne derivative (5.4 equiv), diisopropylethylamine (1.9 equiv), CuI (0.57 equiv) and DMEDA (5.2 equiv) were added. The reaction mixture was heated to reflux until TLC indicated complete consumption of **2**, then the solvent was removed. The residue was dissolved in EtOAc and washed with H_2_O twice and once with an aqueous solution of EDTA (1%, m/v). The organic layer was dried over MgSO_4_, filtered and the solvent removed. Purification of the crude material on column chromatography on silica gel (CH_2_Cl_2_/EtOAc) afforded the desired product.

**General procedure B for click reaction:** The azido-sugar **2** (1 equiv) was dissolved in acetonitrile (25 mL/mmol) and then the required alkyne derivative (2.5 equiv) and CuI (0.4 equiv) were added. The reaction mixture was heated to reflux until TLC indicated complete consumption of **2**. Then, the reaction mixture was filtered on celite and the solvent was removed. The residue was dissolved in EtOAc and washed with H_2_O twice and once with an aqueous solution of EDTA (1%, m/v). The organic layer was dried over with MgSO_4_, filtered and the solvent was removed. Purification of the crude material on column chromatography on silica gel (CH_2_Cl_2_/EtOAc) afforded the desired product.

**General procedure C for click reaction:** The azido-sugar **2** (1 equiv) was dissolved in DMF (0.8 mL/mmol), the alkyne derivative (5.1 equiv), CuSO_4_ (0.03 equiv) and sodium ascorbate (0.1 equiv) were added to the solution. The reaction mixture was heated at 70 °C until TLC indicated complete consumption of **2**. Then, it was diluted with water and extracted with EtOAc, the organic layers were combined and dried over with MgSO_4_ and concentrated under reduced pressure. The crude material was purified by flash chromatography (CH_2_Cl_2_/EtOAc) to give the desired product.

**General procedure D for removal of sugar protecting groups:** The protected derivative was dissolved in methanolic ammonia (20 mL/mmol) at room temperature and stirred overnight, and then the reaction mixture was concentrated under vacuum. The crude material was purified by flash chromatography (CH_2_Cl_2_/MeOH) to give the desired product.

**General procedure E for diethyl phosphonate removal:** The protected phosphonate (1 equiv) was dissolved in anhydrous DMF (20 mL/mmol) and trimethylsilyl bromide (15 equiv) was added dropwise at 0 °C. The reaction mixture was stirred at room temperature until completion of the reaction was indicated by TLC (isopropanol/water/ammonia, 7:2:1, v/v/v). Then, the reaction was stopped by adding triethylammonium bicarbonate buffer (TEAB 1 M, pH 7) and concentrated to dryness under high vacuum. Column chromatography of the crude materials on reverse phase (gradient: water to methanol 100%) gave the expected phosphonic acid (as triethylammonium salt), which was passed through a Dowex Na^+^ ion exchange column, the desired fractions were collected and freeze dried leading to the title compound as sodium salt.

## Supporting Information

File 1 Description of the materials and methods, and the preparation and characterization of new compounds.

File 2 Copies of spectra for final compounds and NMR studies.
