# Supplementary material for: Beta-hydroxyphosphonate ribonucleoside analogues derived from 4-substituted-1,2,3-triazoles as IMP/GMP mimics: synthesis and biological evaluation
Source: Beilstein J Org Chem. 2016 Jul 18;12:1476–86. doi: 10.3762/bjoc.12.144 (PMC4979751; doi:10.3762/bjoc.12.144)
Supplement: File 1 — Description of the materials and methods, and the preparation and characterization of new compounds. [file Beilstein_J_Org_Chem-12-1476-s001.pdf]

Supporting Information

for

Beta-hydroxyphosphonate ribonucleoside analogues derived from 4-substituted-1,2,3-triazoles as IMP/GMP mimics: synthesis and biological evaluation

Tai Nguyen Van[1], Audrey Hospital[1], Corinne Lionne[2], Lars P. Jordheim[3], Charles Dumontet[3], Christian Périgaud[1], Laurent Chaloin[2] and Suzanne Peyrottes[1,*]

Address: [1]Institut des Biomolécules Max Mousseron (IBMM), UMR 5247 CNRS – Université de Montpellier - ENSCM, Campus Triolet, cc1705, Place Eugène Bataillon, 34095 Montpellier, France [2]Centre d'études d'agents Pathogènes et Biotechnologies pour la Santé (CPBS), FRE 3689 CNRS - Université de Montpellier, 1919 route de Mende, 34293 Montpellier, France and [3]Université de Lyon, Université Claude Bernard Lyon 1, INSERM 1052, CNRS 5286, Centre Léon Bérard, Centre de Recherche en Cancérologie de Lyon, 69008 Lyon, France

Email: Peyrottes Suzanne - peyrottes@univ-montp2.fr

*Corresponding author

Description of the materials and methods, and the preparation and characterization of new compounds

Materials and methods

Nucleotidase activity assays for in vitro evaluation of inhibitory activity of the compounds

The potential inhibition mediated by the final compounds was evaluated either using the Green Malachite Phosphate Assay (Gentaur) as previously described [1] or by HPLC analysis of inosine content. Briefly, in 96-well plate, the purified recombinant human enzyme (cN-II full-length was obtained and purified beforehand according to a previously published procedure [1,2]) was added to a final concentration of 0.1 µM in 80 µl of buffer containing 50 mM imidazole pH 6.5, 500 mM NaCl and 10 mM $MgCl_2$ and the reaction was started by addition of the substrate (100 µM of IMP with an equimolar concentration of Mg^{2+}) and incubated at 37 °C for 3 min and 30 s. Then, the reaction was quenched either by adding Green Malachite reagent or perchloric acid (PCA) 10%. With the green malachite reagent, the inorganic phosphate was quantified by measuring the absorbance at 630 nm using a Tecan plate reader (Sunrise) and compared with a 0–50 µM phosphate calibration curve. When precipitation occurs with this reagent, the reaction was stopped with PCA 10% and the inosine and IMP contents were quantified by HPLC (Waters Alliance) using a Partisphere 5-SAX column (AIT) and 10 mM ammonium phosphate buffer pH 5.5 as mobile phase. All compounds were dissolved in water and assayed in the concentrations range varying from 0 to 2 mM. Results are expressed as the average (± SD) of three independent experiments.

Molecular docking studies

Molecular docking was carried out with the GOLD 5.2 program (Genetic Optimization for Ligand Docking) from CCDC Software Limited [3]. All derivatives were first

modelled using the VegaZZ molecular modelling program [4] and the atomic charges were assigned using the Gasteiger–Marsili empirical atomic partial charges [5]. The potential energy of all compounds was minimized using 500 steps of steepest descent followed by 5,000 steps of conjugate gradient (tolerance of 0.01 kcal/mol. Å). Docking was performed on the cN-II crystal structure solved in presence of IMP (PDB 2XCW) by applying 50 genetic algorithm runs for conformational poses searching with a radius of 12 Å around the target atom, Mg^{2+} ion (located in the substrate binding site). Structural water molecules present in the crystal structure and near the IMP binding site were retained and allowed to contribute with a 2 Å cut-off of translational and rotational freedom. Docking poses were analysed by the clustering method (complete linkage) from the rmsd matrix of ranking solutions. Solutions were classified according to their respective scores calculated by the Gold score scoring function. Structural analysis and visualization of docking poses was achieved with the PyMOL Molecular Graphics System (version 1.3, Schrödinger, LLC).

Description of general methods for chemical synthesis of the ß-hydroxy-phosphonate analogues may be gleaned from the literature [2, 6].

1,2,5-Tri-*O*-acetyl-3-*O*-benzoyl-6-deoxy-6-diethylphosphono-(α,β)-ribo-(5S)-hexo-furanose [6] used as starting material was obtained according to previously published procedure.

(1-Azido-2,5-di-*O*-acetyl-3-*O*-benzoyl-6-deoxy-6-diethylphosphono)-β-ribo-(5S)-hexofuranose (2)

To a solution of 1,2,5-tri-*O*-acetyl-3-*O*-benzoyl-6-deoxy-6-diethylphosphono-(α,β)-ribo-(5*S*)-hexofuranose (5.02 g, 9.46 mmol) in dry dichloromethane (35 mL) was

added sodium azide (1.35 g, 2.2 equiv) and $SnCl_4$ (0.9 mL, 9 equiv). The reaction was stirred at room temperature until completion was observed by TLC (DCM/EtOAc, 4/6, v/v) and quenched by addition of saturated aqueous $NaHCO_3$. The resulting precipitate was filtered on celite and washed with EtOAc. The mixture was extracted 3 times with EtOAc, the organics layers were dried over $MgSO_4$, filtered and evaporated under reduce pressure. A column chromatography of the crude material on silica gel (DCM/EtOAc, **4**:**6**, v/v) afford the isomer β **2** (3.78 g, 78%) as a colourless oil.

Rf (CH_2Cl_2/EtOAc, 4/6, v/v) 0.56. 1H NMR (400 MHz, $CDCl_3$) δ = 8.04-7.98 (m, 2H, H-Ar), 7.60-7.54 (m, 1H, H-Ar), 7.52-7.43 (m, 2H, H-Ar), 5.65 (t, J = 5.4Hz, 1H, H-3), 5.50-5.42 (m, 1H, H-5), 5.43 (d, 1H, J = 2.6 Hz, H-1), 5.25-5.21 (m, 1H, H-2), 4.61-4.58 (m, 1H, H-4), 4.15-3.90 (m, 4H, O-CH_2-CH_3), 2.28-2.10 (m, 2H, H-6, H-6'), 2.07, 2.02 (2s, 6H, Ac), 1.30 (dt, J = 20.8 and 7.1 Hz, 6H, O-CH_2-$C\underline{H}_3$). 13C NMR (100 MHz, $CDCl_3$) δ = 169.7, 169.3 (C=O, Ac), 164.9 (C=O, Bz), 133.7, 129.7, 128.8, 128.6 (C-Ar), 93.1 (C-1), 82.7 (d, J = 10.3 Hz, C-4), 74.8 (C-2), 70.8 (C-3), 67.9 (d, J = 1.2 Hz, C-5), 62.1, 62.0 (2d, J = 6.5, O-$\underline{C}H_2$-CH_3), 28.1 (d, J = 142.3 Hz, C-6), 20.9, 20.4 (CH_3, Ac), 16.3 (d, J = 5.6 Hz, O-CH_2-$\underline{C}H_3$). 31P NMR (121 MHz, $CDCl_3$) δ = 25.3. MS ESI-QTof>0, m/z 514.16 (M+H) +, 1027.31 (2M+H) +. HR-MS Calculated for $C_{21}H_{29}N_3O_{10}P$: 514.1591; found: 514.1591.

3-*O*-Benzoyl-6-deoxy-2,5-*O*-diacetyl-6-diethylphosphono-1-(4-phenyl-1*H*-1,2,3-triazol-1-yl)-β-D-allofuranose (3a)

Treatment of compound **2** (233 mg, 0.45 mmol) following general procedure A gave rise to the expected derivative 3a as a pale yellow oil (232 mg, 83%).

Rf (CH_2Cl_2/EtOAc, 1/1, v/v) 0.40. 1H NMR (400 MHz, $CDCl_3$) δ = 8.12-8.05 (m, 2H, H-Ar), 7.94 (s, 1H, H-triazole), 7.90-7.82 (m, 2H, H-Ar), 7.65-7.58 (m, 1H, H-Ar), 7.54-7.36 (m, 5H, H-Ar), 6.27 (d, J = 4.5 Hz, 1H, H-1'), 6.14-6.02 (m, 2H, H-2', H-3'), 5.59-5.51 (m, 1H, H-5'), 4.77 (t, J = 3.8 Hz, 1H, H-4'), 4.16-4.08 (m, 4H, O-CH_2-CH_3), 2.40-2.13 (m, 2H, H-6', H-6''), 2.05, 2.04 (2s, 6H, Ac), 1.31-1.25 (m, 6H, O-CH_2-$C\underline{H}_3$). 13C NMR (100 MHz, $CDCl_3$) δ = 169.8, 169.2 (C=O, Ac), 164.9 (C=O, Bz), 148.2 (C=C) 133.8, 129.9, 129.8, 128.9, 128.8, 128.7, 128.5, 125.9 (C-Ar), 119.2 (C=CH), 89.7 (C-1'), 84.5 (d, J = 9.7 Hz, C-4'), 74.3 (C-2'), 70.6 (C-3'), 67.6 (C-5'), 62.2, 62.1 (2d, J = 7.01 Hz, O-$\underline{C}H_2$-CH_3), 28.3 (d, J = 141.7 Hz, C-6'), 20.9, 20.3 (CH_3, Ac), 16.4 (d, J = 6.06 Hz, O-CH_2-$\underline{C}H_3$). 31P NMR (121 MHz, $CDCl_3$) δ = 25.1. MS ESI-QTof >0, m/z 616.2 (M+H) +. HR-MS Calculated for $C_{29}H_{35}N_3O_{10}P$: 616.2060; found: 616.2065. UV (EtOH 95) λmax = 237 nm (εmax = 36000).

3-*O*-Benzoyl-6-deoxy-2,5-*O*-diacetyl-6-diethylphosphono-1-(5-phenyl-1H-1,2,3-triazol-1-yl)-β-D-allofuranose (5)

The azido sugar **2** (204 mg, 0.40 mmol) was dissolved in DMF (10 mL/mmol), then phenyl acetylene (176 µL, 1.6 mmol) and Cp*RuCl$(PPh_3)_2$ (16 mg, 0.02 mmol) were added and the reaction mixture was heated at 75°C overnight. The development of reaction was followed by TLC, after complete consumption of **2**, the solvent was removed. The residue was dissolved in EtOAc and washed with sodium chloride saturated solution. The organic layer was dried over with $MgSO_4$, filtered and the solvent was removed. Purification of the crude material on column chromatography on silica gel (DCM/EtOAc) afforded the expected derivative **5** as a pale yellow oil (108 mg, 68%).

Rf (CH_2Cl_2/EtOAc: 1/1) 0.25. 1H NMR (400 MHz, $CDCl_3$) δ 8.03 (dd, J = 8.2, 1.1 Hz, 2H, H-Ar), 7.73 (s, 1H, H-triazole), 7.69 – 7.62 (m, 1H, H-Ar), 7.61 – 7.56 (m, 1H, H-Ar), 7.54 – 7.50 (m, 4H, H-Ar), 7.48 – 7.42 (m, 2H, H-Ar), 6.36 (dd, J = 5.3, 3.8 Hz, 1H, H-2'), 6.21 (t, J = 5.4 Hz, 1H, H-3'), 5.93 (d, J = 3.8 Hz, 1H, H-1'), 5.53 (dtd, J = 11.6, 6.7, 3.4 Hz, 1H, H-5'), 4.78 (dd, J = 5.3, 3.4 Hz, 1H, H-4'), 4.13 – 4.01 (m, 4H, O-C$\underline{H}_2$-CH_3), 2.22 (dddd, J = 45.7, 19.1, 15.5, 6.8 Hz, 2H, H-6', H-6''), 2.03, 1.96 (2s, 6H, Ac), 1.28 (dt, J = 10.2, 7.1 Hz, 6H, O-CH_2-C$\underline{H}_3$). 13C NMR (100 MHz, $CDCl_3$) δ 169.9, 169.3 (C=O, Ac), 165.0 (C=O, Bz), 139.5 (C=CH), 133.8 (C-Ar), 133.1 (C=CH), 132.1, 130.0, 129.9, 129.4, 129.2, 129.0, 128.7, 87.4 (C-1'), 84.2 (d, J = 9.7 Hz, C-4'), 74.5 (C-2'), 70.9 (C-3'), 67.3 (C-5'), 62.2, 62.0 (2d, J = 5.8 Hz, O-$\underline{C}H_2$-CH_3), 27.2 (d, J = 141.4 Hz, C-6'), 21.0, 20.5 (CH_3, Ac), 16.4 (d, J = 5.9 Hz, O-CH_2-$\underline{C}H_3$). 31P NMR (121 MHz, $CDCl_3$) δ 25.31. MS ESI-QTof >0, m/z 616.21 (M+H) +, 617.21 (M+2H) +, 643.17 (M+Na+5H) +, 661.26 (M+2Na) +, 745.36 (M+6Na+8H) +, 1231.41 (2M+H) +. HR-MS Calculated for $C_{29}H_{35}N_3O_{10}P$: 616.2060; found: 616.2064.

6-Deoxy-6-diethylphosphono-1-(4-phenyl-1*H*-1,2,3-triazol-1-yl)-β-D-allofuranose (4a)

Treatment of compound **3a** (189 mg, 0.31 mmol) following general procedure D gave rise to the expected derivative **4a** (115 mg, 88%).

Rf (CH_2Cl_2/EtOAc, 1/1, v/v) 0.40. 1H NMR (400 MHz, DMSO) δ = 8.76 (s, 1H, C-H triazole), 7.88 (d, J = 7.31 Hz, 2H, H-Ar), 7.48 (t, J = 7.74 Hz, 2H, H-Ar), 7.35 (t, J = 7.33 Hz, 1H, H-Ar), 5.96 (d, J = 5.65 Hz, 1H, H-1'), 5.60 (d, J = 6.45 Hz, 1H, OH), 5.52 (d, J = 5.91 Hz, 1H, OH), 5.26 (d, J = 5.11 Hz, 1H, OH), 4.46 (q, J = 5.65 Hz, 1H, H-2'), 4.26 (q, J = 3.24 Hz, 1H, H-3'), 4.01-3.97 (m, 6H, H-4', H-5', O-C$\underline{H}_2$-CH_3), 2.25-1.86 (m, 2H, H-6', H-6''), 1.21 (td, J = 4.15 and 7.02Hz, 6H, O-CH_2-C$\underline{H}_3$). 13C NMR (100 MHz, DMSO) δ = 146.4 (C=CH), 130.2, 128.6, 127.7, 124.9 (C-Ar), 119.5

(C=CH), 91.6 (C-1'), 88.2 (d, J = 15.6 Hz, C-4'), 74.9 (C-2'), 69.2 (C-3'), 65.4 (d, J = 4.19 Hz, C-5'), 61.0, 60.7 (2d, J = 6.16 Hz, O-$\underline{C}H_2$-CH_3), 29.8 (d, J = 138.7 Hz, C-6'), 15.9 (d, J = 5.98 Hz, O-CH_2-$\underline{C}H_3$). 31P NMR (121 MHz, $CDCl_3$) δ = 29.4. MS ESI-QTof >0, m/z 428.16 (M+H) +, 855.31 (2M+H) +. HR-MS Calculated for $C_{18}H_{27}N_3O_7P$: 428.1587; found: 428.1585.

6-Deoxy-6-phosphono-1-(4-phenyl-1*H*-1,2,3-triazol-1-yl)-β-D-allofuranose disodium salt (1a)

Treatment of compound **4a** (105 mg, 0.25 mmol) following general procedure G gave rise to the expected derivative **1a** (92 mg, 90%).

Rf (iPrOH/NH_4OH/H_2O: 7/2/1) 0.17. 1H NMR (400 MHz, D_2O) δ = 8.42 (s, 1H, C-H triazole), 7.82-7.70 (m, 2H, H-Ar), 7.58-7.41 (m, 3H, H-Ar), 6.08 (d, J = 5.06 Hz, 1H, H-1'), 4.74-4.68 (m, 1H, H-2'), 4.59-4.55 (m, 1H, H-3'), 4.28-4.12 (m, 2H, H-4', H-5'), 1.95-1.80 (m, 2H, H-6', H-6''). 13C NMR (100 MHz, D_2O) δ = 147.9 (C=C), 129.3, 129.3, 129.0, 125.8 (C-Ar), 120.7 (CH triazole), 91.7 (C-1'), 88.4 (d, J = 13.9 Hz, C-4'), 75.1 (C-2'), 69.4 (C-3'), 67.3 (d, J = 3.1 Hz, C-5'), 32.1 (d, J = 130.5 Hz, C-6'). 31P NMR (121 MHz, $CDCl_3$) δ = 20.3. MS ESI-QTof >0, m/z 394.08 (M-Na+2H) +, 765.17 (2M-3Na+4H) +. MS ESI-QTof <0, m/z 370.08 (M-2Na+H)-. HR-MS Calculated for $C_{14}H_{18}N_3O_7PNa$: 394.0780; found: 394.0780. UV (H_2O) λmax = 240 nm (εmax = 15000).

3-*O*-Benzoyl-6-deoxy-2,5-*O*-diacetyl-6-diethylphosphono-1-(4-(3-methoxy-phenyl)-1*H*-1,2,3-triazol-1-yl)-β-D-allofuranose (3b)

Treatment of compound **2** (351 mg, 0.68 mmol) following general procedure A gave rise to the expected derivative **3b** as a white oil (333 mg, 76%).

Rf (CH_2Cl_2/AcOEt: 1/1) 0.34. 1H NMR (300 MHz, $CDCl_3$) δ 8.12 – 8.06 (m, 2H, H-Ar), 7.93 (s, 1H, H-triazole), 7.67 – 7.59 (m, 1H, H-Ar), 7.53 – 7.44 (m, 3H, H-Ar), 6.91

(dt, J = 7.0, 2.4 Hz, 1H, H-Ar), 6.25 (d, J = 4.7 Hz, 1H, H-1'), 6.09 – 5.94 (m, 2H, H-2', H-3'), 5.52 (ddt, J = 11.8, 10.5, 5.3 Hz, 1H, H-5'), 4.78 (t, J = 3.9 Hz, 1H, H-4'), 4.18 – 4.00 (m, 4H, O-C$\underline{H}_2$-CH_3), 3.88 (s, 3H, OCH_3), 2.25 (dqd, J = 19.3, 15.5, 6.7 Hz, 2H, H-6', H-6''), 2.04 (d, J = 4.5 Hz, 6H, H-Ac), 1.28 (td, J = 7.1, 2.8 Hz, 6H, O-CH_2-C$\underline{H}_3$). 13C NMR (75 MHz, $CDCl_3$) δ 169.9, 169.4 (C=O, Ac), 165.1 (C=O, Bz), 160.2 (C-Ar), 148.3 (C=C), 133.9, 131.4, 130.1, 129.9, 129.0, 128.7, 119.5 (C=CH), 118.4, 114.8, 111.0 (C-Ar), 89.8 (C-1'), 84.5 (d, J = 9.5 Hz, C-4'), 74.4 (C-3'), 70.8 (C-2'), 67.7 (C-5'), 62.2,62.0 (2d, J = 7.0 Hz, O-$\underline{C}H_2$-CH_3), 55.5 (O-CH_3), 27.5 (d, J = 141.5 Hz, C-6'), 21.1, 20.5 (CH_3, Ac), 16.5 (d, J = 6.1 Hz, O-CH_2-$\underline{C}H_3$). 31P NMR (81 MHz, $CDCl_3$) δ 25.14. MS ESI-QTof>0, m/z 646.22 $(M+H)^+$, 647.22 $(M+2H)^+$, 668.19 $(M+Na)^+$. HR-MS Calculated for $C_{30}H_{37}N_3O_{11}P$: 646.2166 (M+H) +; found: 646.2170.

6-Deoxy-6-diethylphosphono-1-(4-(3-methoxyphenyl)-1*H*-1,2,3-triazol-1-yl)-β-D-allofuranose (4b)

Treatment of compound **3b** (313 mg, 0.49 mmol) following general procedure D gave rise to the expected derivative **4b** as white solid (219 mg, 99%).

Rf (CH_2Cl_2/MeOH: 9/1) 0.39. 1H NMR (300 MHz, DMSO) δ 8.77 (s, 1H, H-triazole), 7.51 – 7.31 (m, 3H, H-Ar), 6.92 (dd, J = 8.1, 1.4 Hz, 1H, H-Ar), 5.94 (d, J = 5.6 Hz, 1H, H-1'), 5.60 (d, J = 6.5 Hz, 1H, OH), 5.52 (d, J = 5.8 Hz, 1H, OH), 5.27 (d, J = 5.1 Hz, 1H, OH), 4.44 (dd, J = 11.3, 5.7 Hz, 1H, H-2'), 4.25 (dd, J = 7.9, 4.9 Hz, 1H, H-3'), 4.09 – 3.87 (m, 6H, H-4', H-5', O-C$\underline{H}_2$-CH_3), 3.82 (s, 3H, OCH_3), 2.11 – 1.79 (m, 2H, H-6', H-6''), 1.21 (td, J = 7.0, 3.5 Hz, 6H, O-CH_2-C$\underline{H}_3$). 13C NMR (75 MHz, DMSO) δ 159.3 (C-Ar), 146.2 (C=C), 131.5, 129.7 (C-Ar), 119.7 (C=CH), 117.2, 113.3, 110.2 (C-Ar), 91.5 (C-1'), 88.1 (d, J = 15.7 Hz, C-4'), 74.8 (C-2'), 69.1 (C-3'), 65.3 (d, J = 4.1 Hz, C-5'), 60.7, 60.5 (2d, J = 6.1 Hz, O-$\underline{C}H_2$-CH_3), 54.8 (O-CH_3), 29.0 (d, J = 138.7 Hz, C-6'), 15.8 (d, J = 6.0 Hz, O-CH_2-$\underline{C}H_3$). 31P NMR (81 MHz, DMSO)

δ 29.60. MS ESI-QTof>0, m/z 458.17 (M+H) +, 459.17 (M+2H) +, 480.15 (M+Na) +, 915.33(2M+H) +. HR-MS Calculated for $C_{19}H_{29}N_3O_8P$: 458.1692 (M+H) +; found: 458.1691.

6-Deoxy-6-phosphono-1-(4-(3-methoxypheny)-1*H*-1,2,3-triazol-1-yl)-β-D-allofuranose (disodium salt) (1b)

Treatment of compound **4b** (181 mg, 0.40 mmol) following general procedure E gave rise to the expected derivative **1b** as a white solid (105 mg, 59%).

Rf (iPrOH/NH_4OH/H_2O: 7/2/1) 0.13. 1H NMR (400 MHz, D_2O) δ 8.33 (s, 1H, H-triazole), 7.29 (t, J = 7.9 Hz, 1H, H-Ar), 7.22 (d, J = 7.7 Hz, 1H, H-Ar), 7.10 (s, 1H, H-Ar), 6.85 (d, J = 8.2 Hz, 1H, H-Ar), 6.07 (d, J = 4.9 Hz, 1H, H-1'), 4.66 (t, J = 5.1 Hz, 1H, H-2'), 4.55 (t, J = 4.7 Hz, 1H, H-3'), 4.29 – 4.17 (m, 2H, H-4', H-5'), 3.76 (s, 3H), 1.97 – 1.74 (m, 2H, H-6', H-6''). 13C NMR (100 MHz, D_2O) δ 160.9 (C-Ar), 149.2 (C=C), 132.3, 132.2, 122.5 (C-Ar), 120.2 (C=CH), 116.2, 112.5 (C-Ar), 93.5 (C-1'), 90.1 (d, J = 13.8 Hz, C-4'), 77.0 (C-2'), 71.1 (C-3'), 69.2 (d, J = 2.8 Hz, C-5'), 57.2 (OCH_3), 33.3 (d, J = 130.0 Hz, C-6'). 31P NMR (121 MHz, D_2O) δ 20.05. MS ESI-QTof>0, m/z 402.11 (M-2Na+3H) +, 424.09 (M-Na+2H) +, 891.13 (2M+H) +. MS ESI-QTof <0, m/z 400.09 (M-2Na+H)-, 801.19 (2M-4Na+3H)-. HR-MS Calculated for $C_{15}H_{19}N_3O_8Na_2P$: 446.0705 (M+H) +; found: 446.0704. UV (H_2O) λ1max = 211 nm (ε1max = 35653); λ2max = 243 nm (ε2max = 16146); λ3max = 286 nm (ε3max = 3954).

3-*O*-Benzoyl-6-deoxy-2,5-*O*-diacetyl-6-diethylphosphono-1-(4-(4-methoxy-phenyl)-1*H*-1,2,3-triazol-1-yl)-β-D-allofuranose (3c)

Treatment of compound **2** (346 mg, 0.67 mmol) following general procedure A gave rise to the expected derivative **3c** as a white oil (357 mg, 82%).

Rf (CH_2Cl_2/AcOEt: 1/1) 0.27. 1H NMR (300 MHz, $CDCl_3$) δ 8.10 (dd, J = 8.4, 1.3 Hz, 2H, H-Ar), 7.86 (s, 1H, H-triazole), 7.83 – 7.78 (m, 1H, H-Ar), 7.78 – 7.74 (m, 1H, H-Ar), 7.70 – 7.59 (m, 1H, H-Ar), 7.55 – 7.46 (m, 2H, H-Ar), 7.05 – 6.92 (m, 2H, H-Ar), 6.26 (d, J = 4.7 Hz, 1H, H-1’), 6.04 (dt, J = 9.5, 5.4 Hz, 2H, H-2’, H-3’), 5.53 (dtd, J = 10.5, 6.7, 3.8 Hz, 1H, H-5’), 4.78 (t, J = 3.9 Hz, 1H, H-4’), 4.23 – 4.02 (m, 4H, O-C$\underline{H}_2$-CH_3), 3.87 (s, 3H, OCH_3), 2.44 – 2.09 (m, 2H, H-6’, H-6’’), 2.06 (d, J = 4.3 Hz, 6H, H-Ac), 1.30 (td, J = 7.1, 2.5 Hz, 6H, O-CH_2-C$\underline{H}_3$). 13C NMR (75 MHz, $CDCl_3$) δ 170.1, 169.5 (C=O, Ac), 165.1 (C=O, Bz), 160.2 (C-Ar), 148.3 (C=C), 134.1, 130.0, 129.1, 128.8, 127.2, 123.1 (C-Ar), 118.4 (C=CH), 114.6 (C-Ar), 89.8 (C-1’), 84.6 (d, J = 9.5 Hz, C-4’), 74.5 (C-3’), 70.9 (C-2’), 67.9 (C-5’), 62.5, 62.2 (2d, J = 6.8 Hz, O-$\underline{C}H_2$-CH_3), 55.6 (O-CH_3), 27.6 (d, J = 141.5 Hz, C-6’), 21.2, 20.6 (CH_3, Ac), 16.6 (d, J = 6.1 Hz, O-CH_2-$\underline{C}H_3$). 31P NMR (81 MHz, $CDCl_3$) δ 25.43. MS ESI-QTof>0, m/z 646.22 (M+H) +, 647.22 (M+2H) +, 668.20 (M+Na) +. HR-MS Calculated for $C_{30}H_{37}N_3O_{11}P$: 646.2166 (M+H) +; found: 646.2176.

6-Deoxy-6-diethylphosphono-1-(4-(4-methoxyphenyl)-1*H*-1,2,3-triazol-1-yl)-β-D-allofuranose (4c)

Treatment of compound **3c** (337 mg, 0.52 mmol) following general procedure D gave rise to the expected derivative **4c** as a white solid (214 mg, 90%).

Rf (CH_2Cl_2/MeOH: 9/1) 0.33. 1H NMR (300 MHz, DMSO) δ 8.65 (s, 1H, H-triazole), 7.97 – 7.61 (m, 2H, H-Ar), 7.03 (d, J = 8.8 Hz, 2H, H-Ar), 5.93 (d, J = 5.7 Hz, 1H, H-1’), 5.60 (d, J = 6.5 Hz, 1H, OH), 5.53 (d, J = 5.9 Hz, 1H, OH), 5.27 (d, J = 5.1 Hz, 1H, OH), 4.44 (d, J = 5.5 Hz, 1H, H-2’), 4.25 (d, J = 3.1 Hz, 1H, H-3’), 4.07 – 3.89 (m, 6H, H-4’, H-5’, O-C$\underline{H}_2$-CH_3), 3.80 (s, 3H, OCH_3), 2.18 – 1.81 (m, 2H, H-6’, H-6’’), 1.21 (td, J = 7.0, 3.0 Hz, 6H, O-CH_2-C$\underline{H}_3$). 13C NMR (75 MHz, DMSO) δ 159.7 (C-Ar),

147.2 (C=C), 127.1, 123.7 (C-Ar), 119.4 (C=CH), 114.9 (C-Ar), 92.3 (C-1'), 89.0 (d, J = 15.8 Hz, C-4'), 75.7 (C-2'), 70.0 (C-3'), 66.3 (d, J = 4.2 Hz, C-5'), 61.6, 61.3 (2d, J = 6.1 Hz, O-$\underline{C}H_2$-CH_3), 55.7 (OCH_3), 29.9 (d, J = 138.8 Hz, C-6'), 23.1, 16.8 (d, J = 6.0 Hz, O-CH_2-$\underline{C}H_3$). 31P NMR (81 MHz, DMSO) δ 29.15. MS ESI-QTof>0, m/z 458.17 (M+H) +, 459.17 (M+2H) +, 915.33(2M+H) +. HR-MS Calculated for $C_{19}H_{29}N_3O_8P$: 458.1692 (M+H) +; found: 458.1692.

6-Deoxy-6-phosphono-1-(4-(4-methoxyphenyl)-1*H*-1,2,3-triazol-1-yl)-β-D-allofuranose disodium salt (1c)

Treatment of compound **4c** (176 mg, 0.39 mmol) following general procedure E gave rise to the expected derivative **1c** as a white solid (170 mg, 99%).

Rf (iPrOH/NH_4OH/H_2O: 7/2/1) 0.14. 1H NMR (400 MHz, D_2O) δ 8.46 (s, 1H, H-triazole), 7.79 – 7.69 (m, 2H, H-Ar), 7.11 – 7.00 (m, 2H, H-Ar), 6.01 (d, J = 5.5 Hz, 1H, H-1'), 4.63 (t, J = 5.5 Hz, 1H, H-2'), 4.40 (dd, J = 5.4, 3.6 Hz, 1H, H-3'), 4.16 (ddd, J = 9.5, 7.7, 3.8 Hz, 2H, H-4', H-5'), 3.85 (s, 3H, OCH_3), 1.84 – 1.48 (m, 2H, H-6', H-6''). 13C NMR (100 MHz, D_2O) δ 159.2 (C-Ar), 147.6 (C=C), 127.3, 122.5 (C-Ar), 119.7 (C=CH), 114.6 (C-Ar), 93.1 (C-1'), 89.0 (d, J = 14.4 Hz (C-4'), 76.7 (C-2'), 70.1 (C-3'), 68.2 (d, J = 3.5 Hz, C-5'), 55.5 (OCH_3), 31.5 (d, J = 125.5 Hz, C-6'). 31P NMR (121 MHz, D_2O) δ 18.45. MS ESI-QTof>0, m/z 402.11 (M-2Na+3H) +. MS ESI-QTof <0, m/z 400.09 (M-2Na+H)-, 801.19 (2M-4Na+3H)-. HR-MS Calculated for $C_{15}H_{19}N_3O_8P$: 400.0910 (M-2Na+H)-; found: 400.0910. UV (H_2O) λmax = 251 nm (εmax = 17939).

3-*O*-Benzoyl-6-deoxy-2,5-*O*-diacetyl-6-diethylphosphono-1-(4-(3,5-dimethoxy-phenyl)-1*H*-1,2,3-triazol-1-yl)-β-D-allofuranose (3d)

Treatment of compound **2** (345 mg, 0.67 mmol) following general procedure A gave rise to the expected derivative **3d** as a white oil (420 mg, 93%).

Rf (CH_2Cl_2/AcOEt: 1/1) 0.29. 1H NMR (300 MHz, $CDCl_3$) δ 8.08 (dd, J = 8.4, 1.3 Hz, 2H, H-Ar), 7.92 (s, 1H, H-triazole), 7.68-7.60 (m, 1H, H-Ar), 7.49 (t, J = 7.6 Hz, 2H, H-Ar), 7.00 (d, J = 2.3 Hz, 2H, H-Ar), 6.46 (t, J = 2.3 Hz, 1H, H-Ar), 6.25 (d, J = 4.7 Hz, 1H, H-1'), 6.12-6.01 (m, 2H, H-2', H-3'), 5.51 (dtd, J = 10.6, 6.7, 3.9 Hz, 1H, H-5'), 4.77 (t, J = 3.9 Hz, 1H, H-4'), 4.15-4.04 (m, 4H, O-C$\underline{H}_2$-CH_3), 3.85 (s, 6H, OCH_3), 2.33-2.18 (m, 2H, H-6', H-6''), 2.04 (d, J = 5.5 Hz, 6H, H-Ac), 1.28 (td, J = 7.0, 2.9 Hz, 6H, O-CH_2-C$\underline{H}_3$). 13C NMR (75 MHz, $CDCl_3$) δ 169.7, 168.9 (C=O, Ac), 164.6 (C=O, Bz), 161.0 (C-Ar), 148.0 (C=C), 133.7, 131.5, 129.7, 128.5 (C-Ar), 119.3 (C=CH), 103.5, 100.7 (C-Ar), 89.6 (C-1'), 84.3 (d, J = 9.6 Hz, C-4'), 74.3 (C-3'), 70.5 (C-2'), 67.4 (C-5'), 62.1, 62.0 (2d, J = 6.8 Hz, O-$\underline{C}H_2$-CH_3), 55.3 (OCH_3), 27.2 (d, J = 153.5 Hz, C-6'), 20.9, 20.3 (CH_3, Ac), 16.3 (d, J = 6.0 Hz, O-CH_2-$\underline{C}H_3$). 31P NMR (81 MHz, $CDCl_3$) δ 24.95. MS ESI-QTof>0, m/z 676.23 (M+H) +, 677.23 (M+2H) +, 698.21 (M+Na) +. HR-MS Calculated for $C_{31}H_{39}N_3O_{12}P$: 676.2271 (M+H) +; found: 676.2268.

6-deoxy-6-diethylphosphono-1-(4-(3,5-dimethoxyphenyl)-1*H*-1,2,3-triazol-1-yl)-β-D-allofuranose (4d)

Treatment of compound **3d** (380 mg, 0.56 mmol) following general procedure D gave rise to the expected derivative **4d** as a white solid (261 mg, 95%).

Rf (CH_2Cl_2/MeOH: 9/1) 0.40. 1H NMR (300 MHz, DMSO) δ 8.79 (s, 1H, H-triazole), 7.06 (d, J = 2.3 Hz, 2H, H-Ar), 6.50 (t, J = 2.3 Hz, 1H, H-Ar), 5.94 (d, J = 5.7 Hz, 1H, H-1'), 5.63 (d, J = 6.5 Hz, 1H, OH), 5.53 (d, J = 5.9 Hz, 1H, OH), 5.29 (d, J = 5.1 Hz, 1H, OH), 4.45 (dd, J = 11.3, 5.7 Hz, 1H, H-2'), 4.26 (dd, J = 8.1, 5.0 Hz, 1H, H-3'), 4.08 – 3.88 (m, 6H, H-4', H-5', O-C$\underline{H}_2$-CH_3), 3.81 (s, 6H, OCH_3), 2.12 – 1.73 (m, 2H, H-6', H-6''), 1.22 (td, J = 7.0, 3.7 Hz, 6H, O-CH_2-C$\underline{H}_3$). 13C NMR (75 MHz, DMSO) δ 160.8 (C-Ar), 146.5 (C=C), 132.2 (C-Ar), 120.1 (C=CH), 103.1, 99.8 (C-Ar), 91.7 (C-

1'), 88.3 (d, J = 15.6 Hz, C-4'), 75.1 (C-2'), 69.3 (C-3'), 65.5 (d, J = 4.1 Hz, C-5'), 61.0., 60.7 (2d, J = 6.1 Hz, O-<u>C</u>H_2-CH_3), 55.2 (OCH_3), 29.2 (d, J = 138.7 Hz, C-6'), 16.1 (d, J = 6.0 Hz, O-CH_2-<u>C</u>H_3). 31P NMR (81 MHz, DMSO) δ 29.62. MS ESI-QTof>0, m/z 488.18 (M+H) +, 489.18 (M+2H) +, 975.35(2M+H) +. HR-MS Calculated for $C_{20}H_{31}N_3O_9P$: 488.1798 (M+H) +; found: 488.1802.

6-Deoxy-6-phosphono-1-(4-(3,5-dimethoxyphenyl)-1*H*-1,2,3-triazol-1-yl)-β-D-allofuranose (disodium salt) (1d)

Treatment of compound **4d** (220 mg, 0.45 mmol) following general procedure E gave rise to the expected derivative **1d** as a white solid (131 mg, 61%).

Rf (iPrOH/NH_4OH/ H_2O: 7/2/1) 0.19. 1H NMR (400 MHz, D_2O) δ 8.28 (s, 1H, H-triazole), 6.60 (s, 2H, H-Ar), 6.21 (s, 1H, H-Ar), 6.06 (d, J = 4.9 Hz, 1H, H-1'), 4.64 (t, J = 5.0 Hz, 1H, H-2'), 4.56 (t, J = 4.7 Hz, 1H, H-3'), 4.25 (ddd, J = 13.1, 8.1, .9 Hz, 2H, H-4', H-5'), 3.68 (s, 6H, OCH_3), 1.87 (dtd, J = 24.1, 15.3, 6.6 Hz, 2H, H-6', H-6''). 13C NMR (100 MHz, D_2O) δ 160.2 (C-Ar), 147.1 (C=C), 130.9 (C-Ar), 120.6 (C=CH), 103.5, 100.3 (C-Ar), 91.7 (C-1'), 88.2 (d, J = 13.5 Hz, C-4'), 75.3 (C-2'), 69.2 (C-3'), 67.3 (d, J = 2.5 Hz, C-5'), 55.3 (OCH_3), 31.5 (d, J = 130.0 Hz, C-6'). 31P NMR (121 MHz, D2O) δ 20.02. MS ESI-QTof>0, m/z 432.12 (M-2Na+3H) +, 476.08 (M+H) +. MS ESI-QTof <0, m/z 400.09 (M-2Na+H)-. HR-MS Calculated for $C_{16}H_{21}N_3O_9Na_2P$: 476.0811 (M+H) +; found: 476.0807. UV (H_2O) λmax = 210 nm (εmax = 41241).

3-*O*-Benzoyl-6-deoxy-2,5-diacetyl-6-diethylphosphono-1-(4-(4-fluorophenyl)-1*H*-1,2,3-triazol-1-yl)-β-D-allofuranose (3e)

Treatment of compound **2** (357 mg, 0.69 mmol) following general procedure A gave rise to the expected derivative **3e** as white oil (221 mg, 50%).

Rf (CH_2Cl_2/AcOEt: 1/1) 0.24. 1H NMR (300 MHz, $CDCl_3$) δ 8.12 – 8.04 (m, 2H, H-Ar), 7.91 (s, 1H, H-triazole), 7.86 – 7.75 (m, 2H, H-Ar), 7.68 – 7.59 (m, 1H, H-Ar), 7.49 (t,

J = 7.6 Hz, 2H, H-Ar), 7.20 – 7.05 (m, 2H, H-Ar), 6.25 (d, J = 4.7 Hz, 1H, H-1'), 6.02 (dt, J = 9.4, 5.4 Hz, 2H, H-2', H-3'), 5.57 – 5.45 (m, 1H, H-5'), 4.76 (t, J = 3.9 Hz, 1H, H-4'), 4.16 – 4.02 (m, 4H, O-C<u>H</u>$_2$-CH$_3$), 2.37 – 2.09 (m, 2H, H-6'. H-6''), 2.04, 2.03 (2s, 6H, Ac), 1.28 (td, J = 6.7, 2.7 Hz, 6H, O-CH$_2$-C<u>H</u>$_3$). 13C NMR (75 MHz, CDCl$_3$) δ 169.7, 169.2 (C=O, Ac), 164.8 (C=O, Bz), 162.8 (d, J = 247.9 Hz, C-Ar), 147.3 (C=CH), 133.8, 129.7, 128.7, 128.6, 127.5 (d, J = 8.2 Hz), 126.2 (d, J = 3.3 Hz, C-Ar), 118.8 (C=CH), 115.8 (d, J = 21.8 Hz, C-Ar), 89.6 (C-1'), 84.4 (d, J = 9.7 Hz, C-4'), 74.2 (C-2'), 70.69 (C-3'), 67.5 (C-5'), 62.2, 62.0 (2d, J = 7.4, 6.7 Hz, O-<u>C</u>H$_2$-CH$_3$), 27.3 (d, J = 141.7 Hz, C-6'), 20.9, 20.3 (CH$_3$, Ac), 16.3 (d, J = 6.1 Hz, O-CH$_2$-<u>C</u>H$_3$). 31P NMR (81 MHz, CDCl$_3$) δ 24.95. MS ESI-QTof>0, m/z 634.20 (M+H) +, 661.16 (M+Na+5H) +. HR-MS Calculated for $C_{29}H_{34}N_3O_{10}PF$: 634.1996 (M+H) +; found: 634.1967.

6-Deoxy-6-diethylphosphono-1-(4-(fluorophenyl)-1*H*-1,2,3-triazol-1-yl)-β-D-allofuranose (4e)

Treatment of compound **3e** (204 mg, 0.32 mmol) following general procedure D gave rise to the expected derivative **4e** as a white solid (124 mg, 87%).

Rf (CH_2Cl_2/MeOH: 9/1) 0.33. 1H NMR (300 MHz, DMSO) δ 8.75 (s, 1H, H-triazole), 7.95 – 7.86 (m, 2H, h-Ar), 7.35 – 7.26 (m, 2H, H-Ar), 5.94 (d, J = 5.7 Hz, 1H, H-1'), 5.61 (d, J = 6.4 Hz, 1H, OH), 5.52 (d, J = 5.9 Hz, 1H, OH), 5.28 (d, J = 5.1 Hz, 1H, OH), 4.44 (dd, J = 11.3, 5.7 Hz, 1H, H-2'), 4.25 (dd, J = 8.0, 4.9 Hz, 1H, H-3'), 4.05 – 3.90 (m, 6H, H-4', H-5', O-C<u>H</u>$_2$-CH$_3$), 2.09 – 1.79 (m, 2H, H-6', H-6''), 1.21 (td, J = 7.0, 3.3 Hz, 6H, O-CH$_2$-C<u>H</u>$_3$). 13C NMR (75 MHz, DMSO) δ 161.9 (d, J = 244.5 Hz, C-Ar), 145.8 (C=CH), 127.3 (d, J = 8.3 Hz), 127.1 (d, J = 3.0 Hz, C-Ar), 119.7 (C=CH), 115.9 (d, J = 21.7 Hz, C-Ar), 91.9 (C-1'), 88.5 (d, J = 15.8 Hz, C-4'), 75.3 (C-

2’), 69.5 (C-3’), 65.7 (d, J = 4.3 Hz, C-5’), 61.0, 60.7 (2d, J = 6.1 Hz, O-<u>C</u>H_2-CH_3), 29.4 (d, J = 138.7 Hz, C-6’), 16.2 (d, J = 6.0 Hz, O-CH_2-<u>C</u>H_3). 31P NMR (81 MHz, DMSO) δ 29.76. 19F NMR (282 MHz, DMSO) δ -113.88. MS ESI-QTof>0, m/z 446.15 (M+H) +, 468.13 (M+Na) +, 891.29 (2M+H) +, 913.27 (2M+Na) +. HR-MS Calculated for $C_{18}H_{26}N_3O_7PF$: 446.1492 (M+H) +; found: 446.1495.

6-Deoxy-6-phosphono-1-(4-(fluorophenyl)-1*H*-1,2,3-triazol-1-yl)-β-D-allo-furanose disodium salt (1e)

Treatment of compound **4e** (88 mg, 0.20 mmol) following general procedure E gave rise to the expected derivative **1e** as a white solid (79 mg, 91%).

Rf (iPrOH/NH_4OH/H_2O: 7/2/1) 0.19. 1H NMR (400 MHz, D_2O) δ 8.42 (s, 1H, H-triazole), 7.70 (dd, J = 8.1, 5.6 Hz, 2H, H-Ar), 7.16 (t, J = 8.8 Hz, 2H, H-Ar), 6.12 (d, J = 5.0 Hz, 1H, H-1’), 4.69 (t, J = 5.1 Hz, 1H, H-2’), 4.56 (t, J = 4.6 Hz, 1H, H-3’), 4.22 (ddd, J = 13.2, 8.2, 4.1 Hz, 2H, H-4’, H-5’), 1.99 – 1.71 (m, 2H, H-6’, H-6’’). 13C NMR (100 MHz, D_2O) δ 162.7 (d, J = 245.6 Hz, C-Ar), 147.7 (C=CH), 127.7 (d, J = 8.6 Hz), 125.5 (d, J = 3.0 Hz, C-Ar), 120.4 (C=CH), 115.9 (d, J = 22.1 Hz, C-Ar), 91.7 (C-1’), 88.4 (d, J = 13.8 Hz, C-4’), 75.1 (C-2’), 69.4 (C-3’), 67.4 (d, J = 2.8 Hz, C-5’), 31.5 (d, J = 129.5 Hz, C-6’). 31P NMR (121 MHz, D_2O) δ 19.82. 19F NMR (376 MHz, D_2O) δ -113.38. MS ESI-QTof>0, m/z 412.07 (M-Na+2H) +, 434.05 (M +H) +, 823.13 (2M-2Na+3H) +. HR-MS Calculated for $C_{14}H_{16}N_3O_7PFNa_2$: 434.0505 (M +H) +; found: 434.0504. UV (H_2O) λmax = 240 nm (εmax = 15581).

3-*O*-Benzoyl-6-deoxy-2,5-*O*-diacetyl-6-diethylphosphono-1-(4-(3,5-bis(trifluoromethyl)pheny)-1*H*-1,2,3-triazol-1-yl)-β-D-allofuranose (3f)

Treatment of compound **2** (272 mg, 0.53 mmol) following general procedure A gave rise to the expected derivative **3f** as a black solid (293 mg, 74%).

Rf (CH_2Cl_2/AcOEt: 1/1) 0.51. 1H NMR (300 MHz, $CDCl_3$) δ 8.33 (s, 2H, H-Ar), 8.22 (s, 1H, H-triazole), 8.14 – 8.03 (m, 2H, H-Ar), 7.86 (s, 1H, H-Ar), 7.70 – 7.57 (m, 1H, H-Ar), 7.50 (t, J = 7.6 Hz, 2H, H-Ar), 6.29 (d, J = 4.9 Hz, 1H, H-1'), 6.07 – 5.94 (m, 2H, H-2', H-3'), 5.60 – 5.46 (m, 1H, H-5'), 4.76 (t, J = 4.0 Hz, 1H, H-4'), 4.23 – 4.01 (m, 4H, O-$C\underline{H}_2$-CH_3), 2.40 – 2.12 (m, 2H, H-6', H-6''), 2.08 (s, 3H, H-Ac), 2.04 (s, 3H, H-Ac), 1.33 – 1.24 (m, 6H, O-CH_2-$C\underline{H}_3$). 13C NMR (75 MHz, $CDCl_3$) δ 169.9, 169.4 (C=O, Ac), 165.1 (C=O, Bz), 145.8 (C=CH), 134.1, 132.7, 132.4, 132.3, 129.9, 128.9, 125.9 (C-Ar), 123.3 (d, J = 272.3 Hz, CF_3), 120.6 (C=CH), 90.2 (C-1'), 84.8 (d, J = 9.8 Hz, C-4'), 74.5 (C-2'), 70.8 (C-3'), 67.7 (C-5'), 62.4,62.2 (2d, J = 6.6 Hz, O-$\underline{C}H_2$-CH_3), 27.7 (d, J = 141.7 Hz, C-6'), 21.1, 20.5 (CH_3, Ac), 16.5 (d, J = 6.1 Hz, O-CH_2-$\underline{C}H_3$). 31P NMR (81 MHz, $CDCl_3$) δ 25.43. MS ESI-QTof>0, m/z 752.18 (M+H) +, 753.18 (M+2H) +. HR-MS Calculated for $C_{31}H_{33}N_3O_{10}F_6P$: 752.1808 (M+H) +; found: 752.1802.

6-Deoxy-6-ditehylphosphono-1-(4-(3,5-bis(trifluoromethyl)phenyl)-1*H*-1,2,3-triazol-1-yl)-β-D-allofuranose (4f)

Treatment of compound **3f** (250 mg, 0.33 mmol) following general procedure E gave rise to the expected derivative **4f** as a white solid (186 mg, 100%).

Rf (CH_2Cl_2/MeOH: 9/1) 0.32. 1H NMR (300 MHz, DMSO) δ 9.12 (s, 1H, H-Ar), 8.52 (s, 2H, H-Ar), 8.09 (s, 1H, H-triazole), 5.97 (d, J = 5.5 Hz, 1H, H-1'), 5.66 (d, J = 6.4 Hz, 1H, OH), 5.48 (d, J = 5.8 Hz, 1H, OH), 5.30 (d, J = 5.3 Hz, 1H, OH), 4.46 (dd, J = 11.2, 5.6 Hz, 1H, H-2'), 4.26 (dd, J = 8.2, 5.0 Hz, 1H, H-3'), 4.04 – 3.89 (m, 6H, H-4', H-5', O-$C\underline{H}_2$-CH_3), 2.09 – 1.79 (m, 2H, H-6', H-6''), 1.25 – 1.13 (m, 6H, O-CH_2-$C\underline{H}_3$). 13C NMR (75 MHz, DMSO) δ 144.0 (C=CH), 133.2, 131.3, 130.9, 125.4 (C-Ar), 123.3 (d, J = 272.9 Hz, CF3), 121.8 (C=CH), 92.1 (C-1'), 88.5 (d, J = 15.5 Hz, C-4'),

75.3 (C-2’), 69.4 (C-3’), 65.5 (d, J = 4.0 Hz, C-5’), 61.0, 60.7 (2d, J = 6.1 Hz, O-C̲H$_2$-CH$_3$), 29.3 (d, J = 139.3 Hz, C-6’), 16.2 (d, J = 6.0 Hz, O-CH$_2$-C̲H$_3$). 31P NMR (81 MHz, DMSO) δ 29.63. MS ESI-QTof>0, m/z 564.13 (M+H) +, 586.12 (M+Na) +. HR-MS Calculated for $C_{20}H_{25}N_3O_7F_6P$: 564.1334 (M+H) +; found: 564.1338.

6-Deoxy-6-phosphono-1-(4-(3,5-bis(trifluoromethyl)phenyl)-1*H*-1,2,3-triazol-1-yl)-β-D-allofuranose (disodium salt) (1f)

Treatment of compound **4f** (146 mg, 0.26 mmol) following general procedure G gave rise to the expected derivative **1f** as a white solid (139 mg, 96%).

Rf (iPrOH/NH_4OH/H_2O: 7/2/1) 0.17. 1H NMR (400 MHz, D_2O) δ 8.67 (s, H-triazole), 8.22 (s, 2H, H-Ar), 7.97 (s, H-Ar), 6.18 (s, 1H, H-1’), 4.73 (d, J = 3.3 Hz, 1H, H-2’), 4.58 (d, J = 2.7 Hz, 1H, H-3’), 4.38 – 4.27 (m, 1H, H-4’), 4.26 – 4.15 (m, 1H, H-5’), 1.87 – 1.58 (m, 2H, H-6’, H-6’’). 13C NMR (100 MHz, D_2O) 145.4 (C=CH), 131.7, 131.3 (C-Ar), 125.9, 123.3 (d, J = 272.2 Hz, CF3), 122.4, 121.7 (C-Ar), 119.2 (C=CH), 91.8 (C-1’), 88.7 (d, J = 13.3 Hz, C-4’), 75.3 (C-2’), 69.2 (C-3’), 67.8 (C-5’), 31.7 (d, J = 125.1 Hz, C-6’). 31P NMR (121 MHz, D_2O) δ 18.00. 19F NMR (376 MHz, D_2O) δ -62.84. MS ESI-QTof>0, m/z 508.07 (M-2Na+3H) +, 530.05 (M-Na+2H) +. MS ESI-QTof <0, m/z 506.04 (M-2Na+H)-. HR-MS Calculated for $C_{16}H_{15}N_3O_7F_6P$: 506.0552 (M-2Na+H)-; found: 506.0548. UV (H_2O) λ1max = 203 nm (εmax = 25060); λ2max = 247 nm (εmax = 14596).

3-*O*-Benzoyl-6-deoxy-2,5-di-*O*-acetyl-6-diethylphosphono-1-(4-(4-(dimethylamino) phenyl)-1*H*-,1,2,3-triazol-1-yl)-β-D-allofuranose (3g)

Treatment of compound **2** (328 mg, 0.64 mmol) following general procedure A gave rise to the expected derivative **3g** as an orange solid (370 mg, 88%).

Rf (CH_2Cl_2/AcOEt: 1/1) 0.26. 1H NMR (300 MHz, $CDCl_3$) δ 8.12 – 8.05 (m, 2H, H-Ar), 7.78 (s, 1H, H-triazole), 7.72 – 7.67 (m, 2H, H-Ar), 7.66 – 7.59 (m, 1H, H-Ar), 7.49 (t,

J = 7.6 Hz, 2H, H-Ar), 6.77 (d, J = 8.9 Hz, 2H, H-Ar), 6.23 (d, J = 4.6 Hz, 1H, H-1’), 6.02 (dt, J = 9.4, 5.3 Hz, 2H, H-2’, H-3’), 5.51 (qd, J = 6.7, 3.8 Hz, 1H, H-5’), 4.76 (t, J = 3.9 Hz, 1H, H-4’), 4.17 – 3.99 (m, 4H, O-C<u>H</u>$_2$-CH$_3$), 3.00 (s, 6H, N-CH$_3$), 2.37 – 2.10 (m, 2H, H-6’, H-6’’), 2.04, 2.03(2s, 6H, Ac), 1.27 (dt, J = 7.1, 3.5 Hz, 6H, O-CH$_2$-C<u>H</u>$_3$). 13C NMR (75 MHz, CDCl$_3$) δ 169.9, 169.4 (C=O, Ac), 165.0 (C=O, Bz), 150.7 (C-Ar), 148.9 (C=CH), 133.9, 129.9, 129.0, 128.8, 126.9 (C-Ar), 118.2 (C=CH), 117.6, 112.6 (C-Ar), 89.7 (C-1’), 84.4 (d, J = 9.4 Hz, C-4’), 74.4 (C-2’), 70.8 (C-3’), 67.8 (C-5’), 62.4, 62.3 (2d, J = 6.8 Hz, O-<u>C</u>H$_2$-CH$_3$), 40.5 (N-CH$_3$), 27.4 (d, J = 141.4 Hz, C-6’), 21.1, 20.5 (CH$_3$, Ac), 16.5 (d, J = 6.1 Hz, O-CH$_2$-<u>C</u>H$_3$). 31P NMR (81 MHz, CDCl$_3$) δ 25.19. MS ESI-QTof>0, m/z 659.25 (M+H) +. HR-MS Calculated for $C_{31}H_{40}N_4O_{10}P$: 659.2482 (M+H) +; found: 659.2484.

6-Deoxy-6-diethyphosphono-1-(4-(4-(dimethylamino)phenyl)-1*H*-1,2,3-triazol-1-yl)-β-D-allofuranose (4g)

Treatment of compound **3g** (330 mg, 0.50 mmol) following general procedure D gave rise to the expected derivative **4g** as a rose solid (234 mg, 99%).

Rf (CH_2Cl_2/MeOH: 9/1) 0.38. 1H NMR (300 MHz, DMSO) δ 8.53 (s, 1H, H-triazole), 7.67 (d, J = 8.9 Hz, 2H, H-Ar), 6.78 (d, J = 9.0 Hz, 2H, H-Ar), 5.91 (d, J = 5.8 Hz, 1H, H-1’), 5.54 (dd, J = 16.4, 6.2 Hz, 2H, OH), 5.25 (d, J = 5.1 Hz, 1H, OH), 4.43 (dd, J = 11.4, 5.9 Hz, 1H, H-2’), 4.24 (dd, J = 8.0, 5.0 Hz, 1H, H-3’), 4.07 – 3.87 (m, 6H, H-4’, H-5’, O-C<u>H</u>$_2$-CH$_3$), 2.93 (s, 6H, NCH$_3$), 2.11 – 1.79 (m, 2H, H-6’, H-6’’), 1.27 – 1.14 (m, 6H, O-CH$_2$-C<u>H</u>$_3$). 13C NMR (75 MHz, DMSO) δ 150.4 (C-Ar), 147.6 (C=CH), 126.4 (C-Ar), 118.7 (C=CH), 118.0, 112.6 (C-Ar), 91.7 (C-1’), 88.6 (d, J = 8.7 Hz, C-4’), 75.4 (C-2’), 69.8 (C-3’), 66.0 (d, J = 4.2 Hz, C-5’), 61.3, 61.1 (2d, J = 6.2 Hz, O-<u>C</u>H$_2$-CH$_3$), 55.2 (N-CH$_3$), 29.7 (d, J = 138.7 Hz, C-6’), 16.5 (d, J = 6.0 Hz, O-CH$_2$-

$\underline{C}H_3$). 31P NMR (81 MHz, DMSO) δ 29.60. MS ESI-QTof>0, m/z 471.20 (M+H) +. HR-MS Calculated for $C_{20}H_{32}N_4O_7P$: 471.2009 (M+H) +; found: 471.2009.

6-Deoxy-6-phosphono-1-(4-*N,N*-dimethylanilin-1*H*-1,2,3-triazol-1-yl)-β-D-allo-furanose (disodium salt) (1g)

Treatment of compound **4g** (194 mg, 0.41 mmol) following general procedure E gave rise to the expected derivative **1g** as a white solid (166 mg, 88%).

Rf (iPrOH/NH_4OH/H_2O: 7/2/1) 0.15. 1H NMR (400 MHz, D_2O) δ 8.29 (s, 1H, H-triazole), 7.59 (d, J = 8.6 Hz, 2H, H-Ar), 6.94 (d, J = 8.7 Hz, 2H, H-Ar), 6.07 (d, J = 5.0 Hz, 1H, H-1'), 4.67 (t, J = 5.2 Hz, 1H, H-2'), 4.55 (t, J = 4.6 Hz, 1H, H-3'), 4.21 (ddd, J = 13.6, 8.3, 4.2 Hz, 2H, H-4', H-5'), 2.86 (s, 6H, N-CH_3), 2.04 – 1.64 (m, 2H, H-6', H-6''). 13C NMR (100 MHz, D_2O) δ 151.3 (C-Ar), 147.9 (C=CH), 126.7, 119.7 (C=CH), 119.4, 115.1 (C-Ar), 91.6 (C-1'), 88.3 (d, J = 13.9 Hz, C-4'), 75.1 (C-2'), 69.4 (C-3'), 67.3 (d, J = 3.1 Hz, C-5'), 40.8 (N-CH_3), 31.4 (d, J = 131.2 Hz, C-6'). 31P NMR (121 MHz, D_2O) δ 20.14. MS ESI-QTof>0, m/z 415.14 (M-2Na+3H) +, 829.27 (2M-4Na+5H) +. MS ESI-QTof <0, m/z 413.12 (M-2Na+H)-, 827.25 (2M-4Na+3H)-. HR-MS Calculated for $C_{16}H_{22}N_4O_7P$: 413.1226 (M-2Na+H)-; found: 413.1226. UV (H_2O) λmax = 24656 nm (εmax = 277).

3-*O*-Benzoyl-6-deoxy-2,5-diacetyl-6-diethylphosphono-1-(4-(2-aminophenyl)-1*H*-1,2,3-triazol-1-yl)-β-D-allofuranose (3h)

Treatment of compound **2** (400 mg, 0.78 mmol) following general procedure C gave rise to the expected derivative **3h** as brown solid (389 mg, 79%).

Rf (CH_2Cl_2/AcOEt: 1/1) 0.21. 1H NMR (400 MHz, $CDCl_3$) δ 8.09 (dd, J = 8.2, 1.1 Hz, 2H, H-Ar), 7.94 (s, 1H, H-triazole), 7.63 (t, J = 7.5 Hz, 1H, H-Ar), 7.49 (t, J = 7.7 Hz, 2H, H-Ar), 7.37 (dd, J = 7.7, 1.1 Hz, 1H, H-Ar), 7.14 (t, J = 7.7 Hz, 1H, H-Ar), 6.75 (dd, J = 15.8, 7.9 Hz, 2H, H-Ar), 6.26 (d, J = 4.7 Hz, 1H, H-1'), 6.07 (t, J = 5.0 Hz, 1H,

H-2’), 6.03 – 5.98 (m, 1H, H-3’), 5.52 (ddd, J = 18.6, 6.7, 4.0 Hz, 1H, H-5’), 4.78 (t, J = 4.1 Hz, 1H, H-4’), 4.16 – 3.92 (m, 2H, H-6’, H-6’’), 2.24 (dddd, J = 41.2, 19.2, 15.5, 6.7 Hz, 4H, O-C<u>H</u>$_2$-CH$_3$), 2.04, 2.03 (2s, 6H, Ac), 1.28 (td, J = 7.1, 4.0 Hz, 6H, O-CH$_2$-C<u>H</u>$_3$). 13C NMR (100 MHz, CDCl$_3$) δ 169.9, 169.4 (C=O, Ac), 165.1 (C=O, Bz), 148.9 (C=CH), 145.2, 133.9, 129.9, 129.5, 128.8, 128.0 (C-Ar), 119.6 (C=CH), 117.7, 116.9, 113.28 (C-Ar), 89.9 (C-1’), 84.5 (d, J = 9.6 Hz, C-4’), 74.4 (C-2’), 70.8 (C-3’), 67.7 (C-5’), 62.4, 62.3 (2d, J = 6.5 Hz, O-<u>C</u>H$_2$-CH$_3$), 27.5 (d, J = 141.7 Hz, C-6’), 21.1, 20.5 (CH$_3$-Ac), 16.5 (d, J = 6.0 Hz, O-CH$_2$-<u>C</u>H$_3$). 31P NMR (121 MHz, CDCl$_3$) δ 24.76. MS ESI-QTof>0, m/z 631.22 (M+H) +, 632.22 (M+2H) +. HR-MS Calculated for $C_{29}H_{36}N_4O_{10}P$: 631.2169 (M+H) +; found: 631.2169.

6-Deoxy-6-diethylphosphono-1-(4-(2-aminophenyl)-1*H*-1,2,3-triazol-1-yl)-β-D-allofuranose (4h)

Treatment of compound **3h** (349 mg, 0.55 mmol) following general procedure D gave rise to the expected derivative **4h** as a yellow oil (204 mg, 83%).

Rf (CH$_2$Cl$_2$/MeOH: 9/1) 0.38. 1H NMR (300 MHz, DMSO) δ 8.69 (s, 1H, H-triazole), 7.50 (dd, J = 7.8, 1.4 Hz, 1H, H-Ar), 7.12 – 6.97 (m, 1H, H-Ar), 6.85 – 6.71 (m, 1H, H-Ar), 6.61 (t, J = 7.4 Hz, 1H, H-Ar), 6.15 (d, J = 7.0 Hz, 1H, NH$_2$) ,5.97 (d, J = 5.6 Hz, 1H, H-1’), 5.62 (d, J = 6.3 Hz, 1H, OH), 5.55 (d, J = 5.8 Hz, 1H, OH), 5.28 (d, J = 5.0 Hz, 1H, OH), 4.47 (dd, J = 10.2, 5.0 Hz, 1H, H-2’), 4.37 – 4.19 (m, 1H, H-3’), 4.10 – 3.86 (m, 6H, H-4’, H-5’, O-C<u>H</u>$_2$-CH$_3$), 2.11 – 1.79 (m, 2H, H-6’, H-6’’), 1.22 (td, J = 7.0, 2.4 Hz, 6H, O-CH$_2$-C<u>H</u>$_3$). 13C NMR (75 MHz, DMSO) δ 147.7 (C=C), 145.7, 132.1, 128.7, 127.7 (C-Ar), 119.6 (C=CH), 116.0, 115.8, 112.5 (C-Ar), 91.9 (C-1’), 88.4 (d, J = 14.9 Hz, C-4’), 75.3 (C-2’), 69.4 (C-3’), 65.6 (d, J = 3.8 Hz, C-5’), 61.0, 60.7 (2d, J = 6.0 Hz, O-<u>C</u>H$_2$-CH$_3$), 29.4 (d, J = 138.5 Hz, C-6’), 16.2 (d, J = 5.9 Hz,

O-CH_2-$\underline{C}H_3$). 31P NMR (81 MHz, DMSO) δ 29.56. MS ESI-QTof>0, m/z 443.17 (M+H) +, 444.17 (M+2H) +. HR-MS Calculated for $C_{18}H_{28}N_4O_7P$: 443.1696 (M+H) +; found: 443.1692.

6-Deoxy-6-phosphono-1-(4-(2-aminophenyl)-1H-1,2,3-triazol-1-yl)-β-D-allo-furanose (disodium salt) (1h)

Treatment of compound **4h** (164 mg, 0.37 mmol) following general procedure E gave rise to the expected derivative **1h** as a white solid (108 mg, 68%).

Rf (iPrOH/NH_4OH/H_2O: 7/2/1) 0.12. 1H NMR (400 MHz, D_2O) δ 8.44 (s, 1H, H-triazole), 7.48 (dd, J = 7.7, 1.4 Hz, 1H, H-Ar), 7.34 – 7.19 (m, 1H, H-Ar), 7.04 – 6.81 (m, 2H, H-Ar), 6.17 (d, J = 5.1 Hz, 1H, H-1'), 4.74 (t, J = 5.2 Hz, 1H, H-2'), 4.62 – 4.54 (m, 1H, H-3'), 4.28 (t, J = 3.9 Hz, 1H, H-4'), 4.20 (ddd, J = 13.1, 8.9, 4.5 Hz, 1H, H-5'), 1.83 (dddd, J = 23.9, 16.3, 14.9, 6.8 Hz, 2H, H-6', 6''). 13C NMR (100 MHz, D_2O) δ 146.5 (C=C), 143.8, 129.9, 129.0, 121.6, 119.4 (C-Ar), 117.7 (C=CH), 115.3 (C-Ar), 91.5 (C-1),88.3 (d, J = 13.8 Hz, C-4'), 75.0 (C-2'), 69.2 (C-3'), 67.3 (d, J = 2.9 Hz, C-5'), 31.4 (d, J = 129.1 Hz, C-6'). 31P NMR (121 MHz, D_2O) δ 19.55. HR-MS Calculated for $C_{14}H_{20}N_4O_7P$: 387.1070 (M-2Na+3H) +; found: 387.1072. UV (H_2O) λ1max = 213 nm (ε1max = 29180), λ2max = 302 nm (ε2max = 3591).

3-*O*-Benzoyl-6-deoxy-2,5-di-*O*-acetyl-6-diethylphosphono-1-(4-(3-aminophenyl)-1*H*-1,2,3-triazol-1-yl)-β-D-allofuranose (3i)

Treatment of compound **2** (394 mg, 0.77 mmol) following general procedure C gave rise to the expected derivative **3i** as white solid (416 mg, 86%).

Rf (CH_2Cl_2/AcOEt: 1/1) 0.11. 1H NMR (300 MHz, $CDCl_3$) δ 8.24 – 7.96 (m, 2H, H-Ar), 7.89 (s, 1H, H-triazole), 7.72 – 7.58 (m, 1H, H-Ar), 7.50 (dd, J = 10.5, 4.7 Hz, 2H, H-Ar), 7.17 (tt, J = 4.4, 2.6 Hz, 3H, H-Ar), 6.68 (ddd, J = 7.7, 2.3, 1.2 Hz, 1H, H-Ar), 6.25 (d, J = 4.7 Hz, 1H, H-1'), 6.04 (dt, J = 9.5, 5.4 Hz, 2H, H-2', H-3'), 5.52 (dt, J =

6.7, 2.9 Hz, 1H, H-5'), 4.78 (t, J = 3.9 Hz, 1H, H-4'), 4.26 – 3.97 (m, 4H, O-$C\underline{H}_2$-CH_3), 3.80 (s, 2H, NH_2), 2.25 (dqd, J = 19.0, 15.5, 6.7 Hz, 2H, H-6', H-6''), 2.04 (d, J = 2.8 Hz, 6H, CH_3-Ac), 1.28 (ddd, J = 6.9, 4.9, 2.1 Hz, 6H, O-CH_2-$C\underline{H}_3$). 13C NMR (75 MHz, $CDCl_3$) δ 170.1, 169.5 (C=O, Ac), 165.2 (C=O, Bz), 148.6 (C=C), 147.2, 134.1, 131.1, 130.1, 130.0, 129.0, 128.9 (C-Ar), 119.5 (C=CH), 116.4, 115.5, 112.7 (C-Ar), 89.9 (C-1'), 84.6 (d, J = 9.6 Hz, C-4'), 74.5 (C-3'), 70.9 (C-2'), 67.8 (C-5'), 62.4, 62.3 (2d, J = 6.7 Hz, O-$\underline{C}H_2$-CH_3), 27.6 (d, J = 141.3 Hz, C-6'), 21.2, 20.6 (CH_3, Ac), 16.6 (d, J = 6.1 Hz, O-CH_2-$\underline{C}H_3$). 31P NMR (81 MHz, $CDCl_3$) δ 25.14. MS ESI-QTof>0, m/z 631.22 (M+H) +, 653.20 (M+Na) +, 1261.42 (2M+H) +. HR-MS Calculated for $C_{29}H_{36}N_4O_{10}P$: 631.2169 (M+H) +; found: 631.2168.

6-Deoxy-6-diethylphosphono-1-(4-(3-aminophenyl)-1*H*-1,2,3-triazol-1-yl)-β-D-allofuranose (4i)

Treatment of compound **3i** (376 mg, 0.60 mmol) following general procedure D gave rise to the expected derivative **4i** as a brown solid (250 mg, 95%).

Rf (CH_2Cl_2/MeOH: 9/1) 0.20. 1H NMR (300 MHz, DMSO) δ 8.60 (s, 1H, H-triazole), 7.19 – 7.02 (m, 2H, H-Ar), 7.01 – 6.89 (m, 1H, H-Ar), 6.55 (ddd, J = 7.9, 2.2, 0.9 Hz, 1H, H-Ar), 5.94 (d, J = 5.7 Hz, 1H, H-1'), 5.59 (d, J = 6.5 Hz, 1H, OH), 5.53 (d, J = 5.8 Hz, 1H, OH), 5.26 (d, J = 5.1 Hz, 1H, OH), 5.19 (s, 2H, NH_2), 4.44 (dd, J = 11.4, 5.8 Hz, 1H, H-2'), 4.26 (dt, J = 8.1, 4.1 Hz, 1H, H-3'), 4.11 – 3.79 (m, 6H, H-4', H-5', O-$C\underline{H}_2$-CH_3), 2.15 – 1.80 (m, 2H, H-6', H-6''), 1.22 (td, J = 7.0, 2.1 Hz, 6H, O-CH_2-$C\underline{H}_3$). 13C NMR (75 MHz, DMSO) δ 149.1 (C=C), 147.4, 130.9, 129.3 (C-Ar), 119.4 (C=CH), 113.7, 113.1, 110.5 (C-Ar), 91.7 (C-1'), 88.4(d, J = 15.4 Hz, C-4'), 75.2 (C-2'), 69.4 (C-3'), 65.7 (d, J = 4.2 Hz, C-5'), 61.2, 60.9 (2d, J = 6.2 Hz, O-$\underline{C}H_2$-CH_3), 29.4 (d, J = 138.6 Hz, C-6'), 16.2 (d, J = 6.0 Hz, O-CH_2-$\underline{C}H_3$). 31P NMR (81 MHz,

DMSO) δ 29.53. MS ESI-QTof>0, m/z 443.17 (M+H) +, 885.33 (2M+H) +. HR-MS Calculated for $C_{18}H_{28}N_4O_7P$: 443.1696 (M+H) +; found: 443.1695.

6-Deoxy-6-phosphono-1-(4-(3-aminophenyl)-1*H*-1,2,3-triazol-1-yl)-β-D-allo-furanose disodium salt (1i)

Treatment of compound **4i** (210 mg, 0.47 mmol) following general procedure E gave rise to the expected derivative **1i** as a white solid (97 mg, 48%).

Rf (iPrOH/NH_4OH/H_2O: 7/2/1) 0.13. 1H NMR (400 MHz, D_2O) δ 8.34 (s, 1H, H-triazole), 7.24 (t, J = 7.9 Hz, 1H, H-Ar), 7.16 – 7.08 (m, 2H, H-Ar), 6.86 – 6.73 (m, 1H, H-Ar), 6.07 (d, J = 5.0 Hz, 1H, H-1’), 4.65 (t, J = 5.1 Hz, 1H, H-2’), 4.58 – 4.48 (m, 1H, H-3’), 4.27 – 4.02 (m, 2H, H-4’, H-5’), 2.03 – 1.55 (m, 2H, H-6’, H-6’’). 13C NMR (100 MHz, D_2O) δ 147.7 (C=C), 146.8, 130.2, 120.6 (C-Ar), 116.8 (C=CH), 113.2 (C-Ar), 91.7 (C-1’), 88.3 (d, J = 13.8 Hz, C-4’), 75.1 (C-2’), 69.3 (C-3’), 67.3 (d, J = 2.1 Hz, C-5’), 31.4 (d, J = 130.2 Hz, C-6’). 31P NMR (121 MHz, D_2O) δ 19.64. MS ESI-QTof>0, m/z 387.11 (M-2Na+3H) +, 409.09 (M-Na+2H) +, 431.07 (M+H) +, 773.21 (2M-4Na+5H) +, 795.19 (2M-3Na+4H) +, MS ESI-QTof <0, m/z 385.08 (M-2Na+H)-, 386.08 (M-2Na+2H)-, 771.17 386.08 (2M-4Na+3H)-. HR-MS Calculated for $C_{14}H_{20}N_4O_7P$: 387.1070 (M-2Na+3H) +; found: 387.1070. UV (H_2O) λmax = 224 nm (εmax = 29345).

3-*O*-Benzoly-6-deoxy-2,5-di-*O*-acetyl-6-diethylphosphono-1-(4-(4-aminophenyl)-1*H*-1,2,3-triazol-1-yl)-β-D-allofuranose (3j)

Treatment of compound **2** (380 mg, 0.74 mmol) following general procedure C gave rise to the expected derivative **3j** as yellow solid (359 mg, 77%).

Rf (CH_2Cl_2/AcOEt: 1/1) 0.14. 1H NMR (300 MHz, $CDCl_3$) δ 8.09 – 8.02 (m, 2H, H-Ar), 7.78 (s, 1H, H-triazole), 7.66 – 7.56 (m, 3H, H-Ar), 7.49 (t, J = 7.6 Hz, 2H, H-Ar), 6.76 – 6.68 (m, 2H, H-Ar), 6.23 (d, J = 4.7 Hz, 1H, H-1’), 6.08 – 5.88 (m, 2H, H-2’, H-3’),

5.57 – 5.42 (m, 1H, H-5’), 4.74 (t, J = 3.9 Hz, 1H, H-4’), 4.14 – 3.96 (m, 4H, O-C$\underline{H}_2$-CH_3), 3.80 (s, 2H, NH_2), 2.39 – 2.07 (m, 2H, H-6’, H6’’), 2.03 (d, J = 5.0 Hz, 6H, CH_3-Ac), 1.27 (td, J = 7.1, 2.0 Hz, 6H, O-CH_2-C$\underline{H}_3$). 13C NMR (75 MHz, $CDCl_3$) δ 169.9 169.4 (C=O, Ac), 165.1 (C=O, Bz), 148.7 (C=C), 146.9, 133.9, 129.9, 128.9, 128.8, 127.2 (C-Ar), 120.5 (C=CH), 117.8, 115.4 (C-Ar), 89.7 (C-1’), 84.4 (d, J = 9.6 Hz, C-4’), 74.3 (C-3’), 70.8 (C-2’), 67.7 (C-5’), 62.4, 62.2 (2d, J = 6.7 Hz, O-$\underline{C}H_2$-CH_3), 27.4 (d, J = 141.6 Hz), 21.1, 20.5 (CH_3, Ac), 16.5 (d, J = 6.1 Hz, O-CH_2-$\underline{C}H_3$). 31P NMR (81 MHz, $CDCl_3$) δ 25.22. MS ESI-QTof>0, m/z 631.22 (M+H) +, 1261.42 (2M+H) +. HR-MS Calculated for $C_{29}H_{36}N_4O_{10}P$: 631.2169 (M+H) +; found: 631.2165.

6-Deoxy-6-diethylphosphono-1-(4-(4-aminophenyl)-1*H*-1,2,3-triazol-1-yl)-β-D-allofuranose (4j)

Treatment of compound **3j** (319 mg, 0.50 mmol) following general procedure D gave rise to the expected derivative **4j** as white solid (199 mg, 90%).

Rf (CH_2Cl_2/MeOH: 9/1) 0.21. 1H NMR (300 MHz, DMSO) δ 8.46 (s, 1H, H-triazole), 7.52 (d, J = 8.5 Hz, 2H, H-Ar), 6.63 (d, J = 8.5 Hz, 2H, H-Ar), 5.90 (d, J = 5.7 Hz, 1H, H-1’), 5.56 (d, J = 6.5 Hz, 1H, OH), 5.51 (d, J = 5.9 Hz, 1H, OH), 5.24 (s, 2H, NH_2), 4.42 (dd, J = 11.4, 5.8 Hz, 1H, H-2’), 4.25 (dd, J = 8.0, 5.0 Hz, 1H, H-3’), 4.08 – 3.78 (m, 6H, H-4’, H-5’, O-C$\underline{H}_2$-CH_3), 2.13 – 1.79 (m, 2H, H-6’, H-6’’), 1.22 (td, J = 7.0, 2.4 Hz, 6H, O-CH_2-C$\underline{H}_3$). 13C NMR (75 MHz, DMSO) δ 148.9 (C=C), 147.9, 126.4 (C-Ar), 118.3 (C=CH), 117.6, 114.1 (C-Ar), 91.8 (C-1’), 88.5 (d, J = 15.6 Hz, C-4’), 75.3 (C-2’), 69.7 (C-3’), 65.9 (d, J = 3.8 Hz, C-5’), 61.3, 61.0 (2d, J = 6.1 Hz, O-$\underline{C}H_2$-CH_3), 29.6 (d, J = 138.6 Hz, C-6’), 16.4 (d, J = 6.0 Hz, O-CH_2-$\underline{C}H_3$). 31P NMR (81 MHz, DMSO) δ 29.60. MS ESI-QTof>0, m/z 443.17 (M+H) +, 885.33 (2M+H) +. HR-MS Calculated for $C_{18}H_{28}N_4O_7P$: 443,1696 (M+H) +; found: 443.1696.

6-Deoxy-6-phosphono-1-(4-(4-aminophenyl)-1*H*-1,2,3-triazol-1-yl)-β-D-allo-furanose disodium salt (1j)

Treatment of compound **4j** (159 mg, 0.36 mmol) following general procedure E gave rise to the expected derivative **1j** as a white solid (90 mg, 59%).

Rf (iPrOH/NH_4OH/H_2O: 7/2/1) 0.11. 1H NMR (400 MHz, D_2O) δ 8.26 (s, 1H, H-triazole), 7.51 (d, J = 8.6 Hz, 2H, H-Ar), 6.84 (d, J = 8.6 Hz, 2H, H-Ar), 6.06 (d, J = 5.1 Hz, 1H, H-1'), 4.64 (t, J = 5.2 Hz, 1H, H-2'), 4.56 – 4.45 (m, 1H, H-2'), 4.32 – 3.94 (m, 2H, H-4', H-5'), 2.03 – 1.60 (m, 2H, H-6', H-6''). 13C NMR (100 MHz, D_2O) δ 148.1 (C=C), 147.0, 126.9, 120.3, 119.4 (C-Ar), 116.6 (C=CH), 91.6 (C-1'), 88.3 (d, J = 13.8 Hz, C-4'), 75.0 (C-2'), 69.3 (C-3'), 67.3 (d, J = 1.7 Hz, C-5'), 31.4 (d, J = 129.9 Hz, C-6'). 31P NMR (121 MHz, D_2O) δ 20.04. MS ESI-QTof>0, m/z 387.11 (M-2Na+3H) +, 409.09 (M-Na+2H) +. MS ESI-QTof <0, m/z 388.06 (M-2Na+4H)-, 389.06 (M-2Na+5H)-. HR-MS Calculated for $C_{14}H_{20}N_4O_7P$: 387.1070 (M-2Na+3H) +; found: 387.1069. UV (H_2O) λ1max = 204 nm (ε1max = 31772); λ2max = 265 nm (ε2max = 23675).

3-*O*-Benzoyl-6-deoxy-2,5-di-*O*-acetyl-6-diethylphosphono-1-(4-(3-formylphenyl)-1H-1,2,3-triazol-1-yl)-β-D-allofuranose (3k)

Treatment of compound **2** (385 mg, 0.75 mmol) following general procedure B gave rise to the expected derivative **3k** as a white oil (287 mg, 59%).

Rf (CH_2Cl_2/AcOEt: 1/1) 0.16. 1H NMR (300 MHz, $CDCl_3$) δ 10.08 (s, 1H, CHO), 8.32 (s, 1H, H-triazole), 8.19 – 8.12 (m, 1H, H-Ar), 8.09 (d, J = 6.7 Hz, 3H, H-Ar), 7.87 (d, J = 7.7 Hz, 1H, H-Ar), 7.63 (dt, J = 7.7, 4.2 Hz, 2H, H-Ar), 7.49 (t, J = 7.6 Hz, 2H, H-Ar), 6.27 (d, J = 4.8 Hz, 1H, H-1'), 6.03 (dt, J = 9.5, 5.3 Hz, 2H, H-2', H-3'), 5.52 (qd, J = 6.7, 4.0 Hz, 1H, H-5'), 4.77 (t, J = 4.0 Hz, 1H, H-4'), 4.15 – 4.01 (m, 4H, O-C<u>H</u>$_2$-

CH_3), 2.39 – 2.10 (m, 2H, H-6', H-6''), 2.06, 2.04 (2s, 6H, Ac), 1.28 (td, J = 7.1, 3.3 Hz, 6H, O-CH_2-C<u>H</u>$_3$). 13C NMR (75 MHz, $CDCl_3$) δ 192.5 (C=O,), 170.2, 169.8 (C=O, Ac), 165.5 (C=O, Bz), 147.5 (C=CH), 137.5, 134.4, 132.1, 131.6, 130.3, 130.3, 129.9, 129.3, 129.2, 127.6 (C-Ar), 120.4 (C=CH), 90.4 (C-1'), 85.0 (d, J = 9.7 Hz, C-4'), 74.8 (C-2'), 71.2 (C-3'), 68.2 (C-5'), 62.9, 62.7 (2d, J = 6.8 Hz, O-<u>C</u>H$_2$-CH_3), 27.9 (d, J = 141.7 Hz, C-6'), 21.5, 20.9 (CH_3, Ac), 16.9 (d, J = 6.1 Hz, O-CH_2-<u>C</u>H$_3$). 31P NMR (81 MHz, $CDCl_3$) δ 25.18. MS ESI-QTof>0, m/z 644.20 (M+H) +, 666.18 (M+Na) +. HR-MS Calculated for $C_{30}H_{35}N_3O_{11}P$: 644.2009 (M+H) +; found: 644.2010.

6-Deoxy-6-diethylphosphono-1-(4-(3-formylphenyl)-1*H*-1,2,3-triazol-1-yl)-β-D-allofuranose (4k)

Tetramethylguanidine was added to **3k** (266 mg, 0.41 mmol) was dissolved in mixture of CH_2Cl_2 and MeOH (1:1), stirred at room temperature for 1 h. The reaction was quenched by dowex H+, then filtered and concentrated. The obtained product was purified by column chromatography to give **4k** as a white solid (165 mg, 88%). Rf (CH_2Cl_2/MeOH: 9/1) 0.47. 1H NMR (300 MHz, DMSO) δ 10.10 (d, J = 5.5 Hz, 1H, H-aldehyde), 8.92 (s, 1H, H-triazole), 8.40 (s, 1H, H-Ar), 8.20 (d, J = 7.7 Hz, 1H, H-Ar), 7.91 (d, J = 7.6 Hz, 1H, H-Ar), 7.72 (t, J = 7.7 Hz, 1H, H-Ar), 5.97 (d, J = 5.6 Hz, 1H, H-1'), 5.62 (d, J = 6.4 Hz, 1H, OH), 5.53 (d, J = 5.8 Hz, 1H, OH), 5.28 (d, J = 5.1 Hz, 1H, OH), 4.47 (dd, J = 11.3, 5.7 Hz, 1H, H-2'), 4.27 (dd, J = 8.1, 5.0 Hz, 1H, H-3'), 4.16 – 3.84 (m, 6H, H-4', H-5', O-C<u>H</u>$_2$-CH_3), 2.14 – 1.74 (m, 2H, H-6', H-6''), 1.21 (td, J = 7.0, 3.5 Hz, 6H, O-CH_2-C<u>H</u>$_3$). 13C NMR (75 MHz, DMSO) δ 193.1 (C=O, aldehyde), 145.5 (C=CH), 136.8, 131.5, 130.9, 129.9, 129.3, 125.6 (C-Ar), 120.6 (C=CH), 91.9 (C-1'), 88.5 (d, J = 15.9 Hz, C-4'), 75.3 (C-2'), 69.4 (C-3'), 65.6 (d, J = 4.0 Hz, C-5'), 61.0, 60.7 (2d, J = 6.2 Hz, O-<u>C</u>H$_2$-CH_3), 29.4 (d, J = 138.9 Hz, C-6'),

16.2 (d, J = 6.0 Hz, O-CH_2-$\underline{C}H_3$). 31P NMR (81 MHz, DMSO) δ 29.55. MS ESI-QTof>0, m/z 456.15 (M+H) +, 910.30 (2M +H) +. HR-MS Calculated for $C_{19}H_{27}N_3O_8P$: 456.1536 (M +H) +; found: 456.1535.

6-Deoxy-6-phosphono-1-(4-(3-formylphenyl)-1*H*-1,2,3-triazol-1-yl)-β-D-allofuranose disodium salt (1k)

Treatment of compound **4k** (127 mg, 0.28 mmol) following general procedure E gave rise to the expected derivative **1k** as white solid (85.3 mg, 69%).

Rf (iPrOH/NH_4OH/H_2O: 7/2/1) 0.06. 1H NMR (400 MHz, D_2O) δ 9.78 (s, 1H, H-aldehyde), 8.42 (s, 1H, H-triazole), 7.93 (t, J = 1.4 Hz, 1H, H-Ar), 7.89 – 7.82 (m, 1H, H-Ar), 7.74 (dd, J = 6.5, 1.3 Hz, 1H, H-Ar), 7.52 (t, J = 7.7 Hz, 1H, H-Ar), 6.11 (d, J = 5.1 Hz, 1H, H-1'), 4.69 (t, J = 5.2 Hz, 1H, H-2'), 4.63 – 4.49 (m, 1H, H-3'), 4.25 (ddd, J = 13.7, 8.4, 4.0 Hz, 2H, H-4', H-5'), 2.16 – 1.65 (m, 2H, H-6', H-6''). 13C NMR (100 MHz, D_2O) δ 195.7 (C=O), 146.3 (C=CH), 136.0, 131.8, 130.0, 129.9, 129.8, 126.6 (C-Ar), 120.9 (C=CH), 91.8 (C-1'), 88.4 (d, J = 13.7 Hz, C-4'), 75.3 (C-2'), 69.4 (C-3'), 67.4 (C-5'), 31.5 (d, J = 130.7 Hz, C-6'). 31P NMR (121 MHz, D_2O) δ 20.36. MS ESI-QTof>0, m/z 400.09 (M-2Na+3H) +, 422.07 (M-Na+H) +, 444.06 (M+H) +. MS ESI-QTof <0, m/z 398.07 (M-2Na+H)-. HR-MS Calculated for $C_{15}H_{17}N_3O_8Na_2P$: 444.0549 (M+H) +; found: 444.0553. UV (H_2O) λmax = 235 nm (εmax = 34028).

3-*O*-Benzoyl-6-deoxy-2,5-di-*O*-acetyl-6-diethylphosphono-1-(4-(4-formylphenyl)-1*H*-1,2,3-triazol-1-yl)-β-D-allofuranose (3l)

Treatment of compound **2** (347 mg, 0.67 mmol) following general procedure B gave rise to the expected derivative **3l** as a brown oil (245 mg, 57%).

Rf (CH_2Cl_2/AcOEt: 1/1) 0.15. 1H NMR (300 MHz, $CDCl_3$) δ 8.12-8.07 (m, 3H, H-triazole, H-Ar), 8.05 – 8.00 (m, 2H, H-Ar), 7.98 – 7.93 (m, 2H, H-Ar), 7.67 – 7.60 (m, 1H, H-Ar), 7.49 (dd, J = 10.5, 4.7 Hz, 2H, H-Ar), 6.28 (d, J = 4.7 Hz, 1H, H-1'), 6.02

(dt, J = 9.4, 5.4 Hz, 2H, H-2', H-3'), 5.63 – 5.44 (m, 1H, H-5'), 4.78 (t, J = 3.9 Hz, 1H, H-4'), 4.19 – 3.99 (m, 4H, O-C<u>H</u>$_2$-CH$_3$), 2.39 – 2.09 (m, 2H, H-6', H-6''), 2.05, 2.04 (2s, 6H, Ac), 1.40 – 1.17 (m, 6H, O-CH$_2$-C<u>H</u>$_3$). 13C NMR (75 MHz, CDCl$_3$) δ 192.31 (C=O, aldehyde), 170.3, 169.6 (C=O, Ac), 165.3 (C=O, Bz), 147.2 (C=CH), 136.7, 136.1, 134.4, 131.0, 130.4, 129.3, 126.8 (C-Ar), 120.8 (C=CH), 90.4 (C-1'), 85.1 (d, J = 12.1 Hz, C-4'), 74.9 (C-2'), 71.2 (C-3'), 68.2 (C-5'), 62.8, 62.6 (2d, J = 6.8 Hz, O-<u>C</u>H$_2$-CH$_3$), 27.9 (d, J = 137.9 Hz, C-6'), 21.5, 20.8 (CH$_3$, Ac), 16.9 (d, J = 6.1 Hz, O-CH$_2$-<u>C</u>H$_3$). 31P NMR (81 MHz, CDCl$_3$) δ 25.11. MS ESI-QTof>0, m/z 644,20 (M+H) +, 666.18 (M+Na) +, 671.16 (M+Na+5H) +. HR-MS Calculated for $C_{30}H_{35}N_3O_{11}P$: 644.2009 (M+H) +; found: 644.2012.

6-Deoxy-6-diethylphosphono-1-(4-formylphenyl-1*H*-1,2,3-triazol-1-yl)-β-D-allo-furanose (4l)

Treatment of compound **3l** (205 mg, 0.32 mmol) following procedure described for **4k** gave rise to the expected derivative **4l** as a white oil (77 mg, 45%).

Rf (CH$_2$Cl$_2$/MeOH: 9/1) 0.22. 1H NMR (400 MHz, DMSO) δ 10.04 (s, 1H, H-aldehyde), 8.96 (s, 1H, H-triazole), 8.12 (d, J = 8.2 Hz, 2H, H-Ar), 8.02 (d, J = 8.3 Hz, 2H, H-Ar), 5.98 (d, J = 5.6 Hz, 1H, H-1'), 5.64 (d, J = 6.2 Hz, 1H, OH), 5.55 (d, J = 5.9 Hz, 1H, OH), 5.30 (d, J = 4.9 Hz, 1H, OH), 4.47 (dd, J = 10.7, 5.4 Hz, 1H, H-2'), 4.30 – 4.23 (m, 1H, H-3'), 4.07 – 3.92 (m, 6H, H-4', H-5', O-C<u>H</u>$_2$-CH$_3$), 2.08 – 1.79 (m, 2H, H-6', H-6''), 1.22 (td, J = 7.0, 4.2 Hz, 6H, O-CH$_2$-C<u>H</u>$_3$). 13C NMR (100 MHz, DMSO) δ 192.5 (C=O, aldehyde), 145.5 (C=CH), 136.3, 135.7, 130.3, 125.9 (C-Ar), 121.6 (C=CH), 91.8 (C-1'), 88.8 (d, J = 15.9 Hz, C-4'), 75.6 (C-2'), 69.6 (C-3'), 65.8 (d, J = 4.1 Hz, C-5'), 61.2, 60.9 (2d, J = 6.1 Hz, O-<u>C</u>H$_2$-CH$_3$), 29.6 (d, J = 138.8 Hz, C-6'), 16.4 (d, J = 6.1 Hz, O-CH$_2$-<u>C</u>H$_3$). 31P NMR (121 MHz, DMSO) δ 29.36. MS ESI-

QTof>0, m/z 456.15 (M+H) +, 478.13 (M+Na) +, 911.30 (2M+H) +. HR-MS Calculated for $C_{19}H_{27}N_3O_8P$: 456.1536 (M+H) +; found: 456.1532.

6-Deoxy-6-phosphono-1-(4-formylphenyl-1*H*-1,2,3-triazol-1-yl)-β-D-allofuranose disodium salt (1l)

Treatment of compound **4l** (66 mg, 0.15 mmol) following general procedure E gave rise to the expected derivative **1l** as a white solid (54 mg, 83%).

Rf (iPrOH/NH_4OH/H_2O: 7/2/1) 0.07. 1H NMR (400 MHz, D_2O) δ 9.79 (s, 1H, H-aldehyde), 8.53 (s, 1H, H-triazole), 7.80 (d, J = 8.5 Hz, 2H, H-Ar), 7.75 (d, J = 8.4 Hz, 2H, H-Ar), 6.10 (d, J = 5.0 Hz, 1H, H-1’), 4.67 (t, J = 5.1 Hz, 1H, H-2’), 4.61 – 4.51 (m, 1H, H-3’), 4.33 – 4.12 (m, 2H, H-4’, H-5’), 2.02 – 1.67 (m, 2H, H-6’, H-6’’). 13C NMR (100 MHz, D_2O) δ 195.6 (C=O, aldehyde), 146.4 (C=CH), 135.4, 135.2, 130.7, 125.9 (C-Ar), 121.8 (C=CH), 91.8 (C-1’), 88.4 (d, J = 13.8 Hz, C-4’), 75.3 (C-2’), 69.3 (C-3’), 67.4 (C-5’), 31.5 (d, J = 130.1 Hz, C-6’). 31P NMR (121 MHz, D_2O) δ 20.04. MS ESI-QTof>0, m/z 400.09 (M-2Na+3H) +, 415.21 (M-CHO) +, 438.20 (M-CHO+Na) +. MS ESI-QTof <0, m/z 398.07 (M-2Na+H)-. HR-MS Calculated for $C_{15}H_{17}N_3O_8P$: 398.0753 (M-Na+H)-; found: 398.0742. UV (H_2O) λmax = 287 nm (εmax = 23473).

3-*O*-Benzoyl-6-deoxy-2,5-di-*O*-acetyl-6-diethylphosphono-1-(4-benzoyl-1*H*-1,2,3-triazol-1-yl)-β-D-allofuranose 3m

Treatment of compound **2** (353 mg, 0.69 mmol) following general procedure A gave rise to the expected derivative **3m** as a white oil (414 mg, 93%).

Rf (CH_2Cl_2/AcOEt: 1/1) 0.27. 1H NMR (300 MHz, $CDCl_3$) δ 8.46 – 8.39 (m, 3H, H-triazole, H-Ar), 8.12 – 8.07 (m, 2H, H-Ar), 7.63 (ddd, J = 8.9, 5.1, 1.3 Hz, 2H, H-Ar), 7.56 – 7.44 (m, 4H, H-Ar), 6.29 (d, J = 4.8 Hz, 1H, H-1’), 6.02 (dt, J = 9.5, 5.4 Hz, 2H, H-2’, H3’), 5.56 – 5.44 (m, 1H, H-5’), 4.84 (t, J = 3.9 Hz, 1H, H-4’), 4.16 – 4.03 (m,

4H, O-C$\underline{H}_2$-CH_3), 2.38 – 2.15 (m, 2H, H-6’, H-6’’), 2.11, 2.03 (2s, 6H, Ac), 1.28 (ddd, J = 8.5, 6.0, 2.4 Hz, 6H, O-CH_2-C$\underline{H}_3$). 13C NMR (75 MHz, $CDCl_3$) δ 185.4 (C=O, ketone), 169.8, 169.3 (C=O, Ac), 164.6 (C=O, Bz), 148.3 (C=CH), 136.4, 134.0, 133.6, 130.7, 129.9, 128.8, 128.6 (C-Ar), 128.5 (C=CH), 89.9 (C-1’), 84.4 (d, J = 8.5 Hz, C-4’), 74.3 (C-2’), 70.4 (C-3’), 67.6 (C-5’), 62.2, 62.0 (2d, J = 6.2 Hz, O-$\underline{C}H_2$-CH_3), 27.3 (d, J = 141.3 Hz, C-6’), 20.9, 20.3 (CH_3, Ac), 16.3 (d, J = 6.1 Hz, O-CH_2-$\underline{C}H_3$). 31P NMR (81 MHz, $CDCl_3$) δ 24.88. MS ESI-QTof>0, m/z 666.18 (M+Na) +, 667.19 (M+Na+H) +. HR-MS Calculated for $C_{30}H_{34}N_3O_{11}NaP$: 666.1829 (M+Na) +; found: 666.1833.

6-Deoxy-6-diethylphosphono-1-(4-benzoyl-1*H*-1,2,3-triazol-1-yl)-β-D-allofuran-ose (4m)

Treatment of compound **3m** (375 mg, 0.58 mmol) following general procedure D gave rise to the expected derivative **4m** as white oil (225 mg, 85%).

Rf (CH_2Cl_2/MeOH: 9/1) 0.43. 1H NMR (300 MHz, DMSO) δ 9.12 (s, 1H, H-triazole), 8.29 – 8.12 (m, 2H, H-Ar), 7.71 (t, J = 7.4 Hz, 1H, H-Ar), 7.60 (t, J = 7.5 Hz, 2H, H-Ar), 6.05 (d, J = 5.3 Hz, 1H, H-1’), 5.68 (d, J = 5.8 Hz, 1H, OH), 5.53 (d, J = 5.6 Hz, 1H, OH), 5.30 (d, J = 5.2 Hz, 1H, OH), 4.48 (dd, J = 10.4, 5.2 Hz, 1H, H-2’), 4.28 (dd, J = 8.2, 4.8 Hz, 1H, H-3’), 4.08 – 3.88 (m, 6H, H-4’, H-5’, O-C$\underline{H}_2$-CH_3), 2.17 – 1.68 (m, 2H, H-6’, H-6’’), 1.21 (t, J = 7.0 Hz, 6H, O-CH_2-C$\underline{H}_3$). 13C NMR (75 MHz, DMSO) δ 185.4 (C=O), 146.9 (C=CH), 136.8, 133.7, 130.2 (C-Ar), 129.0 (C=CH), 128.9 (C-Ar), 92.4 (C-1’), 88.7 (d, J = 14.9 Hz, C-4’), 75.6 (C-2’), 69.5 (C-3’), 65.8 (d, J = 3.8 Hz, C-5’), 61.3, 61.0 (2d, J = 6.2 Hz, O-$\underline{C}H_2$-CH_3), 29.7 (d, J = 138.7 Hz, C-6’), 16.5 (d, J = 6.0 Hz, O-CH_2-$\underline{C}H_3$). 31P NMR (81 MHz, DMSO) δ 29.35. MS ESI-QTof>0, m/z 415.21 (M-2Na+6H) +, 456.15 (M +H) +, 478.14 (M+Na) +, 911.30 (2M+H) +. HR-MS Calculated for $C_{19}H_{27}N_3O_8P$: 456.1536 (M +H) +; found: 456.1538.

6-Deoxy-6-phosphono-1-(4-benzoyl-1*H*-1,2,3-triazol-1-yl)-β-D-allofuranose disodium salt (1m)

Treatment of compound **4m** (184 mg, 0.40 mmol) following general procedure E gave rise to the expected derivative **1m** as a white solid (167 mg, 94%).

Rf (iPrOH/NH_4OH/H_2O: 7/2/1) 0.12. 1H NMR (400 MHz, D_2O) δ 8.84 (s, 1H, H-triazole), 8.04 – 7.88 (m, 2H, H-Ar), 7.79 – 7.66 (m, 1H, H-Ar), 7.58 (t, J = 7.8 Hz, 2H, H-Ar), 6.21 (d, J = 4.5 Hz, 1H, H-1'), 4.75 (t, J = 4.9 Hz, 1H, H-2'), 4.59 (t, J = 4.9 Hz, 1H, H-3'), 4.36 – 4.27 (m, 1H, H-4'), 4.22 (ddd, J = 13.4, 8.6, 4.5 Hz, 1H, H-5'), 1.87 (dddd, J = 23.8, 16.6, 15.0, 6.7 Hz, 2H, H-6', H-6''). 13C NMR (100 MHz, D_2O) δ 188.7 (C=O), 145.7 (C=CH), 136.2, 134.3, 129.8 (C-Ar), 129.4 (C=CH), 128.9 (C-Ar), 92.1 (C-1'), 88.3 (d, J = 13.9 Hz, C-4'), 75.3 (C-2'), 69.2 (C-3'), 67.1 (d, J = 3.0 Hz, C-5'), 31.4 (d, J = 130.6 Hz, C-6'). 31P NMR (121 MHz, D_2O) δ 20.29. MS ESI-QTof>0, m/z 400.09 (M-2Na+3H) +, 422.07 (M-Na+H) +, 444.05 (M+H) +, 494.18 (M+2Na+5H) +. MS ESI-QTof <0, m/z 398.07 (M-2Na+H)-. HR-MS Calculated for $C_{15}H_{17}N_3O_8Na_2P$: 444.0549 (M+H) +; found: 444.0546. UV (H2O) λmax = 259 nm (εmax = 15548).

3-*O*-Benzoyl-6-deoxy-2,5 -di-*O*-acetyl-6-diethylphosphono-1-(4-acetyl-1H-1,2,3-triazol-1-yl)-β-D-allofuranose (3n)

Treatment of compound **2** (458 mg, 0.89 mmol) following general procedure A gave rise to the expected derivative **3n** as a white oil (442 mg, 85%).

Rf: (CH_2Cl_2/AcOEt: 1/1) 0.17. 1H NMR (300 MHz, $CDCl_3$) δ 8.22 (s, 1H, H-triazole), 8.08 (dd, J = 8.4, 1.3 Hz, 2H, H-Ar), 7.69 – 7.58 (m, 1H, H-Ar), 7.50 (dd, J = 10.8, 4.4 Hz, 2H, H-Ar), 6.22 (d, J = 4.7 Hz, 1H, H-1'), 6.05 – 5.91 (m, 2H, H-2', H-3'), 5.48 (dd, J = 11.7, 3.7 Hz, 1H, H-5'), 4.81 (t, J = 3.8 Hz, 1H, H-4'), 4.24 – 3.82 (m, 4H, O-C$\underline{H}_2$-CH_3), 2.70 (s, 3H, CO-CH_3), 2.39 – 2.13 (m, 2H, H-6', H-6''), 2.11 (s, 3H, Ac), 2.02 (s,

3H, Ac), 1.28 (ddd, J = 8.5, 6.1, 2.5 Hz, 6H, O-CH_2-$C\underline{H}_3$). 13C NMR (75 MHz, $CDCl_3$) δ 192.9 (C=O, CO-CH_3), 170.2, 169.7 (C=O, Ac), 165.4 (C=O, Bz), 148.7 (C=C), 134.4, 130.3, 129.3, 129.2 (C-Ar), 125.9 (C=CH), 90.5 (C-1'), 85.0 (d, J = 8.8 Hz, C-4'), 74.8 (C-3'), 71.0 (C-2'), 68.2 (C-5'), 62.8, 62.6 (2d, J = 6.2 Hz, O-$\underline{C}H_2$-CH_3), 27.9 (d, J = 141.3 Hz, C-6'), 21.5, 20.8 (CH_3, Ac), 16.9 (d, J = 6.1 Hz, O-CH_2-$\underline{C}H_3$). 31P NMR (81 MHz, $CDCl_3$) δ 24.87. MS ESI-QTof>0, m/z 582.19 (M+H) +, 583.19 (M+2H) +, 604.17 (M+Na) +. HR-MS Calculated for $C_{25}H_{33}N_3O_{11}P$: 582.1853 (M+H) +; found: 582.1854.

6-Deoxy-6-diethylphosphono-1-(4-acetyl-1*H*-1,2,3-triazol-1-yl)-β-D-allofuranose (4n)

Treatment of compound **3n** (442 mg, 0.72 mmol) following general procedure D gave rise to the expected derivative **4n** as a white oil (223 mg, 78%).

Rf (CH_2Cl_2/MeOH: 9/1) 0.35. 1H NMR (300 MHz, DMSO) δ 8.96 (s, 1H, H-triazole), 5.98 (d, J = 5.5 Hz, 1H, H-1'), 5.63 (d, J = 6.2 Hz, 1H, OH), 5.51 (d, J = 5.7 Hz, 1H, OH), 5.27 (d, J = 5.2 Hz, 1H, OH), 4.41 (dd, J = 11.0, 5.5 Hz, 1H, H-2'), 4.24 (dd, J = 8.2, 5.0 Hz, 1H, H-3'), 4.06 – 3.87 (m, 6H, H-4', H-5', O-$C\underline{H}_2$-CH_3), 2.57 (s, 3H, CO-CH_3), 2.11 – 1.77 (m, 2H, H-6', H-6''), 1.22 (t, J = 7.0 Hz, 6H, O-CH_2-$C\underline{H}_3$). 13C NMR (75 MHz, DMSO) δ 191.6 (C=O), 147.3 (C=C), 126.1 (C=CH), 91.7 (C-1'), 88.5 (d, J = 11.4 Hz, C-4'), 75.1 (C-2'), 69.2 (C-3'), 65.5 (d, J = 3.8 Hz, C-5'), 61.0, 60.7 (2d, J = 6.2 Hz, O-$\underline{C}H_2$-CH_3), 29.5 (d, J = 144.1 Hz, C-6'), 27.2 (CO-CH_3), 16.2 (d, J = 6.0 Hz, O-CH_2-$\underline{C}H_3$). 31P NMR (81 MHz, DMSO) δ 29.15. MS ESI-QTof>0, m/z 394.1380 (M+H) +, 395.1407 (M+2H) +, 416.12 (M+Na) +, 787.27(2M+H) +. HR-MS Calculated for $C_{14}H_{25}N_3O_8P$: 394.1379 (M+H) +; found: 394.1380.

6-Deoxy-6-phosphono-1-(4-acetyl-1*H*-1,2,3-triazol-1-yl)-β-D-allofuranose disodium salt (1n)

Treatment of compound **4n** (187 mg, 0.48 mmol) following general procedure E gave rise to the expected derivative **1n** as a white solid (68 mg, 38%).

Rf (iPrOH/NH_4OH/H_2O: 7/2/1) 0.08. 1H NMR (400 MHz, D_2O) δ 8.84 (s, 1H, H-triazole), 6.19 (d, J = 4.7 Hz, 1H, H-1'), 4.74 (t, J = 4.9 Hz, 1H, H-2'), 4.57 (t, J = 4.8 Hz, 1H, H-3'), 4.26 (t, J = 4.0 Hz, 1H, H-4'), 4.23 – 4.15 (m, 1H, H-5'), 2.66 (s, 3H, CO-CH_3), 1.98 – 1.74 (m, 2H, H-6', H-6''). 13C NMR (100 MHz, D_2O) δ 195.2 (C=O), 146.7 (C=C), 127.6 (C=CH), 92.0 (C-1'), 88.5 (d, J = 14.0 Hz, C-4'), 75.2 (C-2'), 69.4 (C-3'), 67.2 (d, J = 3.1 Hz, C-5'), 31.4 (d, J = 130.9 Hz, C-6'), 27.1 (CO-CH_3). 31P NMR (121 MHz, D_2O) δ 20.77. MS ESI-QTof>0, m/z 454.04 (M+3Na+4H) +. MS ESI-QTof <0, m/z 336.06 (M-2Na+H)-. HR-MS Calculated for $C_{10}H1_5N_3O_8P$: 336.0597 (M-2Na+H)-; found: 336.0598. UV (H_2O) λmax = 232 nm (εmax = 9706).

3-*O*-Benzoyl-6-deoxy-2,5-di-*O*-acetyl-6-diethylphosphono-1-(4-methoxycarbonyl-1*H*-1,2,3-triazol-1-yl)-β-D-allofuranose (3o)

Treatment of compound **2** (138 mg, 0.27 mmol) following general procedure A gave rise to the expected derivative **3o** (157 mg, 98%).

Rf (CH_2C_{l2}/MeOH, 9/1, v/v) 0.50. 1H NMR (400 MHz, $CDCl_3$) δ = 8.25 (s, 1H, CH triazole), 8.10-8.05 (m, 2H, H-Ar Bz), 7.64-7.49 (m, 3H, H-Ar Bz), 6.25 (d, J = 4.44 Hz, 1H, H-1'), 6.12-5.95 (m, 2H, H-2' H-3'), 5.61-5.46 (m, 1H, H-4'), 4.81 (t, J = 3.51 Hz, 1H H-5'), 4.17-4.0 (m, 4H, O-CH_2-CH_3), 3.97 (s, 3H, O-CH_3), 2.36-2.18 (m, 2H, H-6', H-6''), 2.10, 2.02 (2s, 6H, Ac), 1.28 (td, J = 2.55 and 7.11 Hz, 6H, O-CH_2-CH_3). 13C NMR (100 MHz, DMSO) δ = 169.7, 169.2 (C=O Ac), 164.9 (C=O, Bz), 140.3 (C=CH), 133.9, 129.8, 128.7 (C-Ar), 127.4 (C=CH), 90.0 (C-1'), 84.7 (d, J = 8.8 Hz, C-4'), 76.4 (C-2'), 74.3 (C-3'), 70.4 (C-5'), 67.7, 67.4 (2d, J = 6.4 Hz, O-CH_2-CH_3), 28.4 (d, J = 141.2 Hz, C-6'), 21.0, 20.3 (CH_3, Ac), 16.4 (m, J = 6.1 Hz, O-CH_2-CH_3).

31P NMR (121 MHz, $CDCl_3$) δ = 29.8. MS ESI-QTof >0, m/z 598.18 (M+H) +. HR-MS Calculated for $C_{25}H_{33}N_3O_{12}P$: 598.1807; found: 598.1802.

6-Deoxy-6-diethylphosphono-1-(4-methoxycarbonyl-1*H*-1,2,3-triazol-1-yl)-β-D-allofuranose (4o)

Sodium methoxide was added to **3o** (365 mg, 0.61 mmol) dissolved in dry methanol at room temperature and stirred for 1 h, then reaction mixture was concentrated under vacuum. Purification of the crude material on silica gel chromatography gave rise to the expected derivative **4o** as a white solid (226 mg, 90%).

Rf (CH_2Cl_2/MeOH: 9/1) 0.28. 1H NMR (300 MHz, DMSO) δ 9.01 (s, 1H, H-triazole), 5.99 (d, J = 5.5 Hz, 1H, H-1'), 5.65 (d, J = 5.9 Hz, 1H, OH), 5.52 (d, J = 5.6 Hz, 1H, OH), 5.30 (d, J = 4.6 Hz, 1H, OH), 4.43 (d, J = 5.0 Hz, 1H, H-2'), 4.25 (d, J = 3.1 Hz, 1H, H-3'), 4.03 – 3.90 (m, 6H, H-4', H-5', O-C$\underline{H}_2$-CH_3), 3.86 (s, 3H, $COOCH_3$), 2.12 – 1.73 (m, 2H, H-6', H-6''), 1.22 (t, J = 7.0 Hz, 6H, O-CH_2-C$\underline{H}_3$). 13C NMR (75 MHz, DMSO) δ 160.8 (C=O, ester), 139.1 (C=CH, 128.1 (C=CH), 92.25 (C-1'), 88.7 (d, J = 15.0 Hz, C-4'), 75.4 (C-2'), 69.4 (C-3'), 65.7 (d, J = 3.8 Hz, C-5'), 61.3, 61.0 (2d, J = 6.1 Hz, O-$\underline{C}H_2$-CH_3), 52.1 ($COOCH_3$), 29.6 (d, J = 138.6 Hz, C-6'), 16.4 (d, J = 5.9 Hz, O-CH_2-$\underline{C}H_3$). 31P NMR (81 MHz, DMSO) δ 29.33. MS ESI-QTof>0, m/z 410.12 (M+H)+, 448.08 (M+Na-7H)+, 819.23 (2M+H)+. HR-MS Calculated for $C_{14}H_{20}N_4O_7P$: 410.1328 (M+H) +; found: 410.1330.

6-Deoxy-6-phosphono-1-(4-methylester-1*H*-1,2,3-triazol-1-yl)-β-D-allofuranose disodium salt (1o)

Treatment of compound **4o** (185 mg, 0.45 mmol) following general procedure E gave rise to the expected derivative **1o** as a white solid (119 mg, 67%).

Rf (iPrOH/NH_4OH/H_2O: 7/2/1) 0.12. 1H NMR (400 MHz, D_2O) δ 8.76 (s, 1H, H-triazole), 6.16 (d, J = 4.6 Hz, 1H, H-1'), 4.72 (t, J = 4.9 Hz, 1H, H-2'), 4.55 (t, J = 4.7

Hz, 1H, H-3’), 4.21 (t, J = 4.1 Hz, 1H, H-4’), 4.15 (ddd, J = 17.9, 8.8, 4.6 Hz, 1H, H-5’), 3.93 (s, 3H, $COOCH_3$), 2.02 – 1.77 (m, 2H, H-6’, H-6’’). 13C NMR (100 MHz, D_2O) δ 162.3 (C=O, ester), 139.4 (C=C), 128.3 (C=CH), 92.0 (C-1’), 88.3 (d, J = 14.7 Hz, C-4’), 75.1 (C-2’), 69.5 (C-3’), 66.8 (d, J = 2.9 Hz, C-5’), 52.7 ($COOCH_3$), 31.1 (d, J = 133.5 Hz, C-6’). 31P NMR (121 MHz, D_2O) δ 22.27. MS ESI-QTof>0, m/z 354.07 (M-2Na+3H) +, 376.05 (M-Na+2H) +, 422.87 (M+Na+2H) +, 707.13 (2M-4Na+5H) +. MS ESI-QTof <0, m/z 352.05 (M-2Na+H)-. HR-MS Calculated for $C_{10}H_{15}N_3O_9P$: 352.0546 (M-2Na+H)-; found: 352.0542. UV (H2O) λmax = 211 nm (εmax = 11461).

6-Deoxy-6-phosphono-1-(4-sodiumcarboxylate-1*H*-1,2,3-triazol-1-yl)-β-D-allofuranose disodium salt (1p)

Compound **1o** (62 mg, 0.16 mmol) was dissolved in water, then sodium hydroxide solution 0.1 M was added and stirred at room temperature for 2 days. Then, the reaction was quenched by adding hydrochloric acid 0.1 N and concentrated under reduced pressure. The obtained residue was purified on reserve phase (gradient: water to methanol 100%), which was passed through a Dowex Na+ ion exchange column. The desired fractions were collected and freeze dried leading to the expected derivative **1p** as a white solid (57 mg, 90%).

Rf (iPrOH/NH_4OH/H_2O: 7/2/1) 0.01. 1H NMR (400 MHz, D_2O) δ 8.46 (s, 1H, H-triazole), 6.14 (d, J = 4.9 Hz, 1H, H-1’), 4.72 (t, J = 5.1 Hz, 1H, H-2’), 4.56 (t, J = 5.1 Hz, 1H, H-3’), 4.23 (t, J = 3.9 Hz, 1H, H-4’), 4.20 – 4.12 (m, 1H, H-5’), 2.08 – 1.70 (m, 2H, H-6’, H-6’’). 13C NMR (100 MHz, D_2O) δ 167.3 (C=O, ester), 144.9 (C=CH), 126.2 (C=CH), 91.7 (C-1’), 88.3 (d, J = 13.9 Hz, C-4’), 75.0 (C-2’), 69.4 (C-3’), 67.0 (d, J = 2.3 Hz, C-5’), 31.3 (d, J = 132.2 Hz, C-6’). 31P NMR (121 MHz, D_2O) δ 21.03. MS ESI-QTof>0, m/z 318.05 (M-4Na+3H) +, 362.04 (M-2Na+3H) +, 384.02 (M-Na+2H) +, 406.00 (M+H) +, 701.08 (2M-5Na+6H) +. MS ESI-QTof <0, m/z 266.04

(M-6Na-6H)-, 294.05 (M-5Na-H)-, 339.04 (M-3Na-2H)-, 677.09 (2M-6Na+5H)-. HR-MS Calculated for $C_9H_{12}N_3O_9PNa_3$: 406.0004 (M+H)-; found: 406.0002. UV (H_2O) λmax = 266 nm (εmax = 2104).

6-Deoxy-6-diethylphosphono-1-(4-aminocarbonyl-1*H*-1,2,3-triazol-1-yl)-β-D-allofuranose (4q)

Treatment of compound **3o** (157.5 mg, 0.26 mmol) following general procedure D gave rise to the expected derivative **4q** (78 mg, 75%).

Rf (CH_2Cl_2/MeOH, 9/1, v/v) 0.18. 1H NMR (400 MHz, DMSO) δ = 8.76 (s, 1H, C-H triazole), 7.89, 7.51 (bs, 2H, NH_2), 5.96 (d, J = 5.6 Hz, 1H, H-1'), 5.61 (d, J = 6.3 Hz, 1H, OH), 5.49 (d, J = 5.7 Hz, 1H, OH), 5.25 (d, J = 5.1 Hz, 1H, OH), 4.41-4.36 (m, 1H, H-2'), 4.24-4.22 (m, 1H, H-3'), 4.02-3.69 (m, 6H, H-4', H-5', O-C$\underline{H}_2$-CH_3), 2.25-1.90 (m, 2H, H-6', H-6''), 1.22 (t, J = 7.1 Hz, 6H, O-CH_2-C$\underline{H}_3$). 13C NMR (100 MHz, DMSO) δ = 161.4 (C=O), 143.4 (C=CH), 125.3 (C=CH), 92.0 (C-1'), 88.6 (d, J = 14.3 Hz, C-4'), 75.9 (C-2'), 69.4 (C-3'), 65.7 (C-5'), 61.1 (dd, J = 5.9 and 26.4 Hz, O-$\underline{C}H_2$-CH_3), 30.3 (d, J = 138.3 Hz, C-6'), 16.4 (d, J = 5.9 Hz, O-CH_2-$\underline{C}H_3$). 31P NMR (121 MHz, $CDCl_3$) δ = 29.2. MS ESI-QTof >0, m/z 395.1 (M+H) +, 417.1 (M+Na) +, 789.3 (2M+H) +. HR-MS Calculated for $C_{13}H_{24}N_4O_8P$: 395.1332; found: 395.1334.

6-Deoxy-6-phosphono-1-(4-aminocarbonyl-1*H*-1,2,3-triazol-1-yl)-β-D-allofuranose disodium salt (1q)

Treatment of compound **4q** (68 mg, 0.20 mmol) following general procedure E gave rise to the expected derivative **1q** (60 mg, 90%).

Rf (iPrOH/NH_4OH/H_2O: 9/9/2) 0.52. 1H NMR (400 MHz, D_2O) δ = 8.74 (s, 1H, C-H triazole), 6.21 (d, J = 4.77 Hz, 1H, H-1'), 4.80-4.72 (m, 1H, H-2'), 4.59-4.57 (m, 1H, H-3'), 4.31-4.27 (m, 1H, H-4'), 4.24-4.05 (m, 1H, H-5'), 1.95-1.65 (m, 2H, H-6', H-6'').

13C NMR (100 MHz, D_2O) δ = 164.1 (C=O), 141.8 (C=CH), 126.3 (CH triazole), 91.8 (C-1’), 88.5 (d, J = 13.6 Hz, C-4’), 75.1 (C-2’), 69.2 (C-3’), 67.3 (d, J = 1.7 Hz, C-5’), 32.1 (d, J = 128.26 Hz, C-6’). 31P NMR (121 MHz, $CDCl_3$) δ = 19.2. MS ESI-QTof >0, m/z 361.05 (M-Na+2H) +, 383.03 (M+H) +. MS ESI-QTof <0, m/z 337.05 (M-2Na+H)-. HR-MS Calculated for $C_9H_{14}N_4O_8PNa_2$: 383.0343; found: 383.0345. UV (H_2O) λmax = 210 nm (εmax = 11900).

References

1. Gallier, F.; Lallemand, P.; Meurillon, M.; Jordheim, L. P.; Dumontet, C.; Périgaud, C.; Lionne, C.; Peyrottes, S.; Chaloin, L. *PLoS Comput Biol,* **2011**, *7* (12), e1002295.

2. Meurillon, M.; Marton, Z.; Hospital, A.; Jordheim, L. P.; Béjaud, J.; Lionne, C.; Dumontet, C.; Périgaud, C.; Chaloin, L.; Peyrottes, S. *European Journal of Medicinal Chemistry,* **2014**, *77* (0), 18-37.

3. Jones, G.; Willett, P.; Glen, R. C.; Leach, A. R.; Taylor, R. *Journal of Molecular Biology,* **1997**, *267* (3), 727-748.

4. Pedretti, A.; Villa, L.; Vistoli, G. *Journal of Computer-Aided Molecular Design,* **2004**, *18* (3), 167-173.

5. Gasteiger, J.; Marsili, M. *Tetrahedron*, **1980,** *36* (22), 3219-3228

6. Gallier, F.; Peyrottes, S.; Périgaud, C. *European Journal of Organic Chemistry*, **2007**, *6*, 925-933.
