# Supplementary material for: Beta-hydroxyphosphonate ribonucleoside analogues derived from 4-substituted-1,2,3-triazoles as IMP/GMP mimics: synthesis and biological evaluation
Source: Beilstein J Org Chem. 2016 Jul 18;12:1476–86. doi: 10.3762/bjoc.12.144 (PMC4979751; doi:10.3762/bjoc.12.144)

Supporting Information

for

Beta-hydroxyphosphonate ribonucleoside analogues derived from 4-substituted-1,2,3-triazoles as IMP/GMP mimics: synthesis and biological evaluation

Tai Nguyen Van[1], Audrey Hospital[1], Corinne Lionne[2], Lars P. Jordheim[3], Charles Dumontet[3], Christian Périgaud[1], Laurent Chaloin[2] and Suzanne Peyrottes[1,*]

Address: [1]Institut des Biomolécules Max Mousseron (IBMM), UMR 5247 CNRS – Université de Montpellier - ENSCM, Campus Triolet, cc1705, Place Eugène Bataillon, 34095 Montpellier, France [2]Centre d'études d'agents Pathogènes et Biotechnologies pour la Santé (CPBS), FRE 3689 CNRS - Université de Montpellier, 1919 route de Mende, 34293 Montpellier, France and [3]Université de Lyon, Université Claude Bernard Lyon 1, INSERM 1052, CNRS 5286, Centre Léon Bérard, Centre de Recherche en Cancérologie de Lyon, 69008 Lyon, France

Email: Peyrottes Suzanne - peyrottes@univ-montp2.fr

*Corresponding author

Spectral data for new compounds and 2D NMR studies

2D-NMR experiments for compounds **2**, **3a** and **3aa**	pages s2–s6
2D-NMR experiments for compounds **3h** and **3i**	pages s7–s10
1H NMR, 31P NMR for compound **1a**	s11
1H NMR, 31P NMR for compound **1b**	s12
1H NMR, 31P NMR for compound **1c**	s13
1H NMR, 31P NMR for compound **1d**	s14
1H NMR, 31P NMR for compound **1e**	s15
1H NMR, 31P NMR for compound **1f**	s16
1H NMR, 31P NMR for compound **1g**	s17
1H NMR, 31P NMR for compound **1h**	s18
1H NMR, 31P NMR for compound **1i**	s19
1H NMR, 31P NMR for compound **1j**	s20
1H NMR, 31P NMR for compound **1k**	s21
1H NMR, 31P NMR for compound **1l**	s22
1H NMR, 31P NMR for compound **1m**	s23
1H NMR, 31P NMR for compound **1n**	s24
1H NMR, 31P NMR for compound **1o**	s25
1H NMR, 31P NMR for compound **1p**	s26
1H NMR, 31P NMR for compound **1q**	s27

2D-NMR experiments for compound **2**

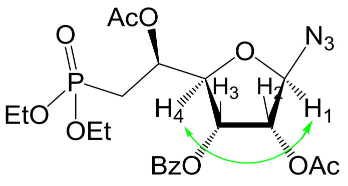

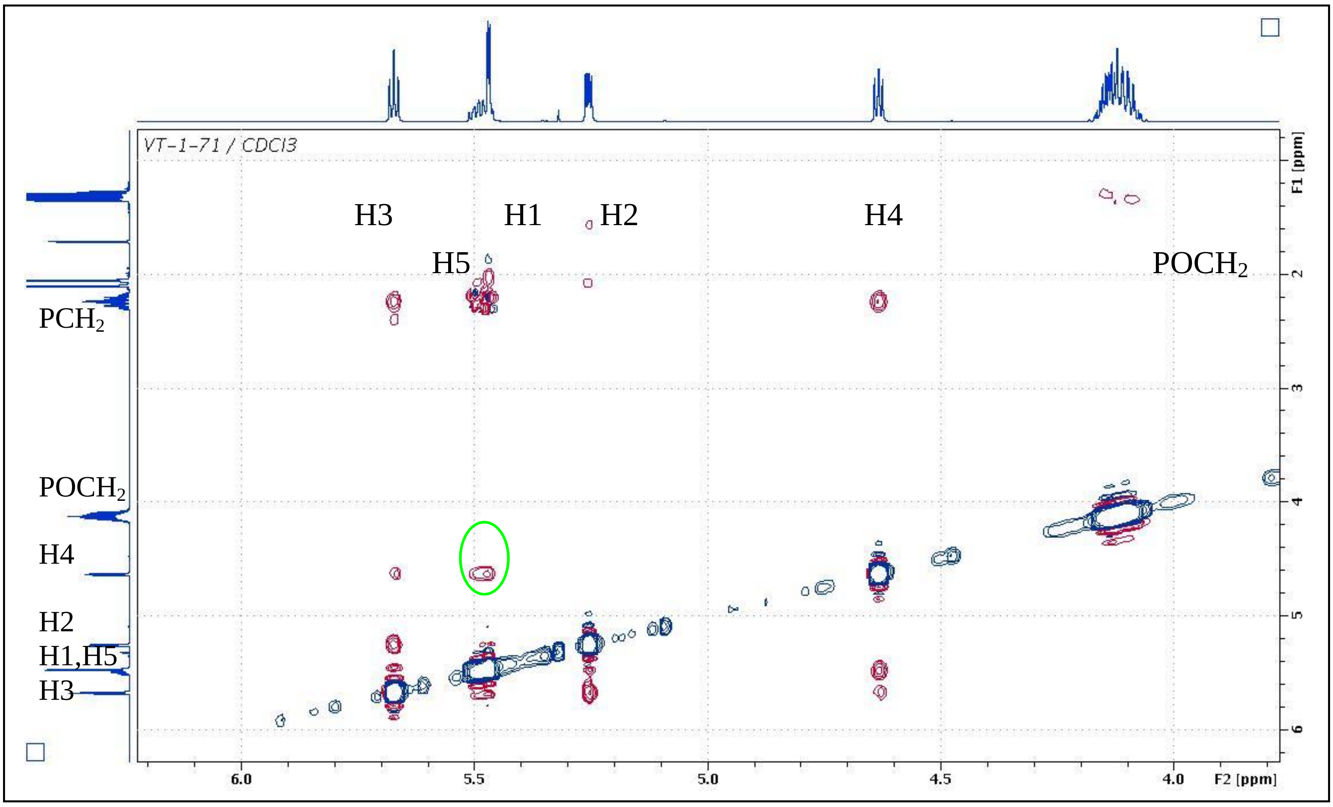

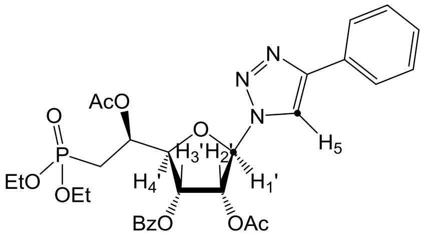

2D-NMR experiments for compounds **3a**

HSQC-experiment

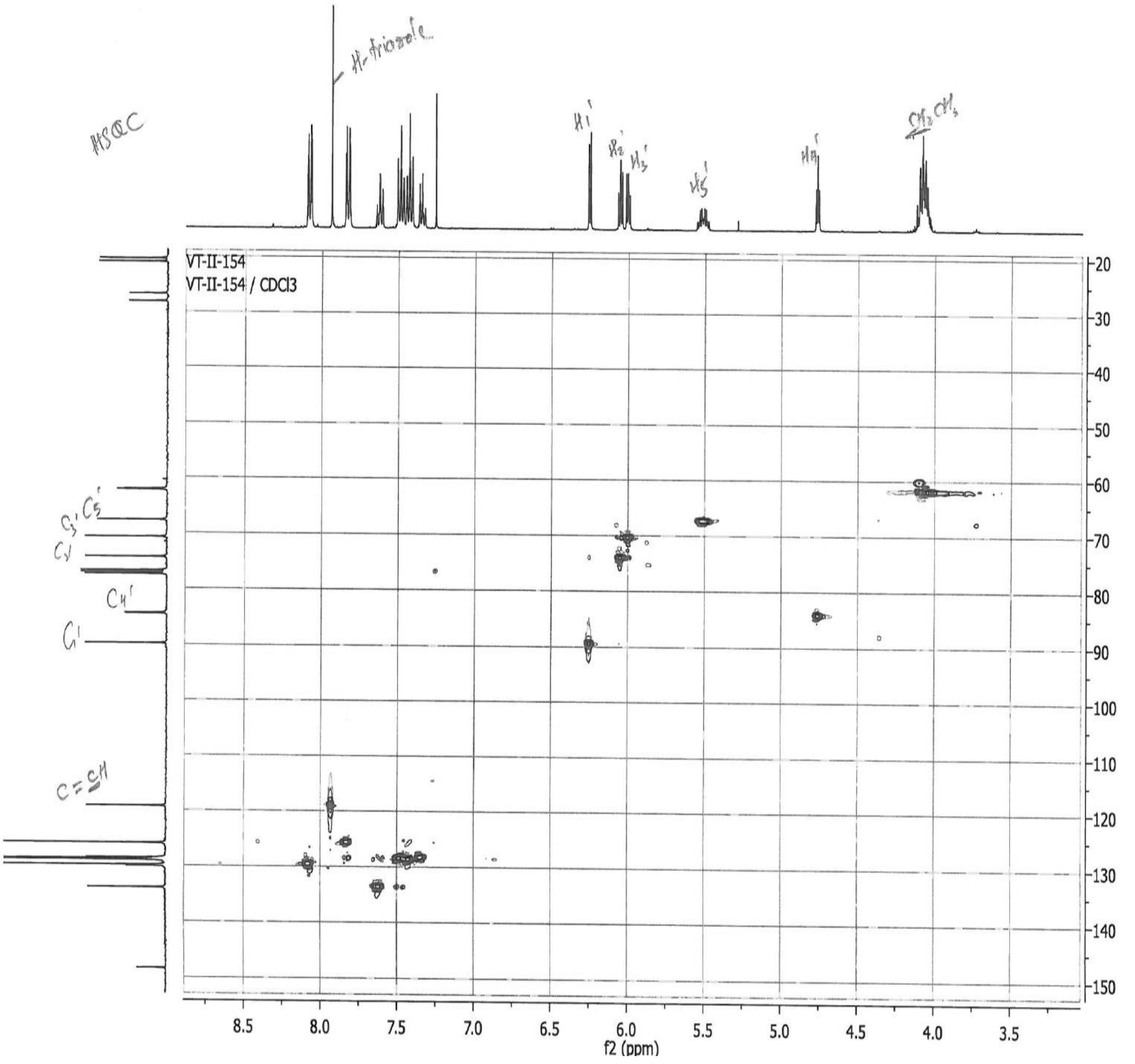

HMBC-experiment

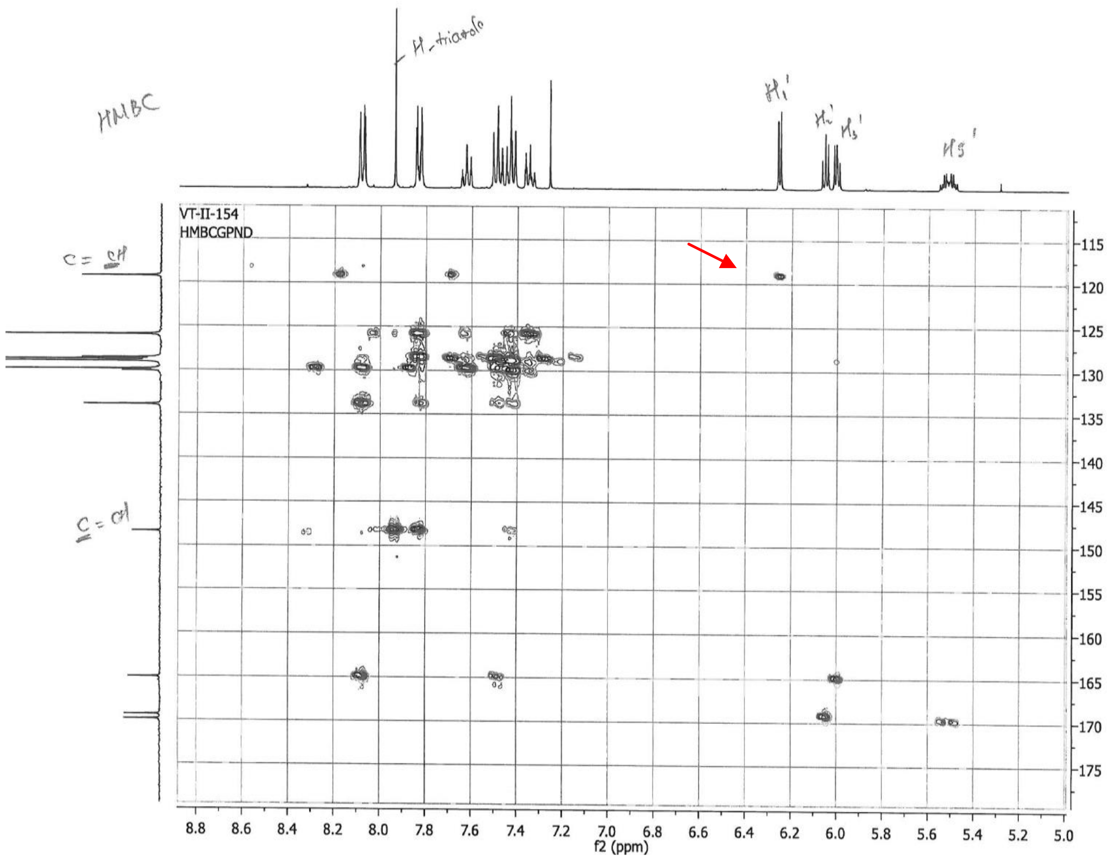

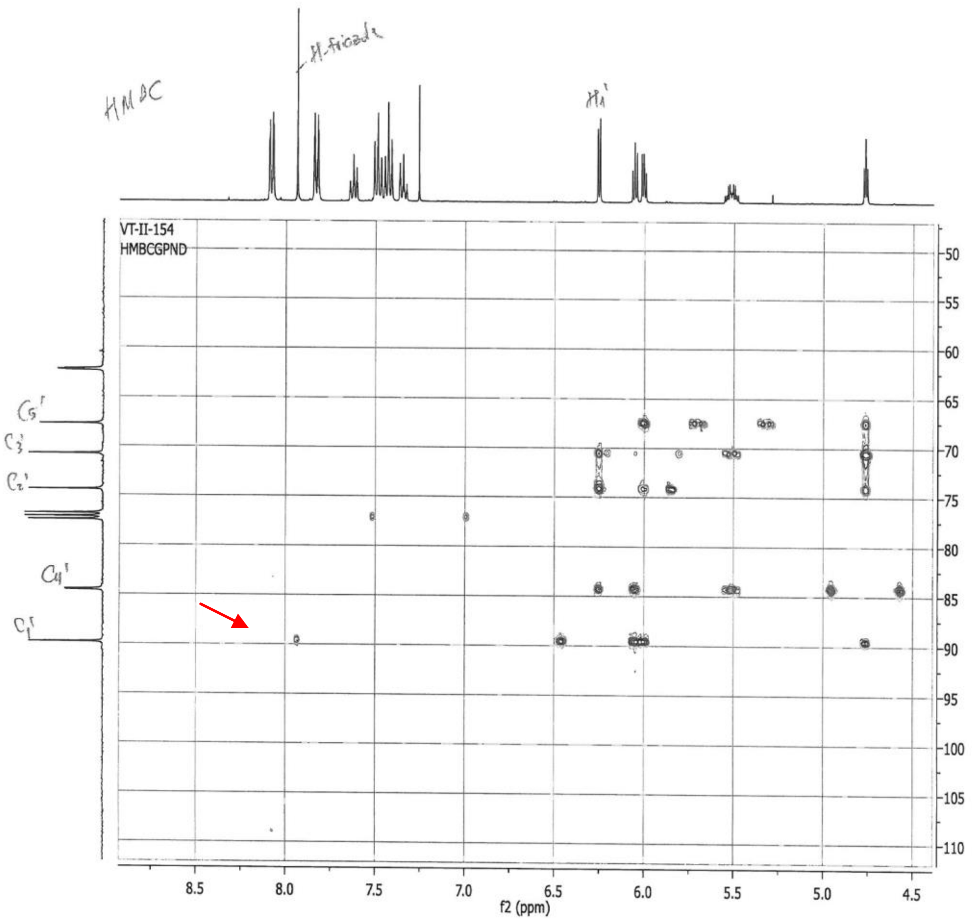

2D-NMR experiments for compound **3aa**

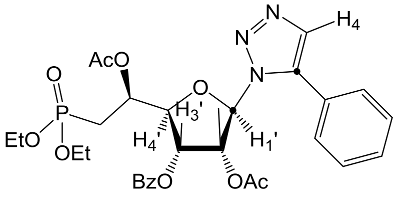

HSQC-experiment

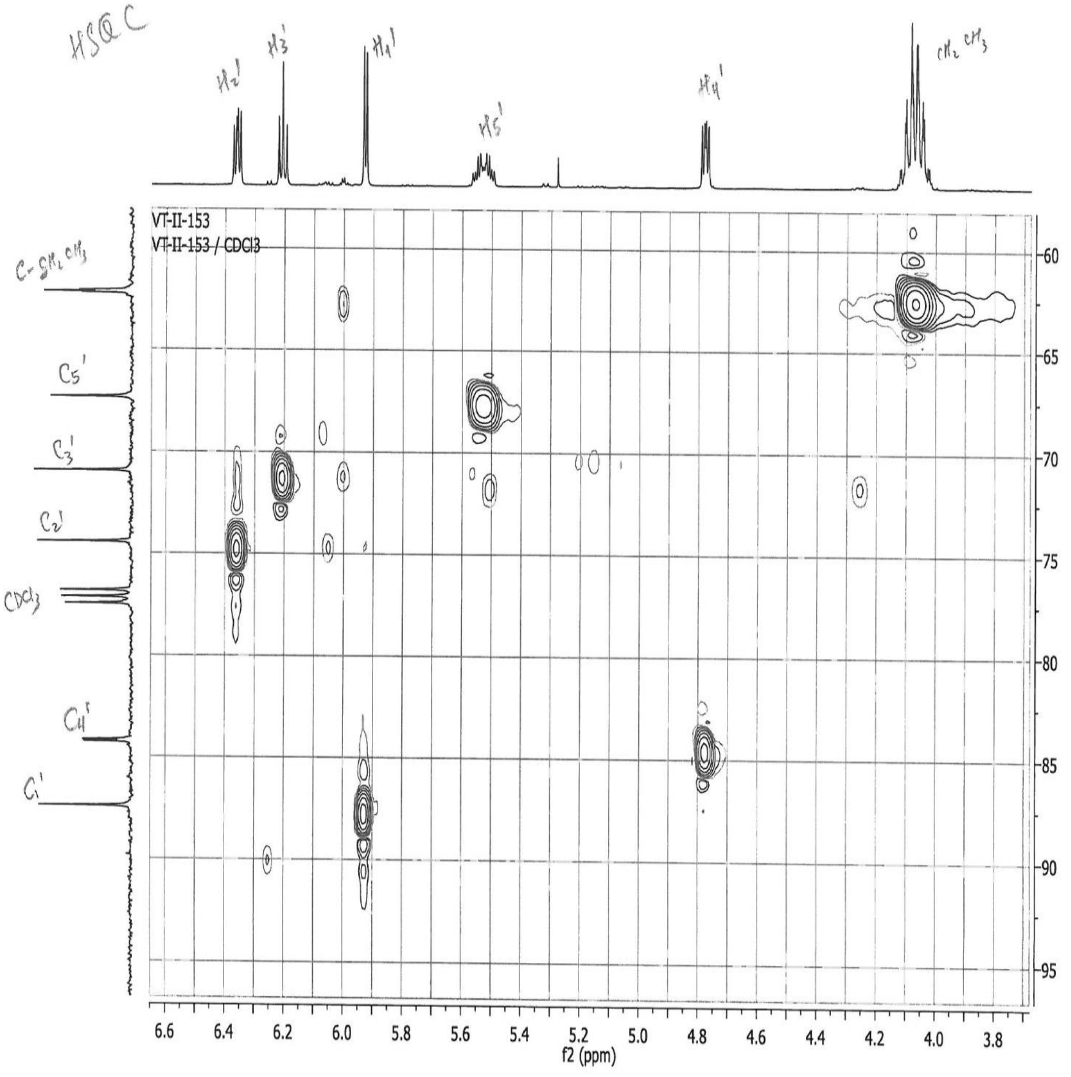

HMBC-experiment

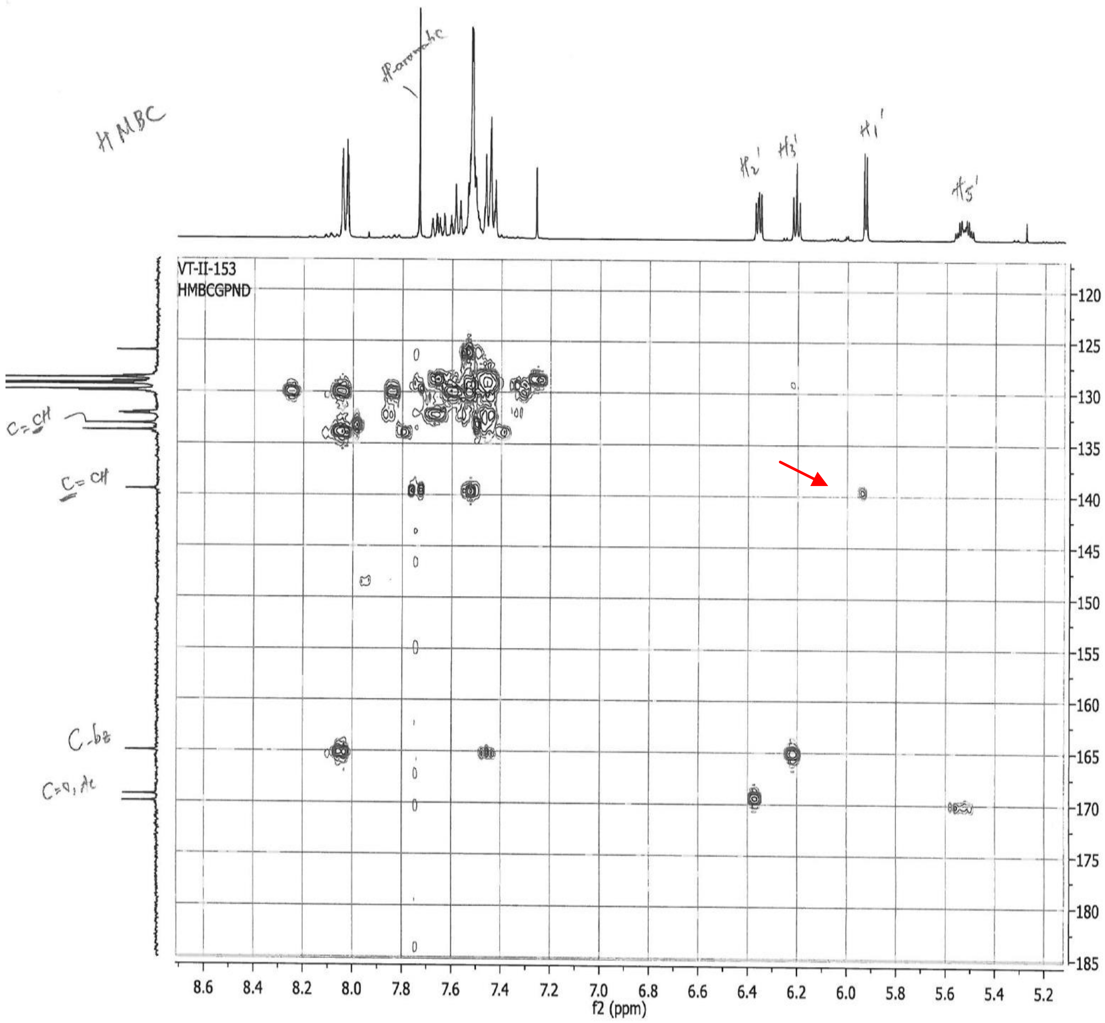

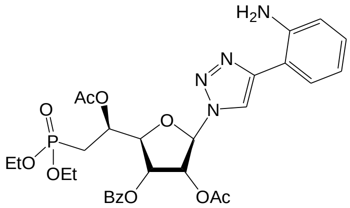

2D-NMR experiments for compounds **3h**

NOESY experiment

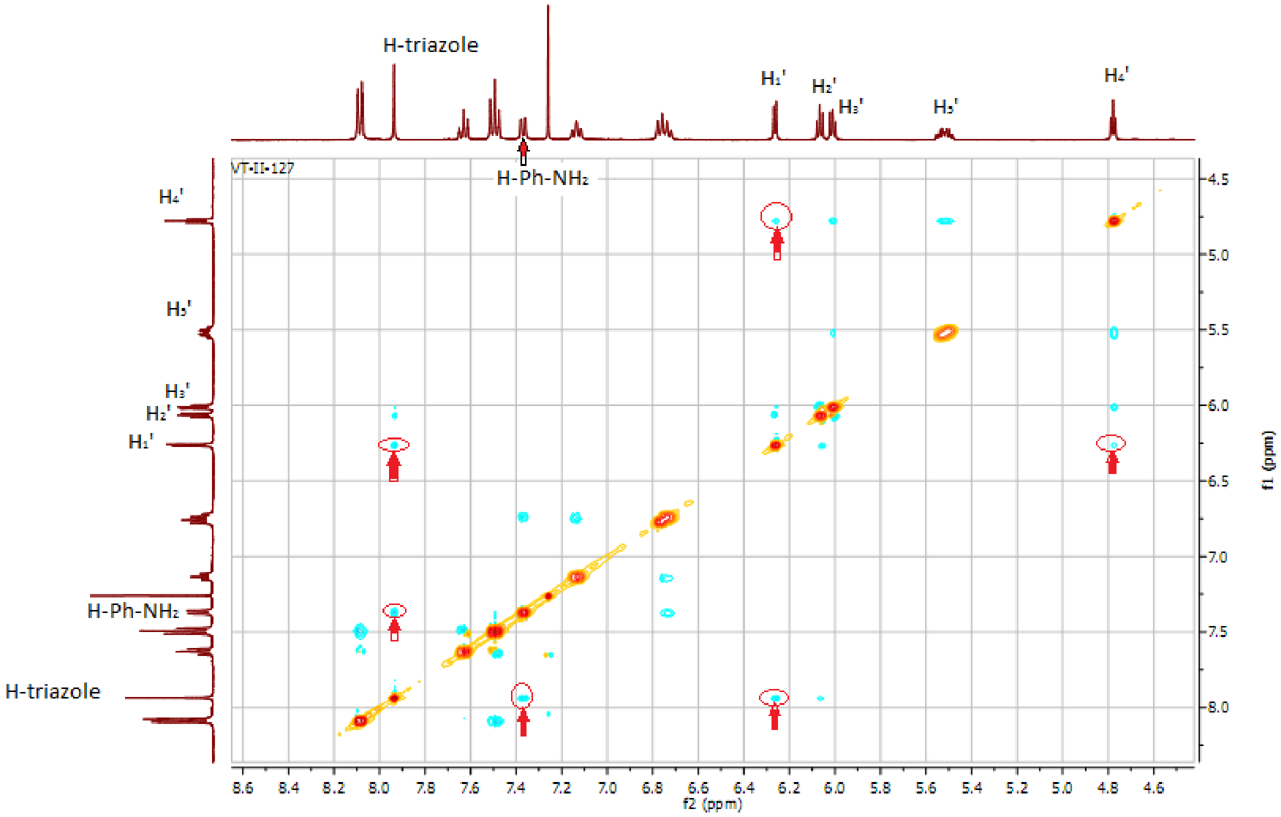

HMBC experiment

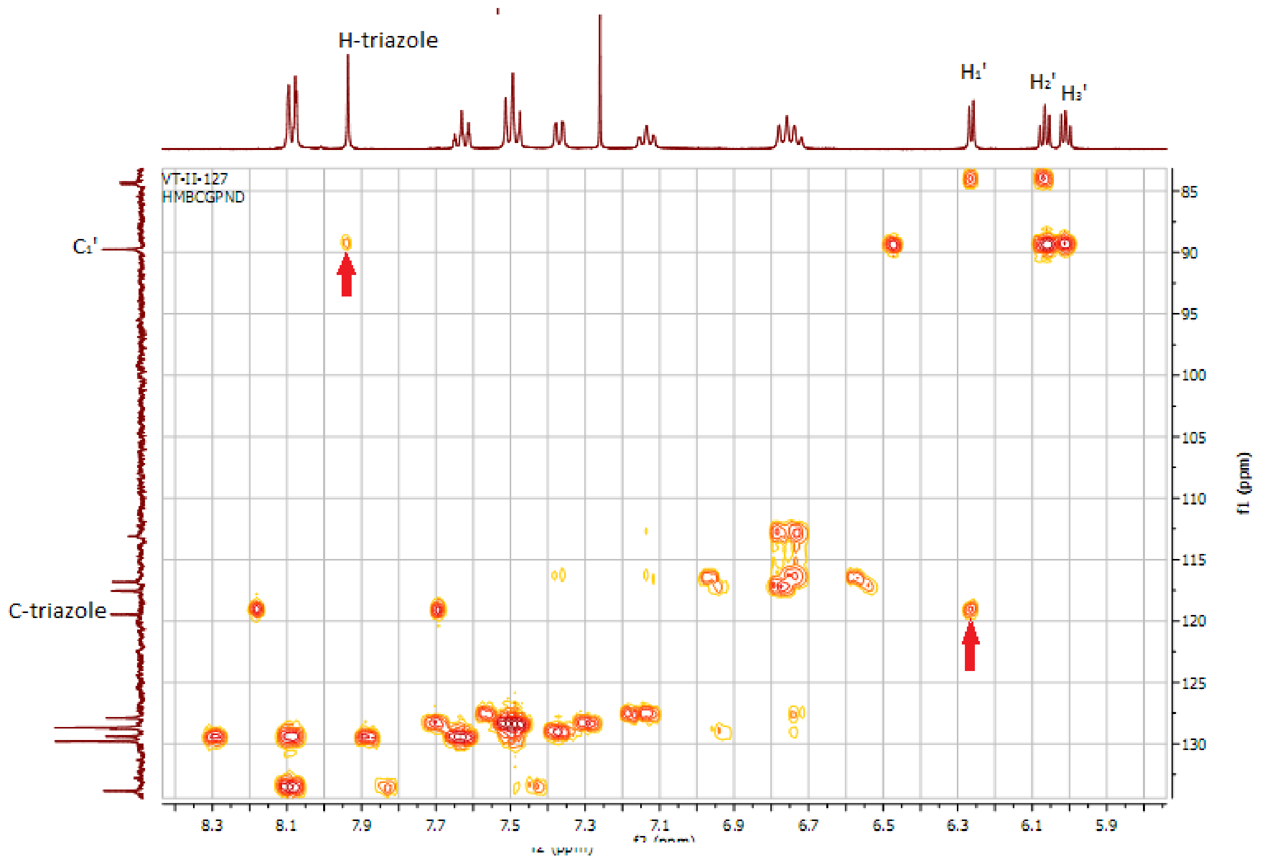

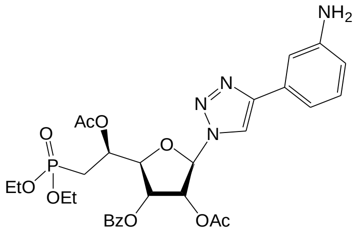

2D-NMR experiments for compounds **3i**

NOESY experiment

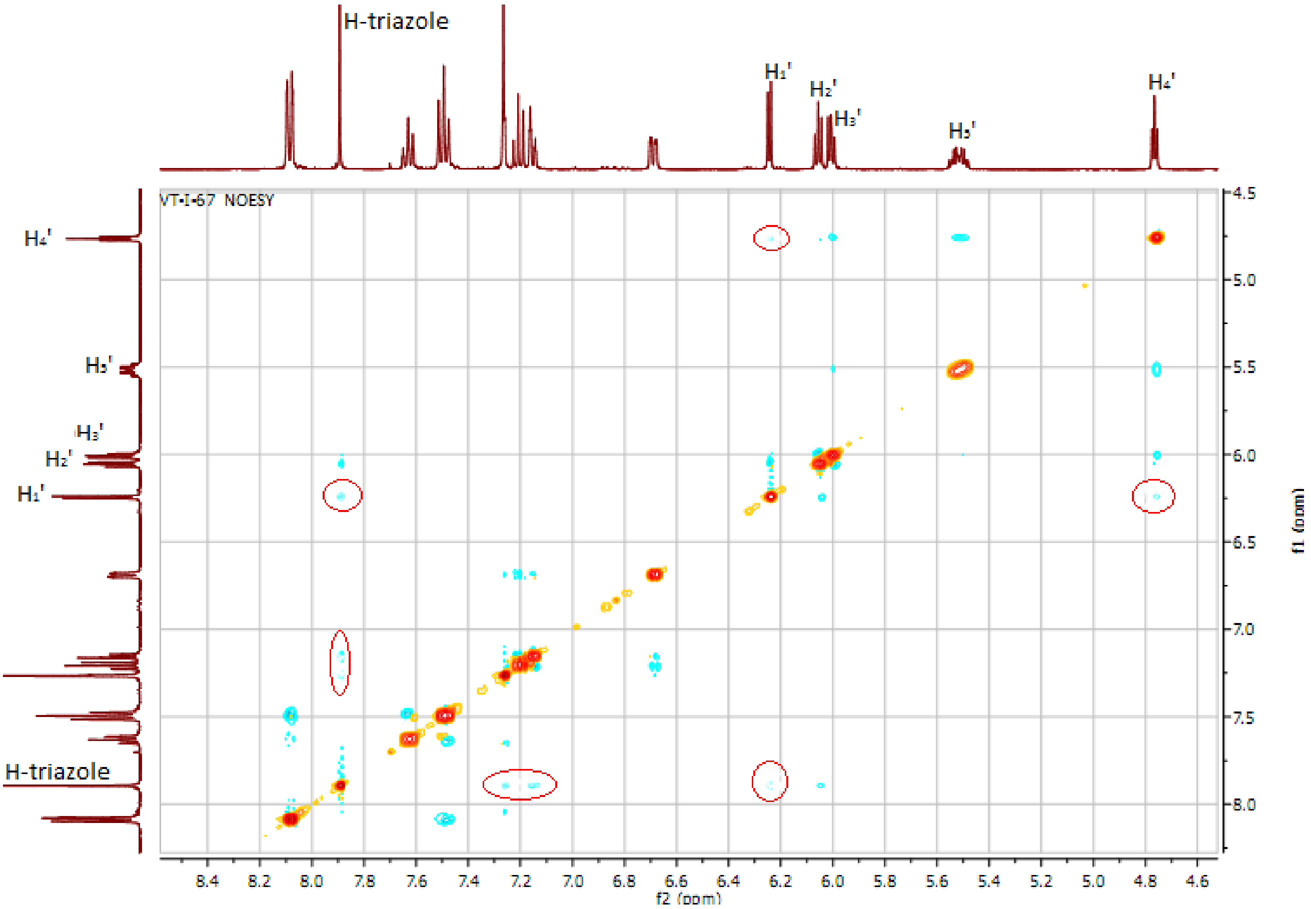

HMBC experiment

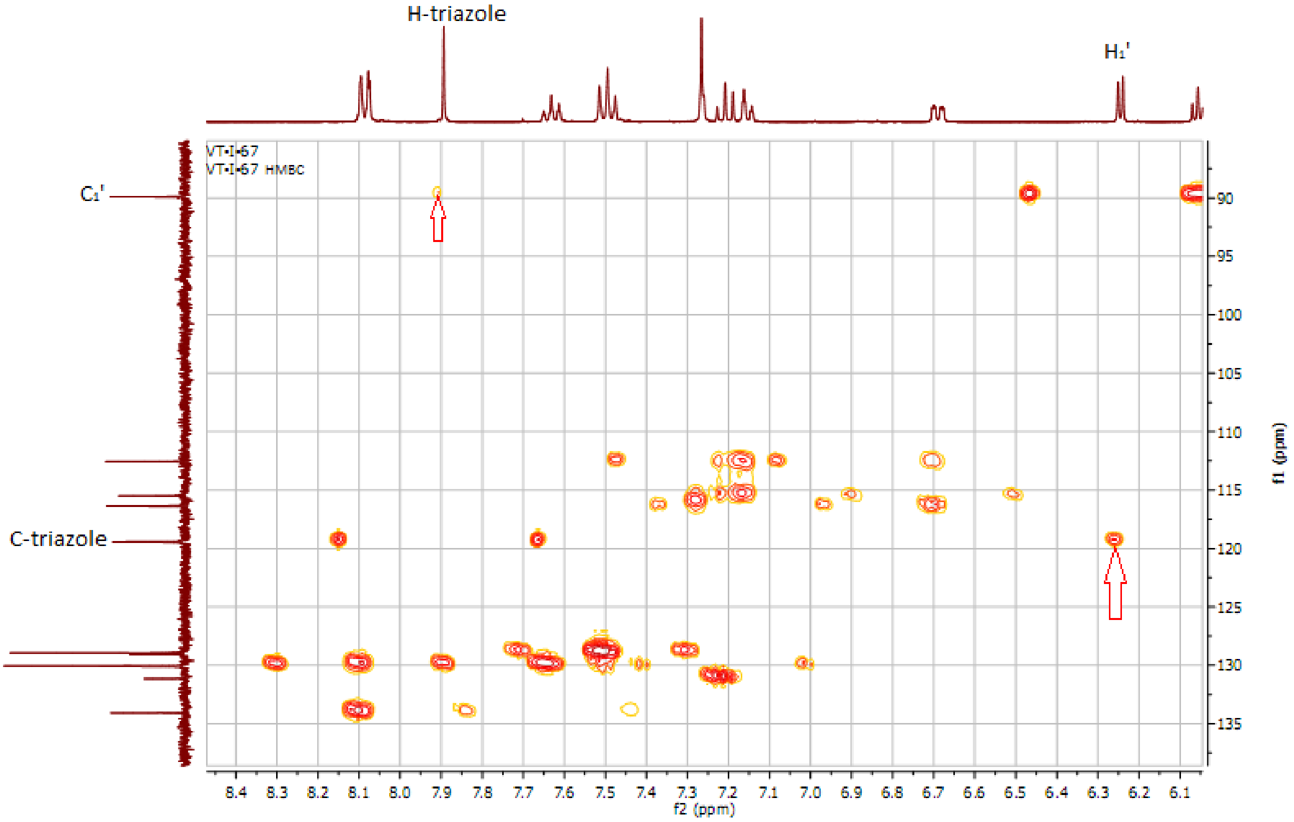

^{1}H NMR, ^{31}P NMR for Compound **1a**

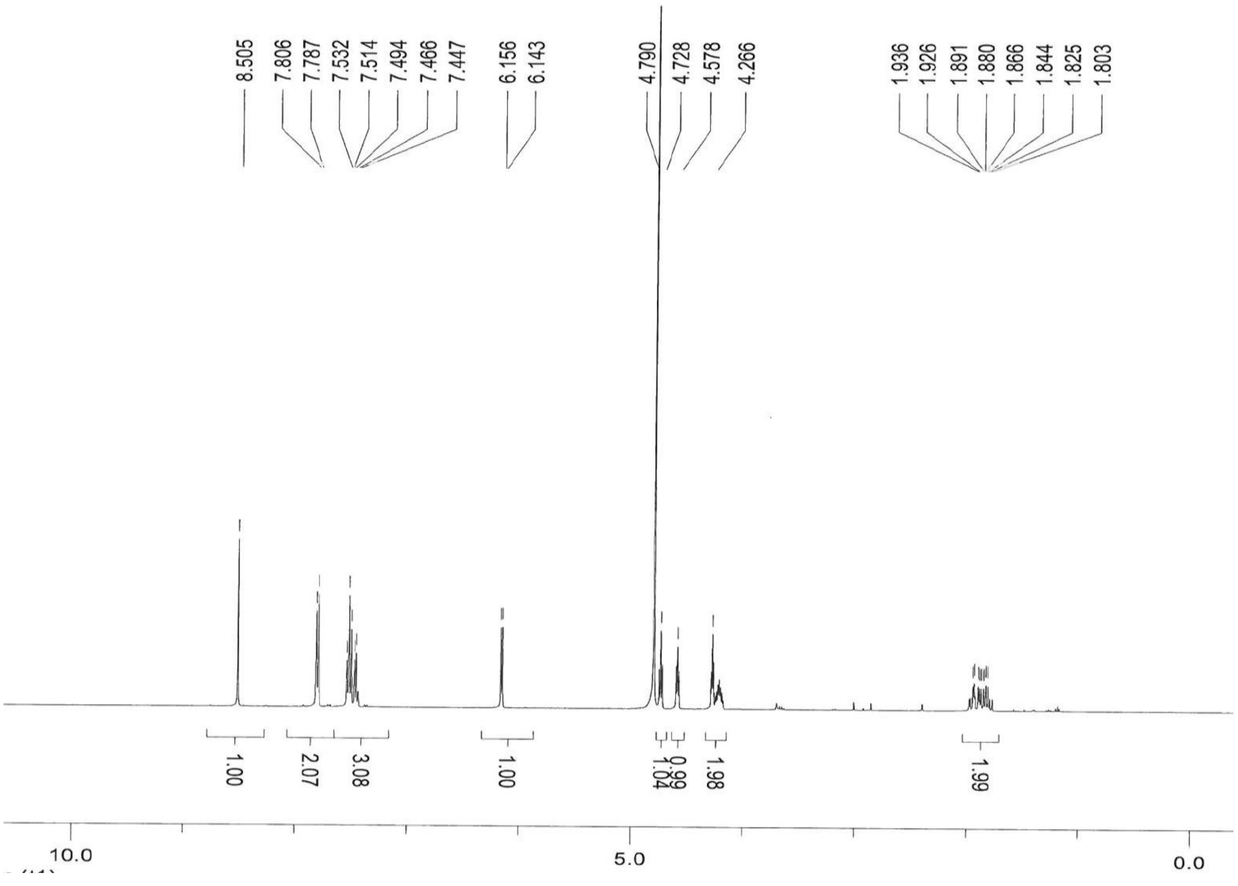

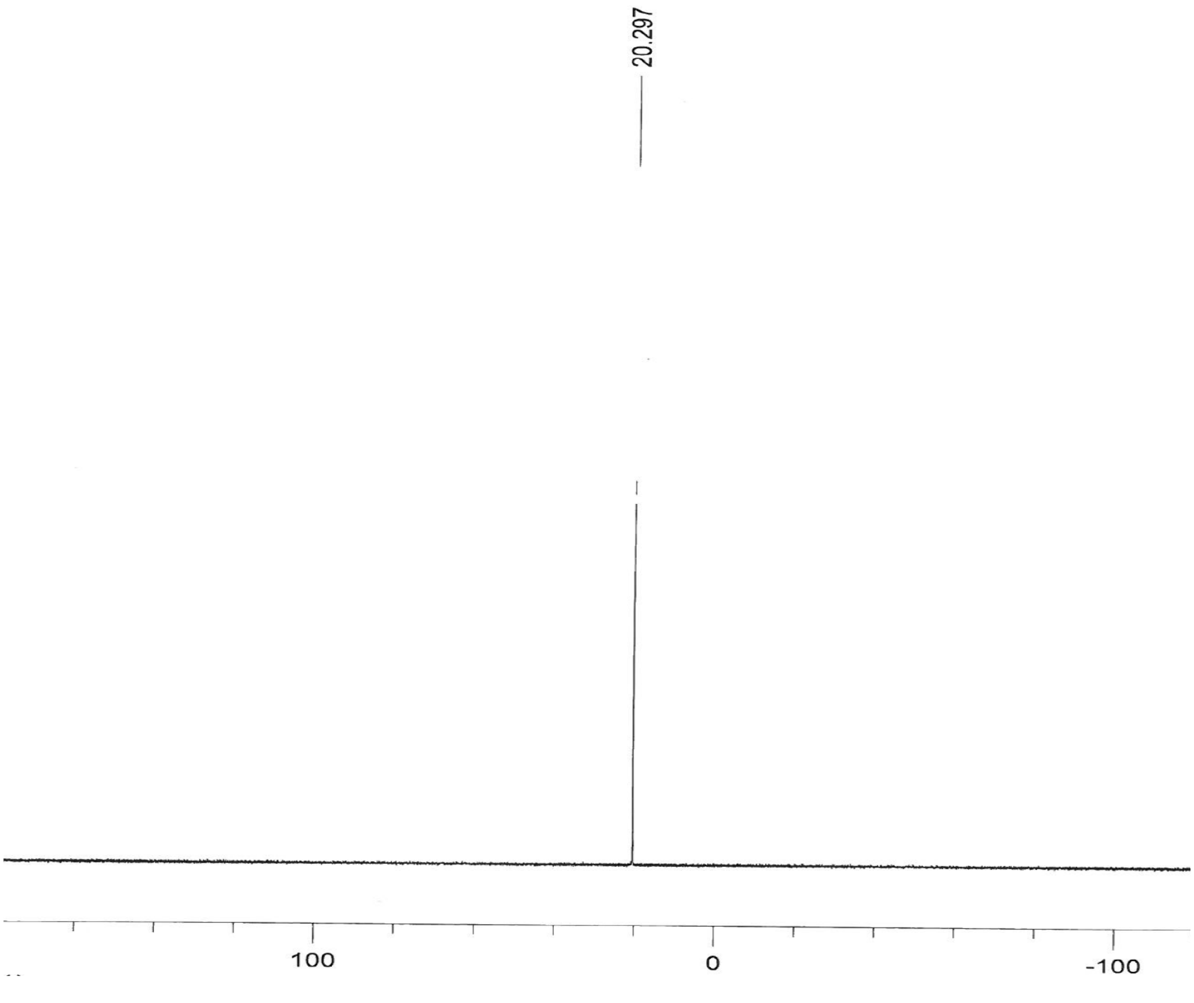

^{1}H NMR, ^{31}P NMR for compound **1b**

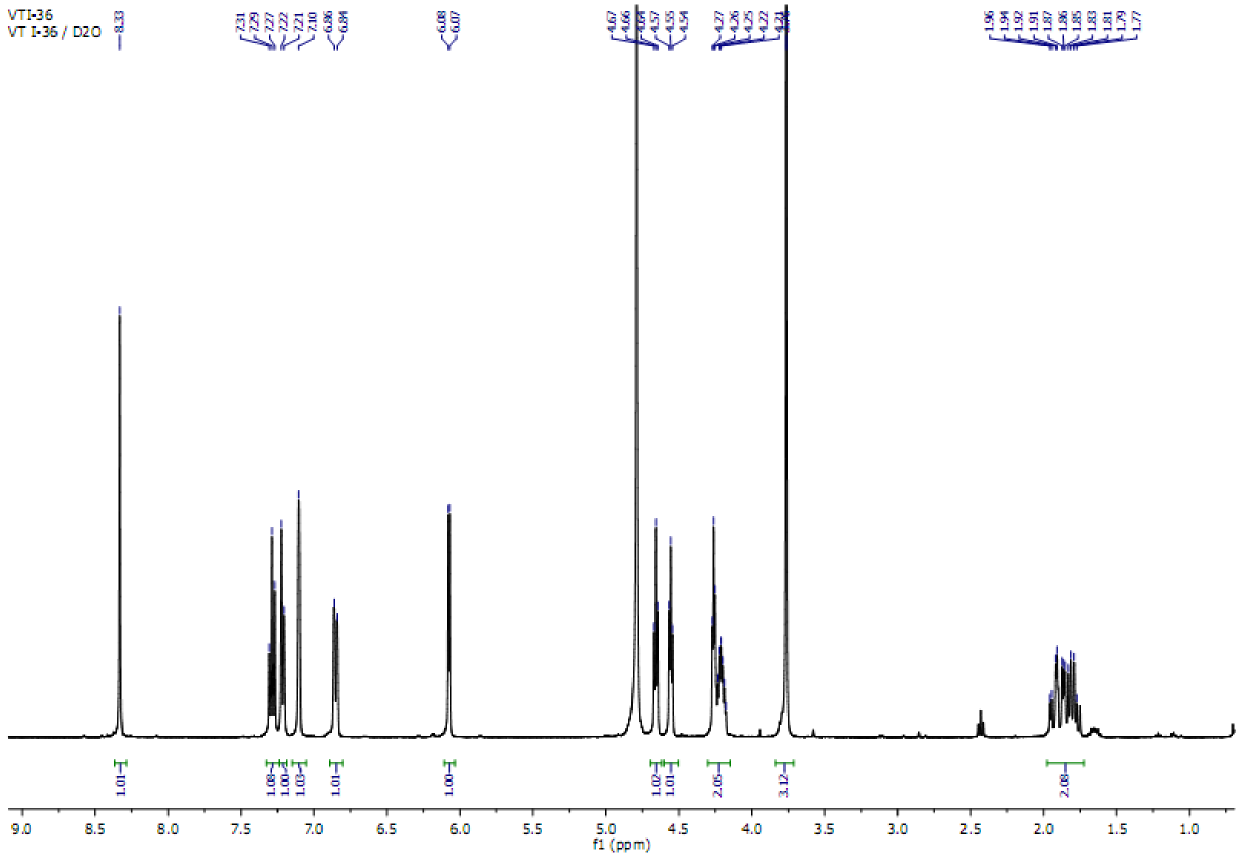

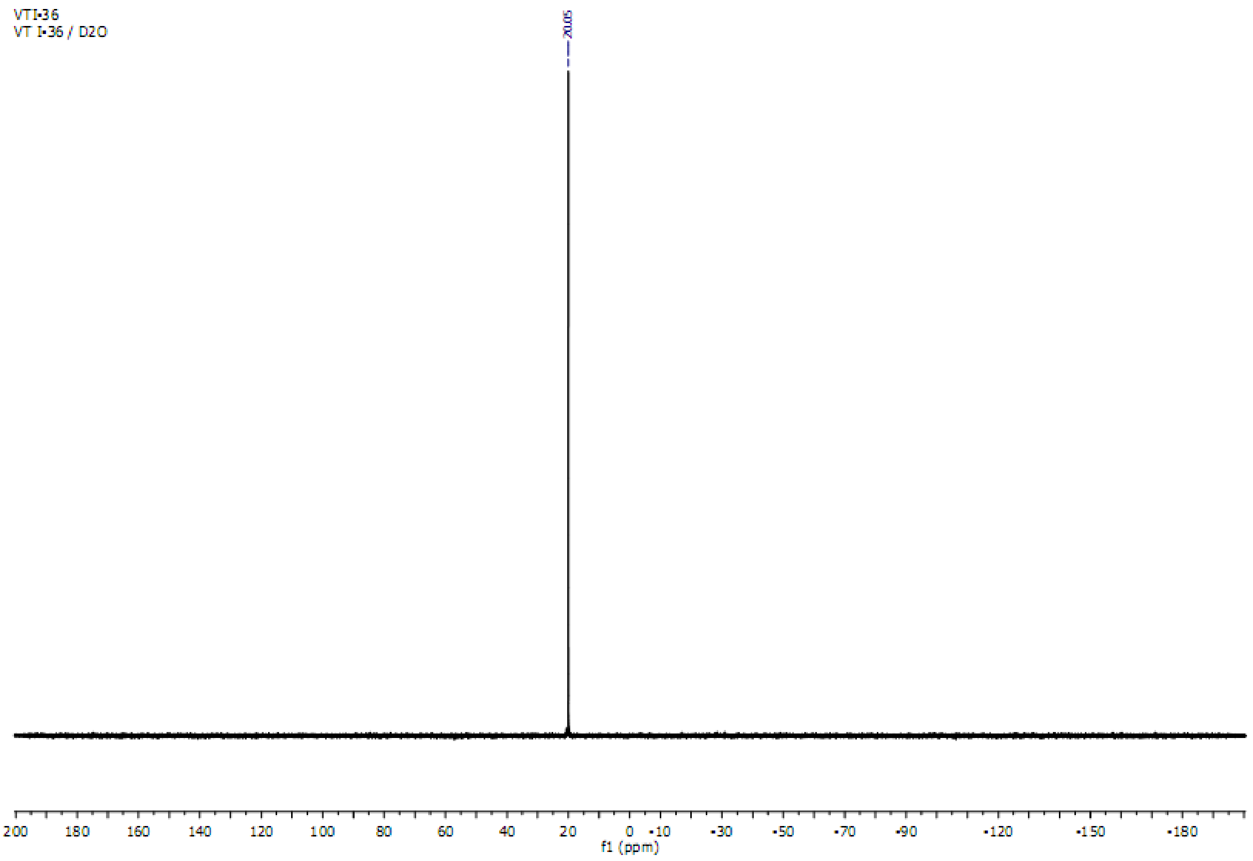

^{1}H NMR, ^{31}P NMR for compound **1c**

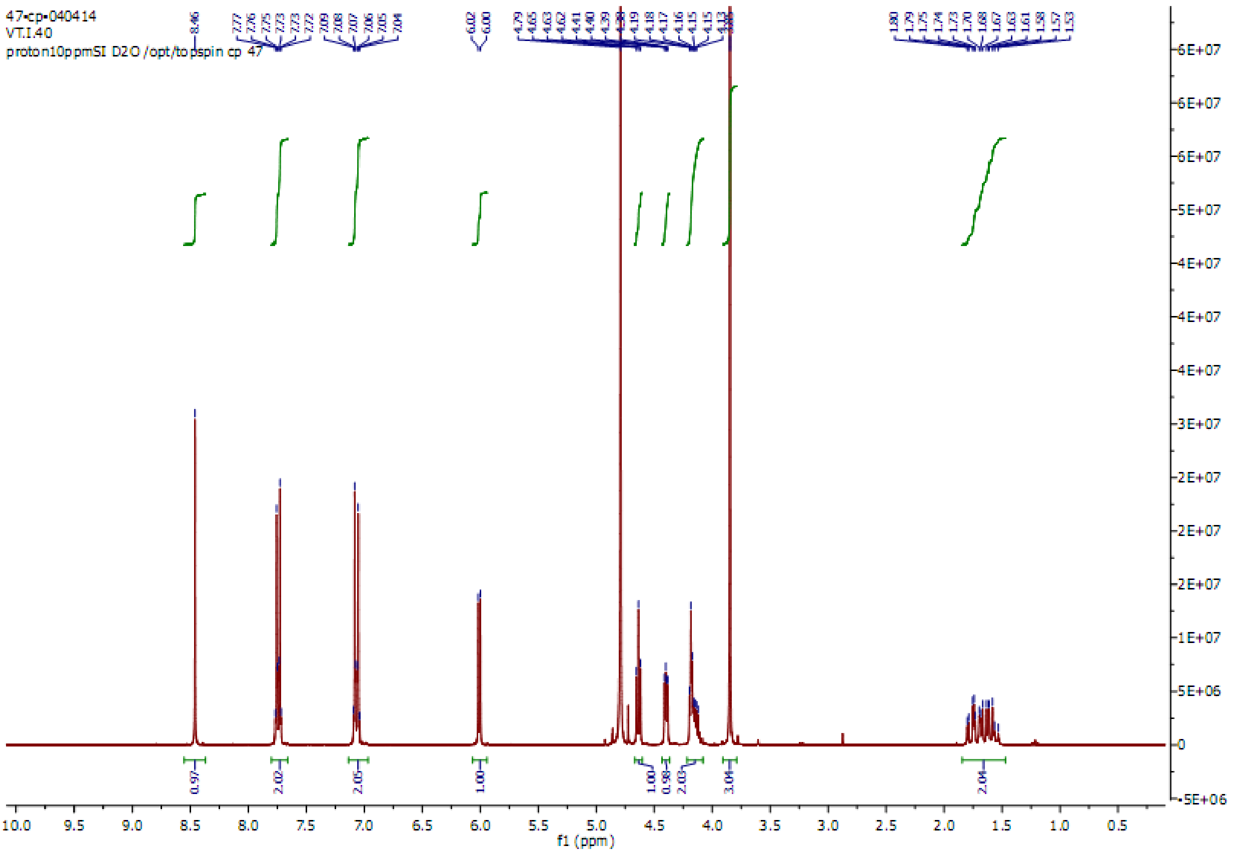

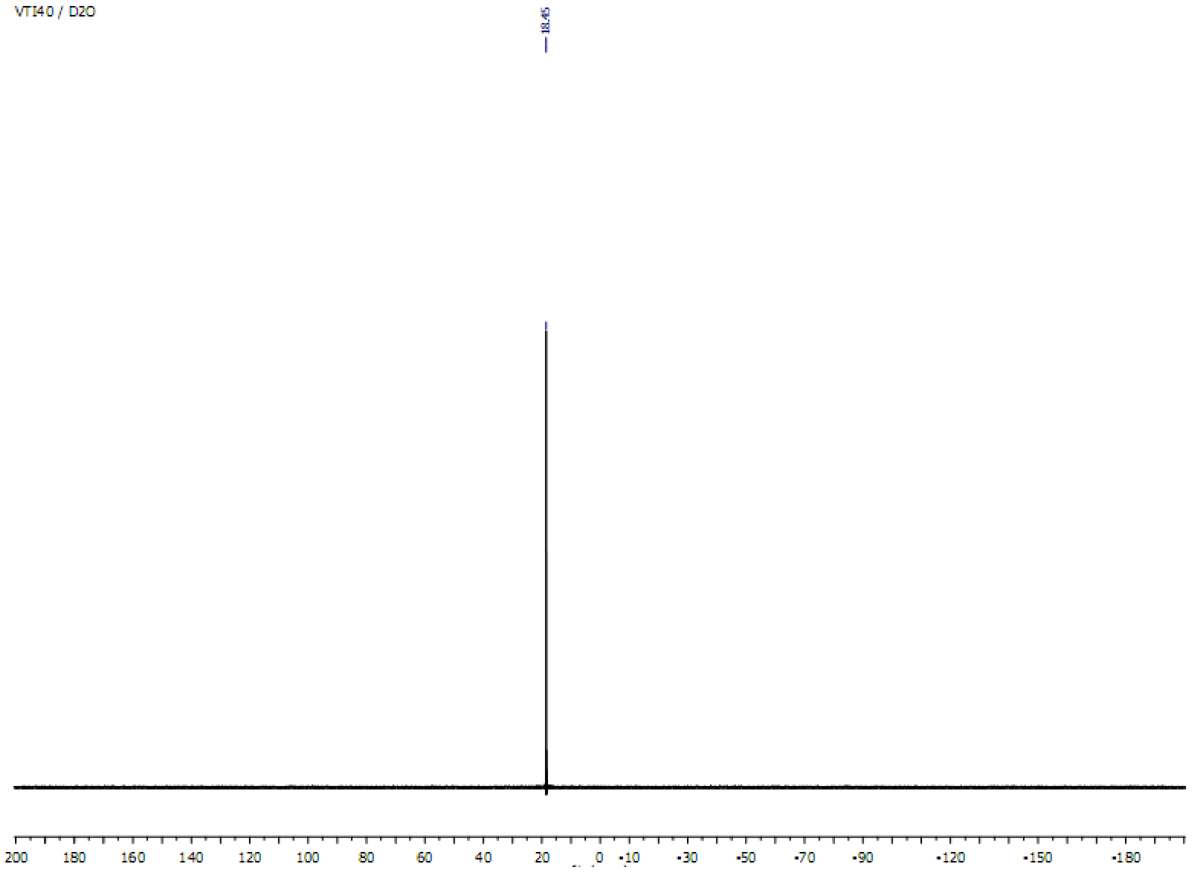

^{1}H NMR, ^{31}P NMR for compound **1d**

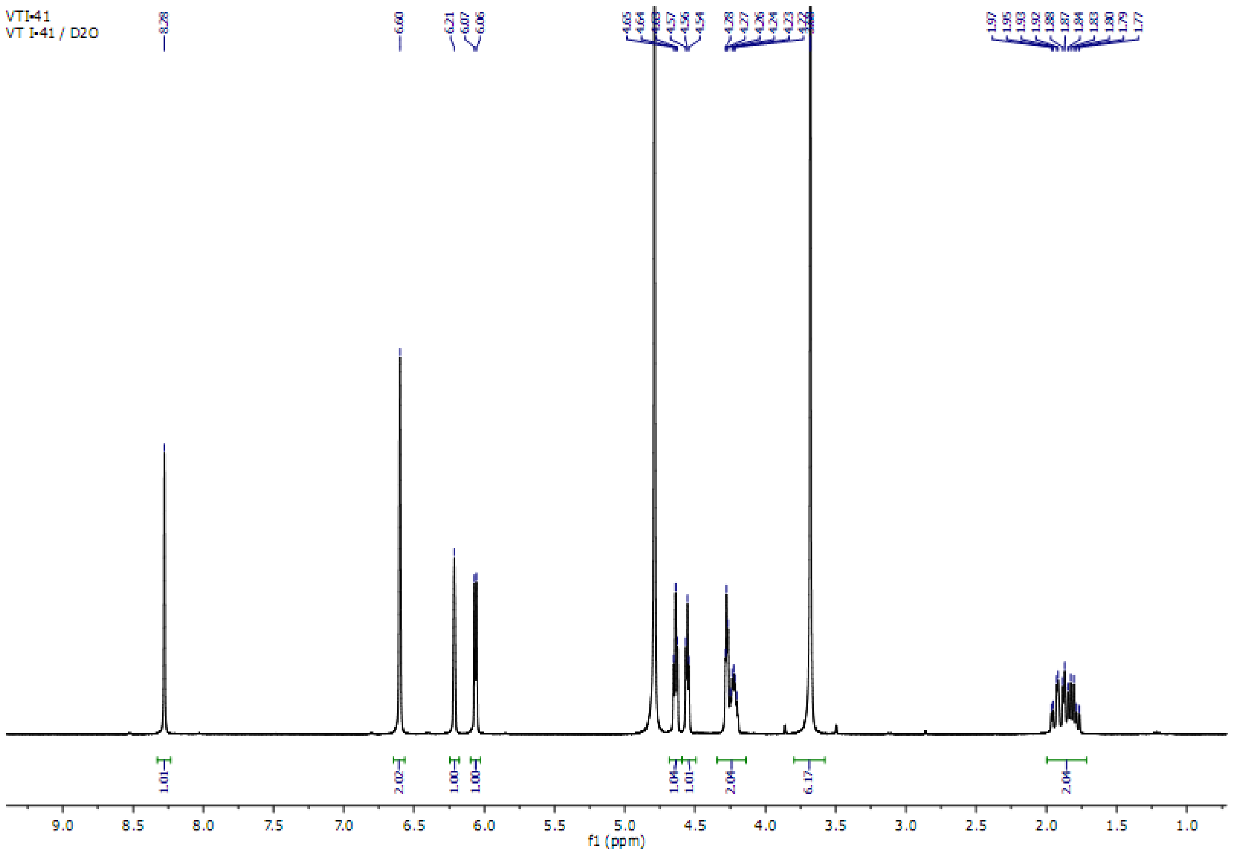

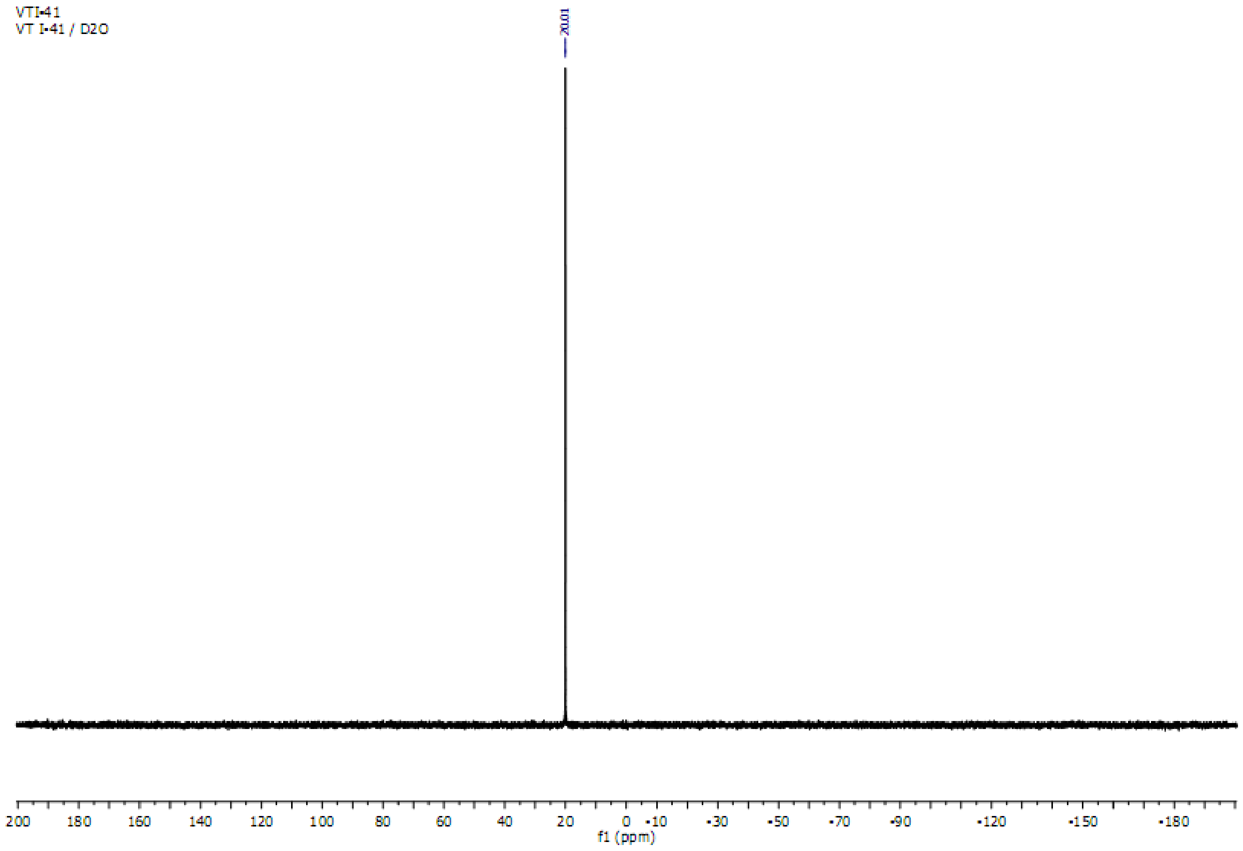

^{1}H NMR, ^{31}P NMR for compound **1e**

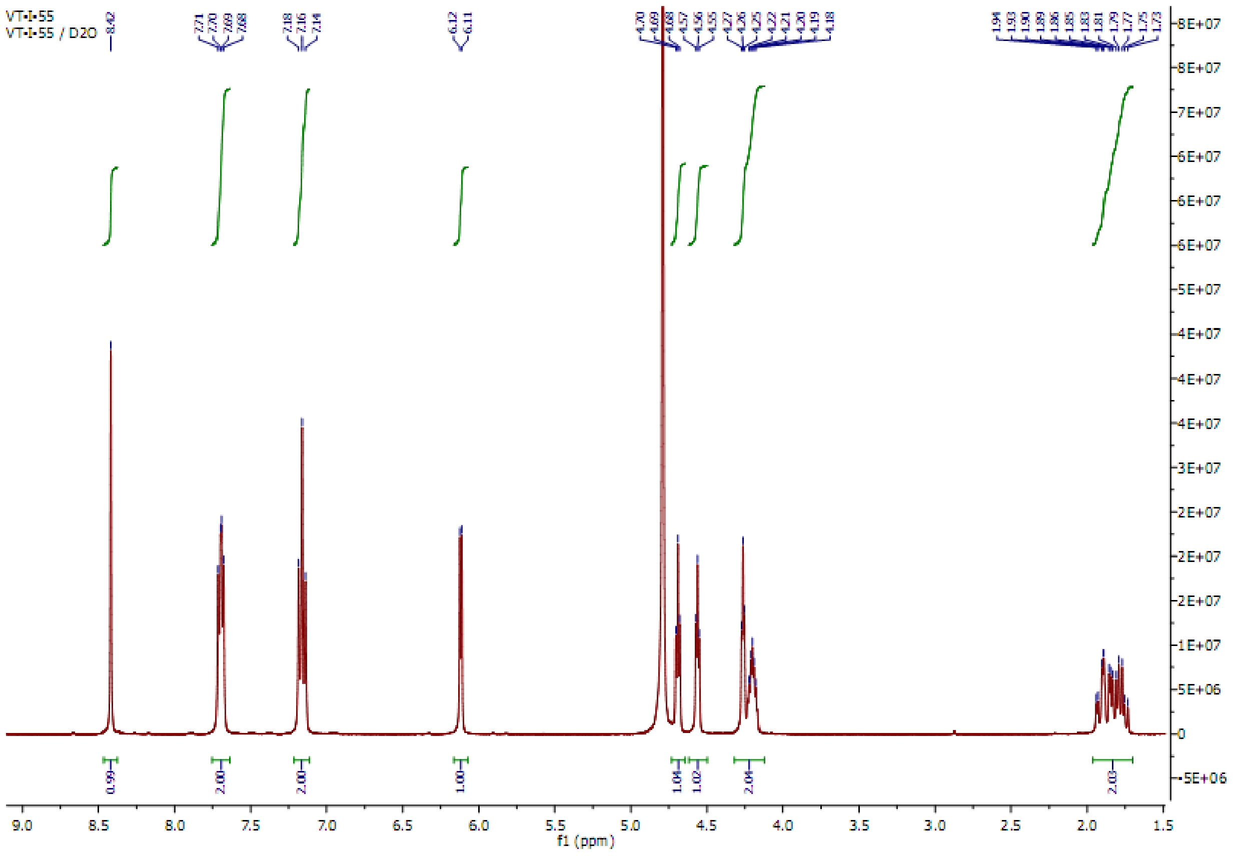

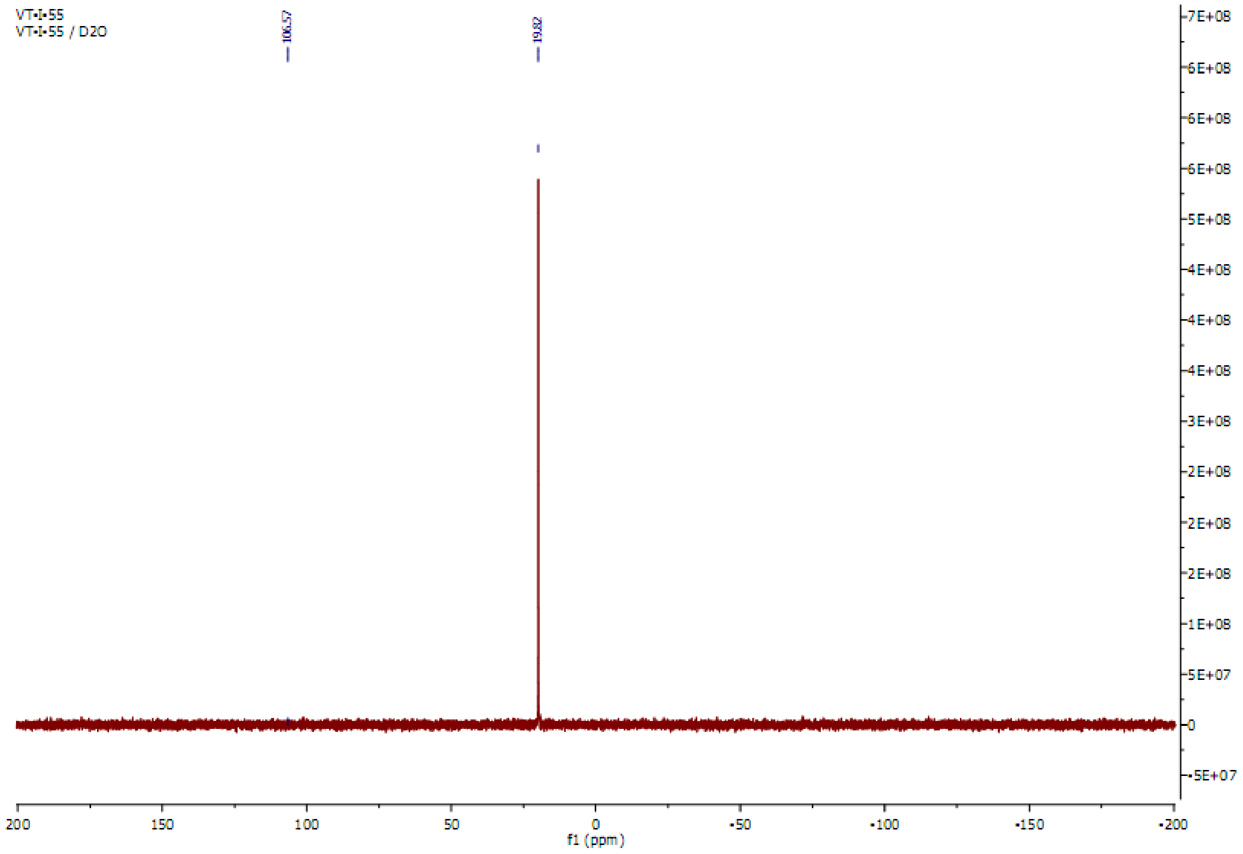

^{1}H NMR, ^{31}P NMR for compound **1f**

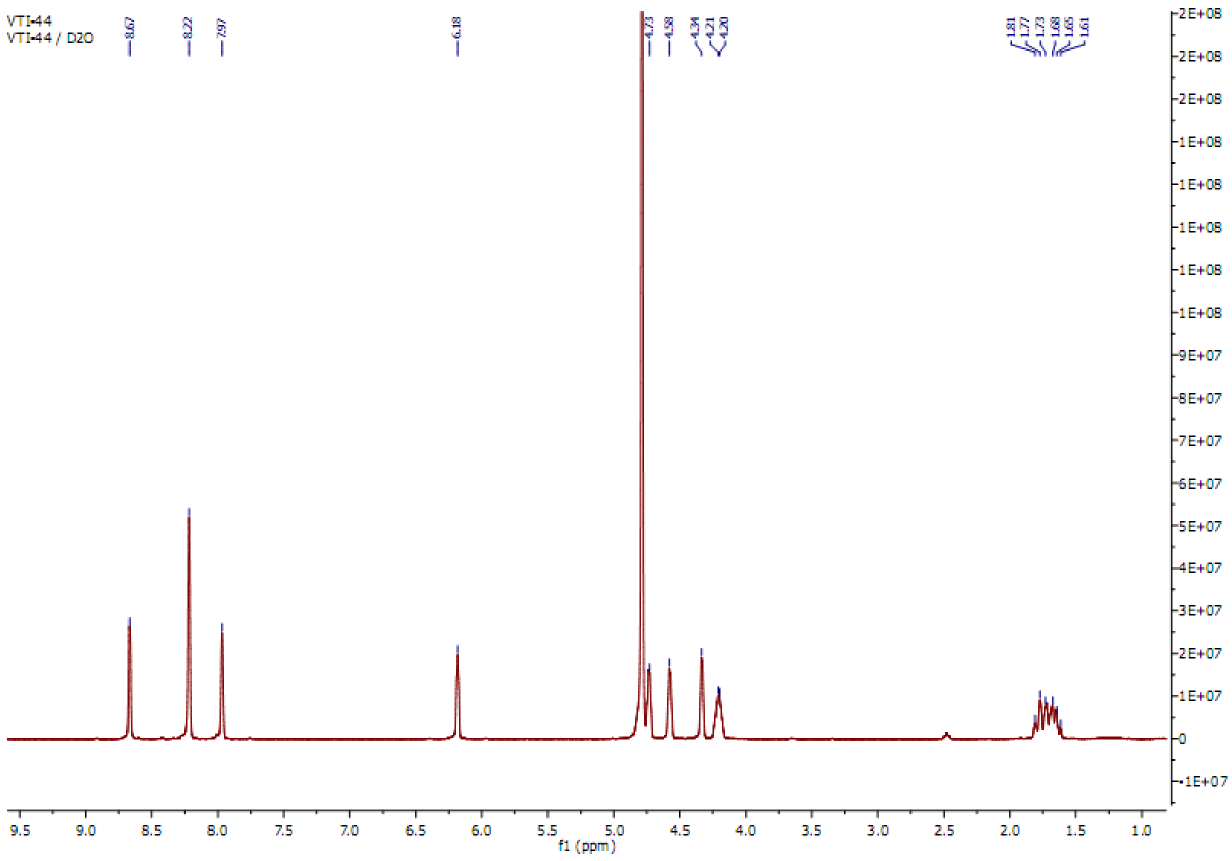

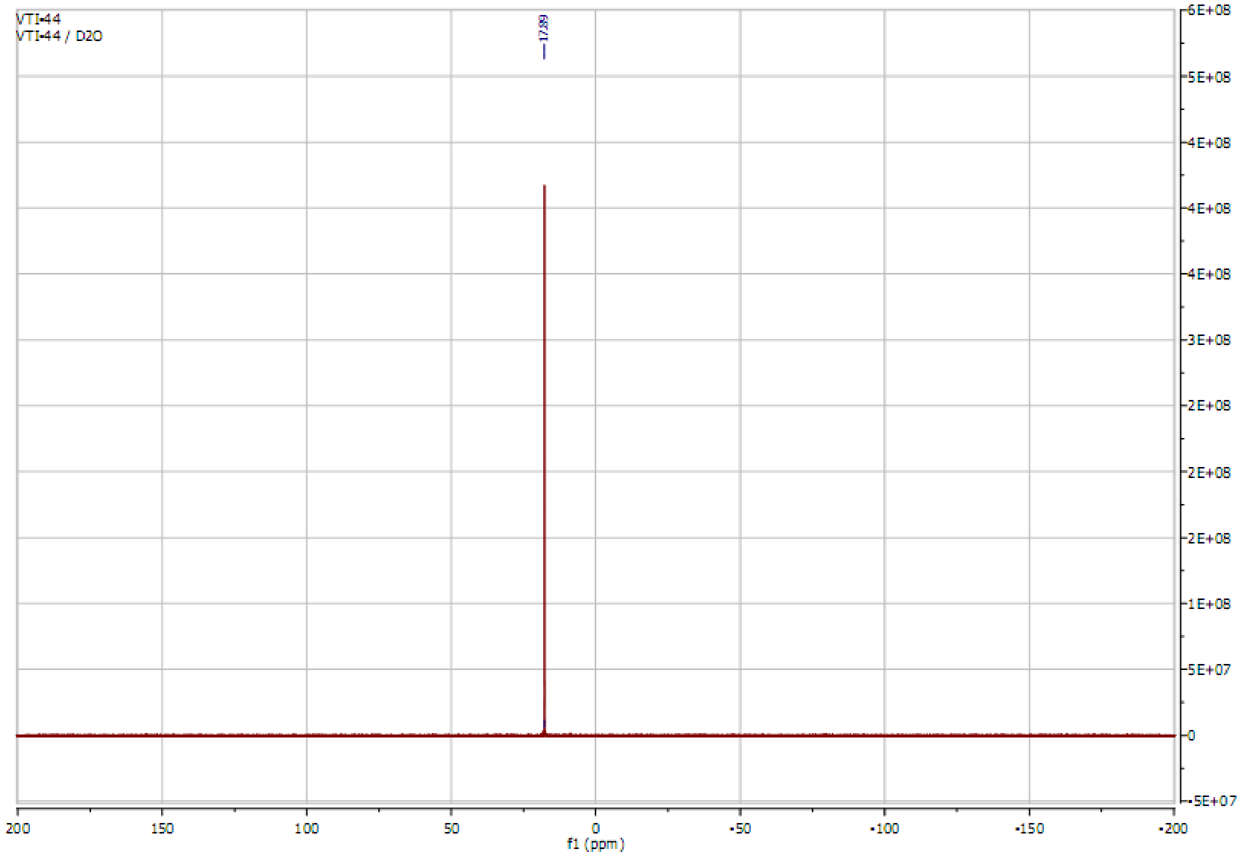

^{1}H NMR, ^{31}P NMR for compound **1g**

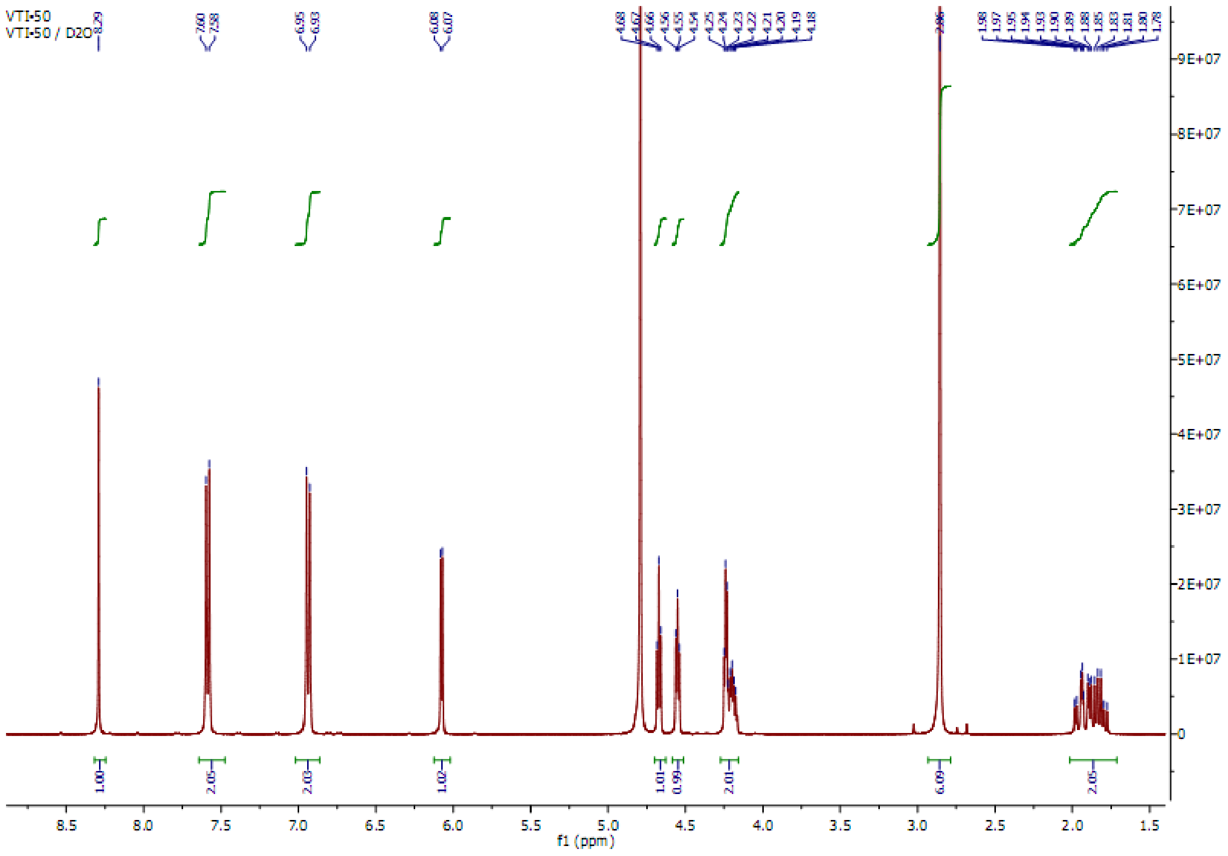

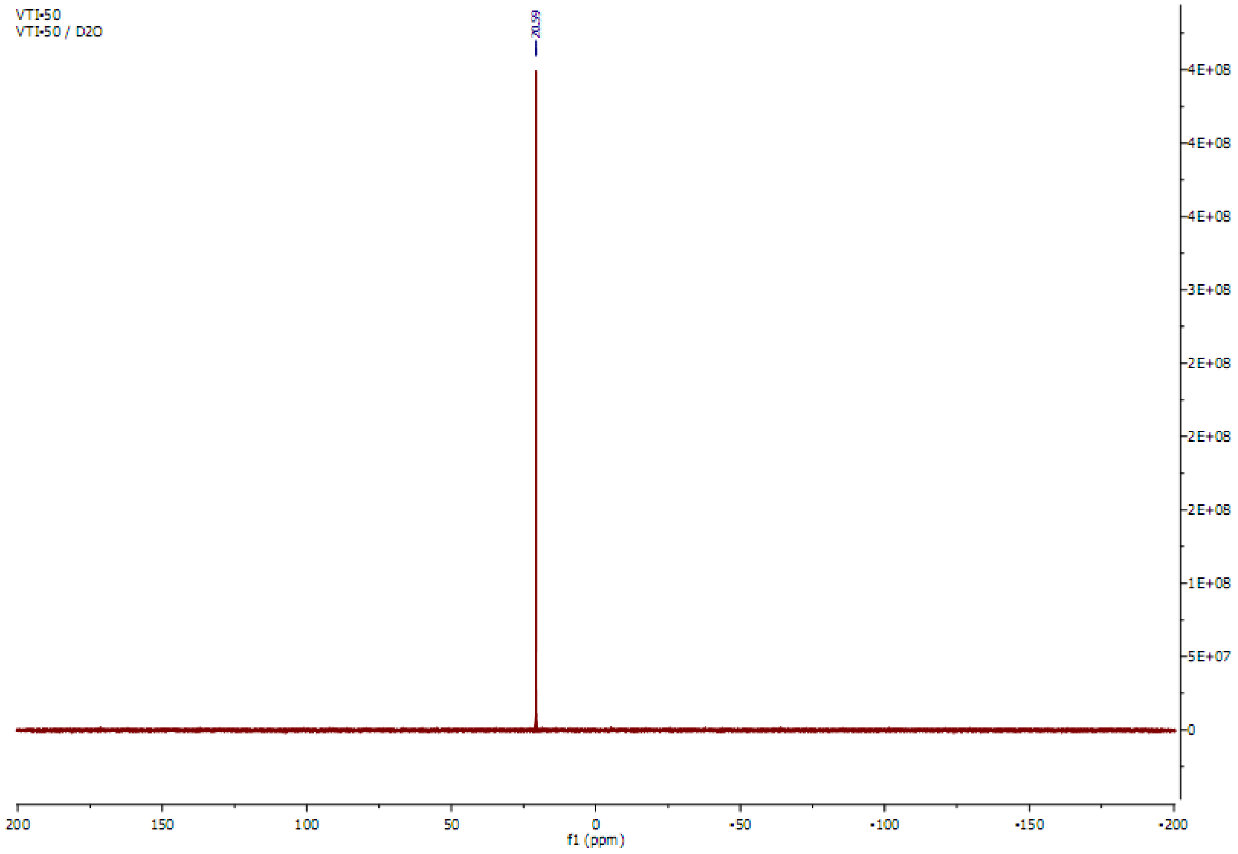

^{1}H NMR, ^{31}P NMR for compound **1h**

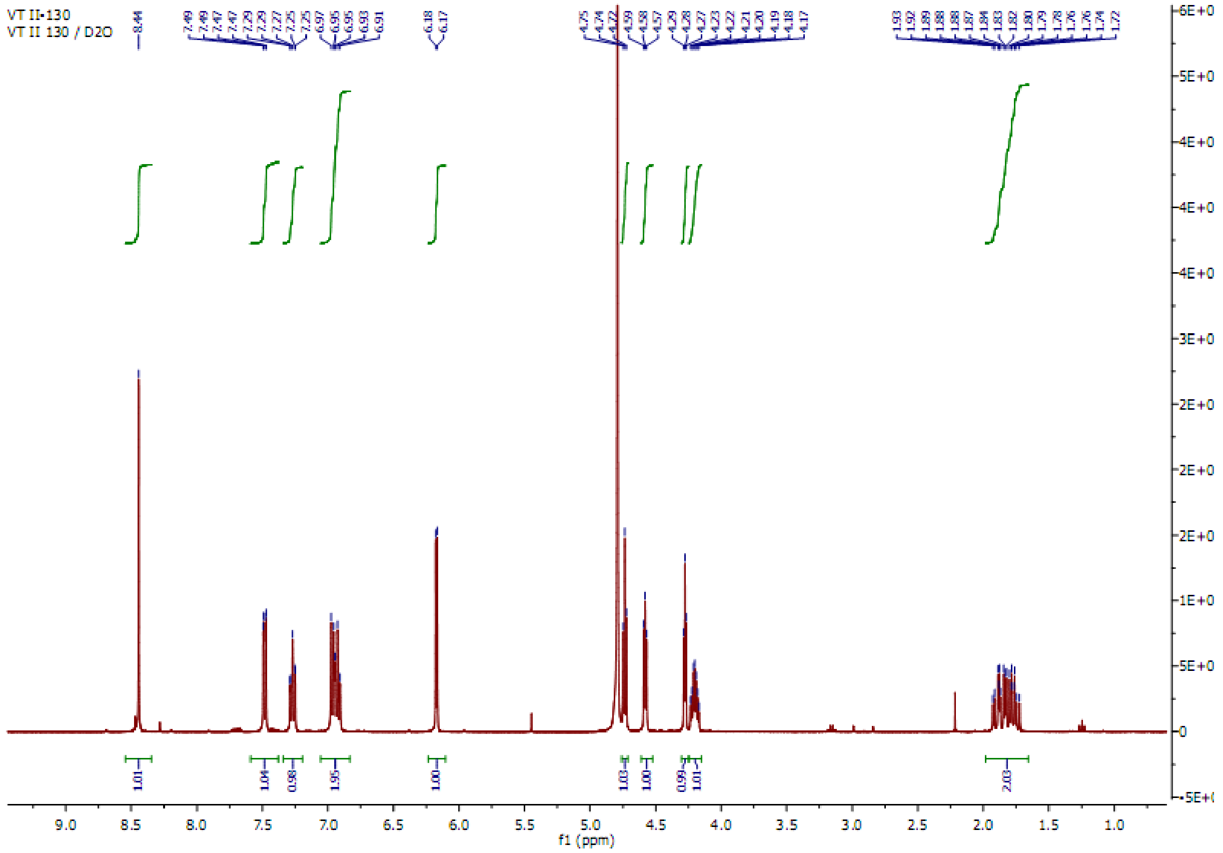

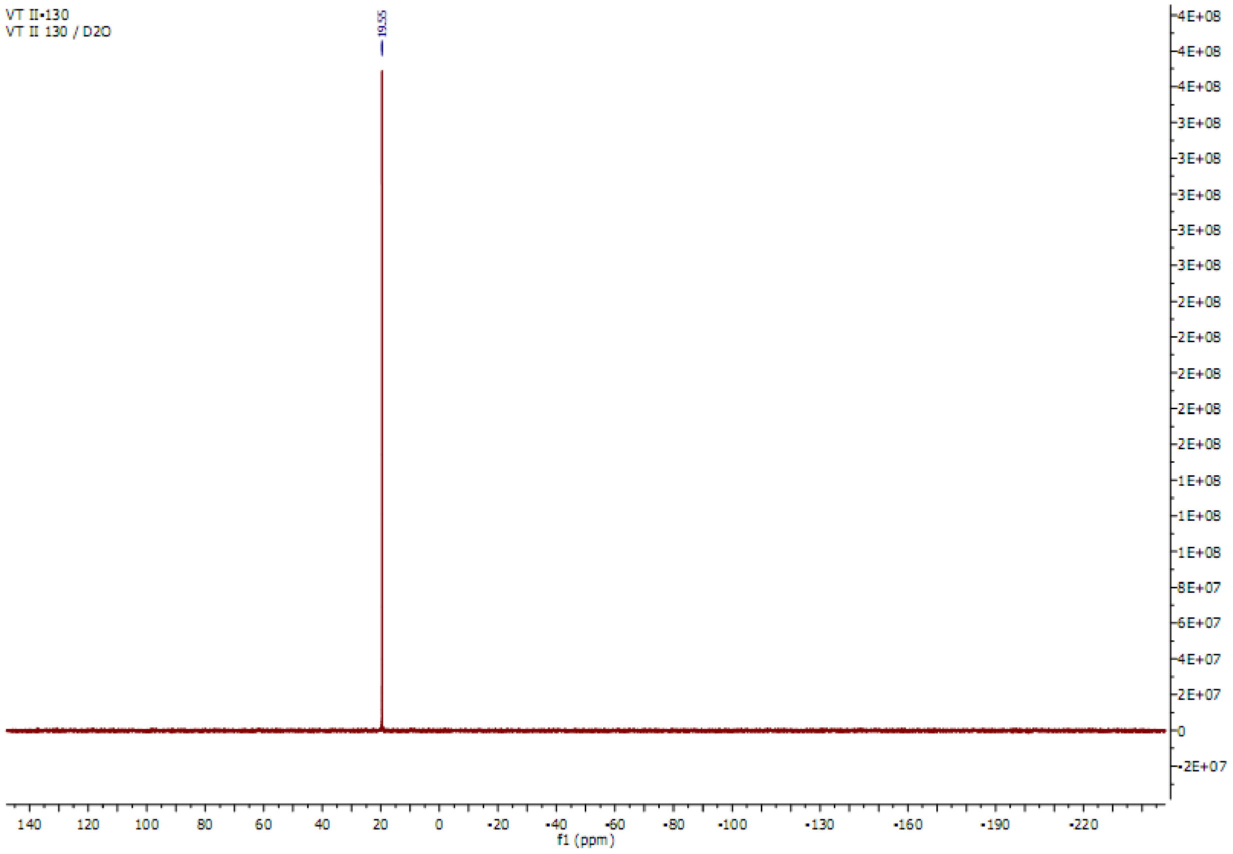

^{1}H NMR, ^{31}P NMR for compound **1i**

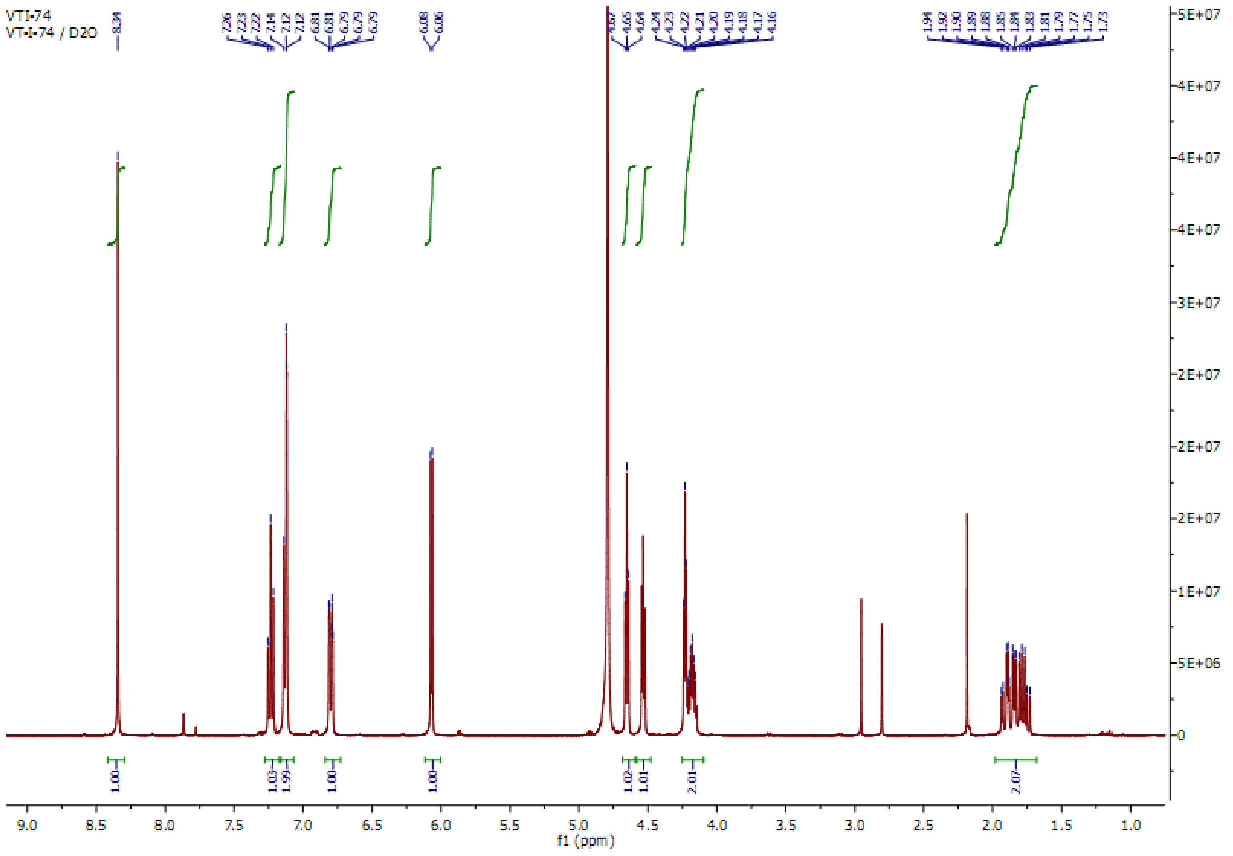

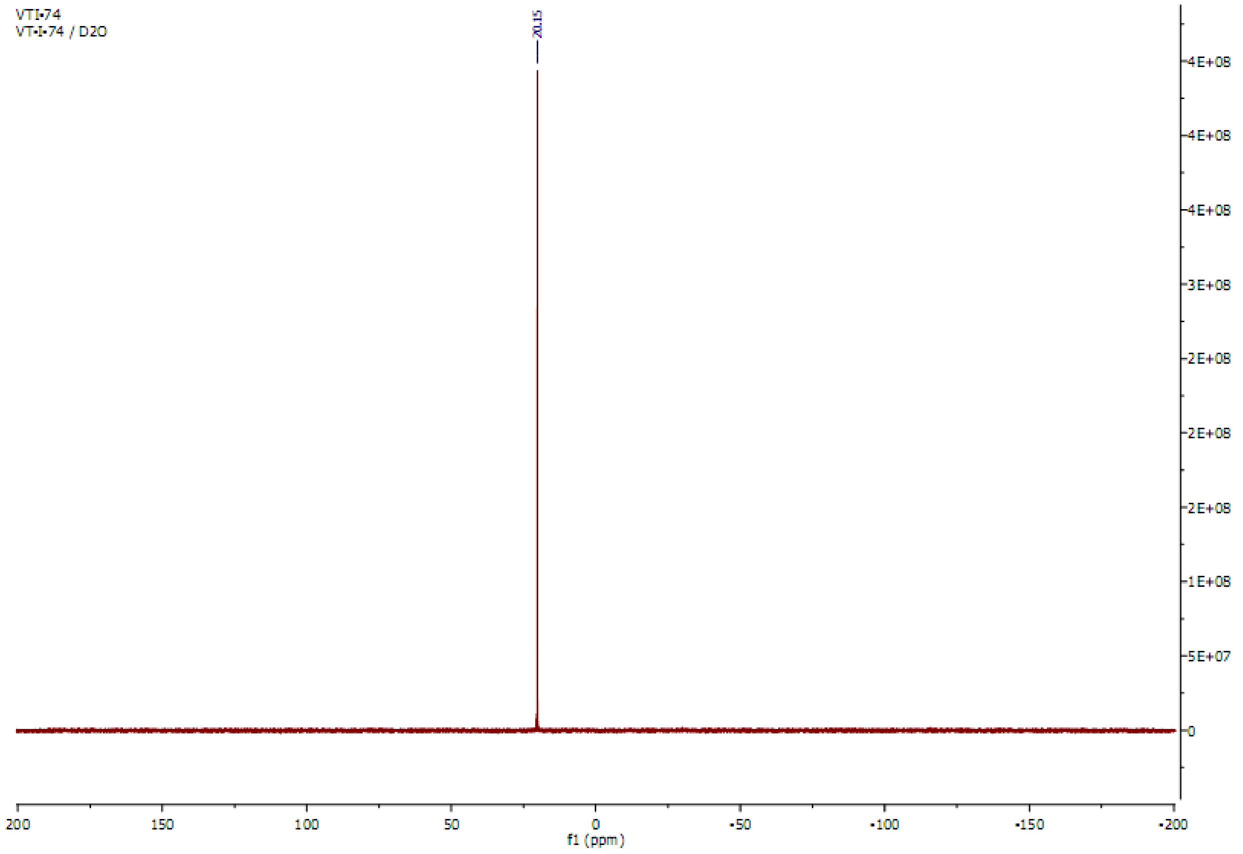

^{1}H NMR, ^{31}P NMR for compound **1j**

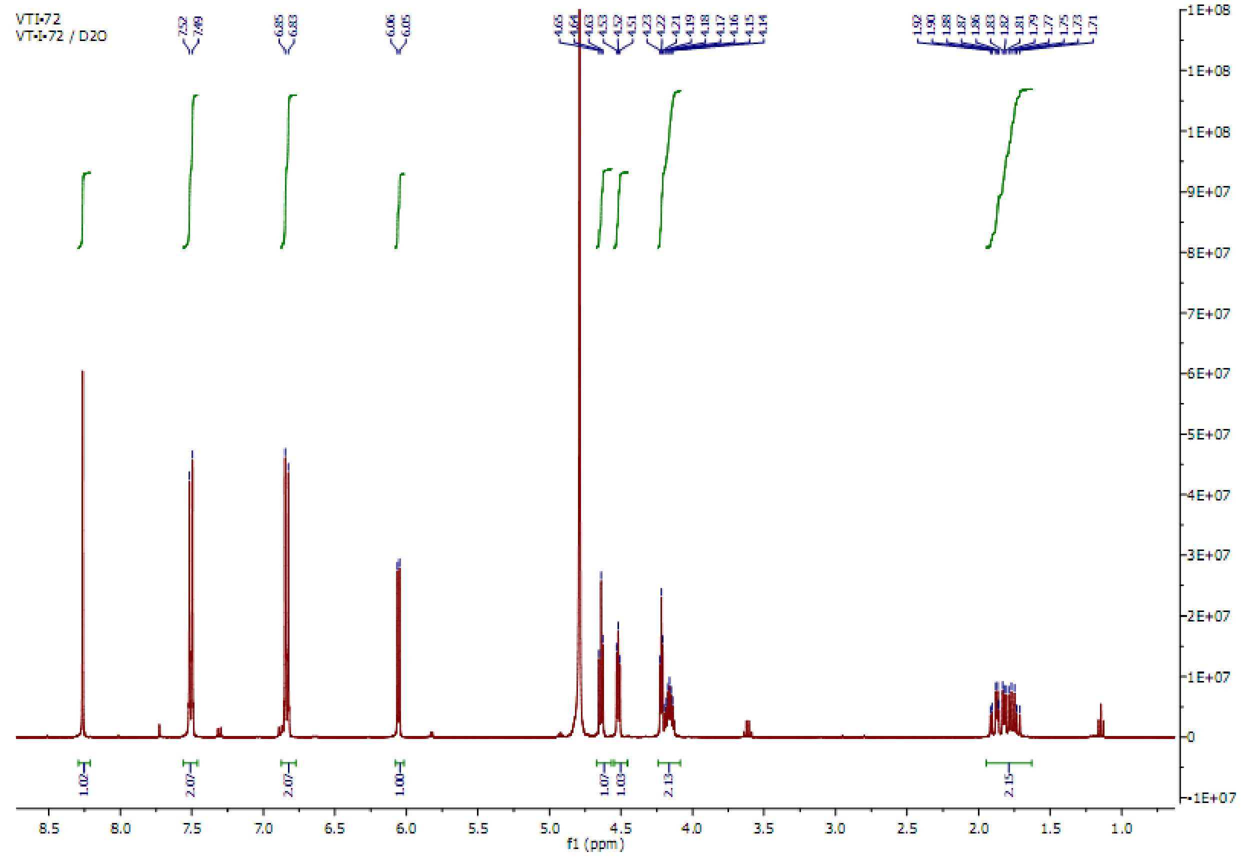

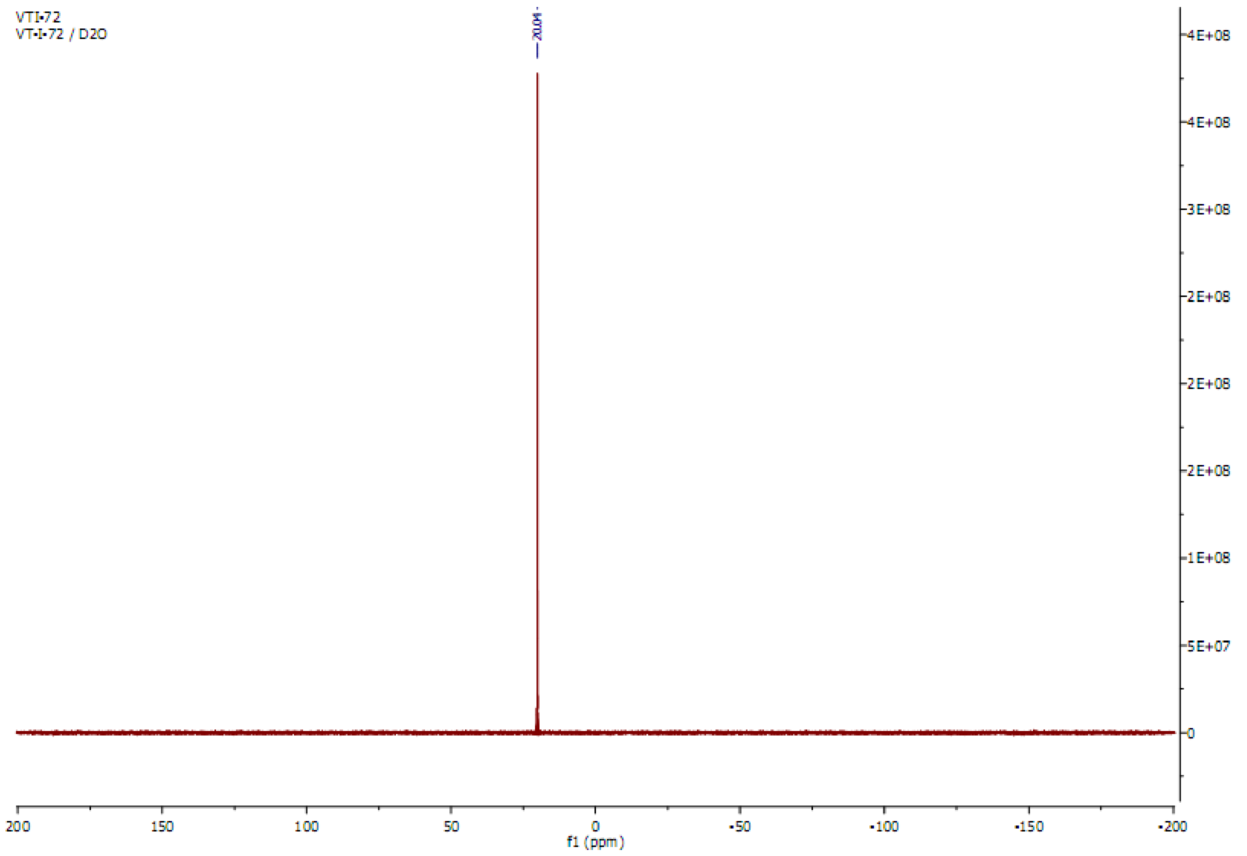

^{1}H NMR, ^{31}P NMR for compound **1k**

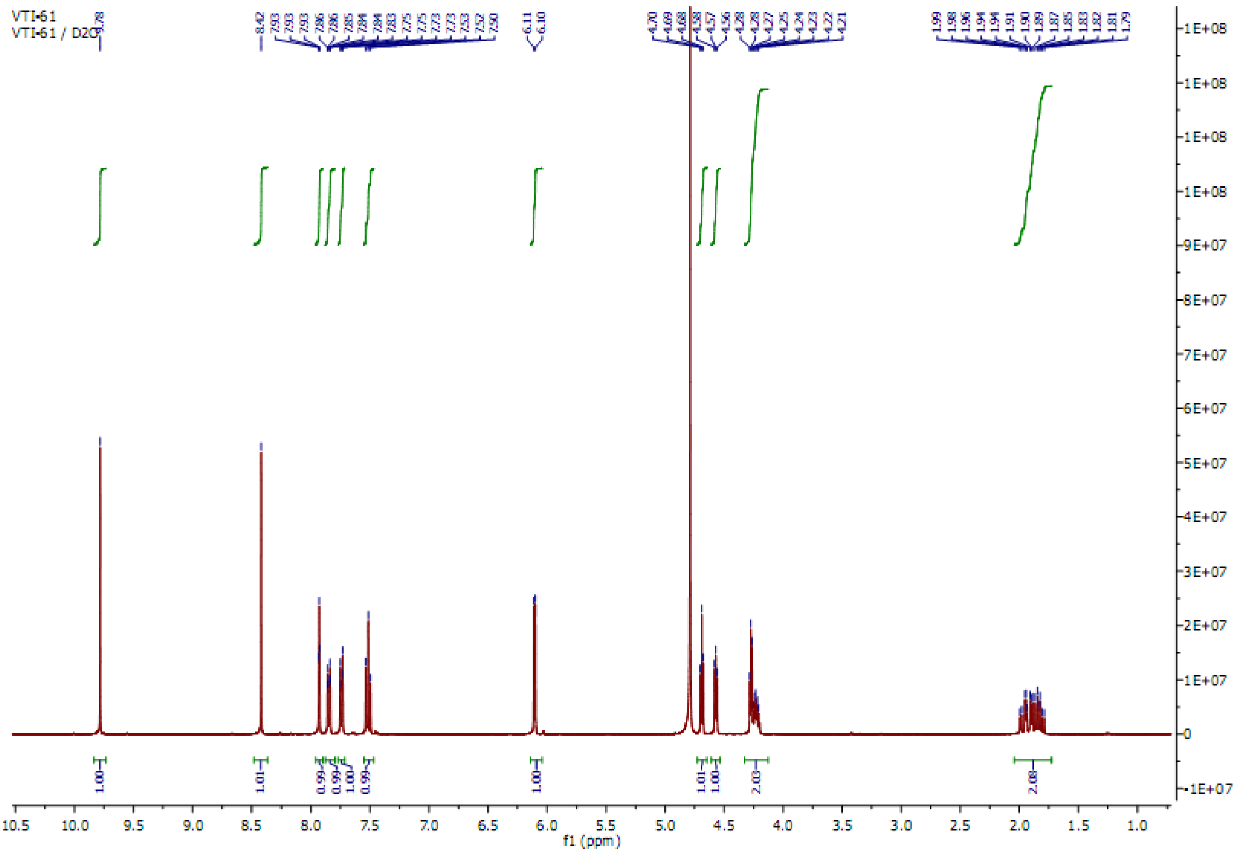

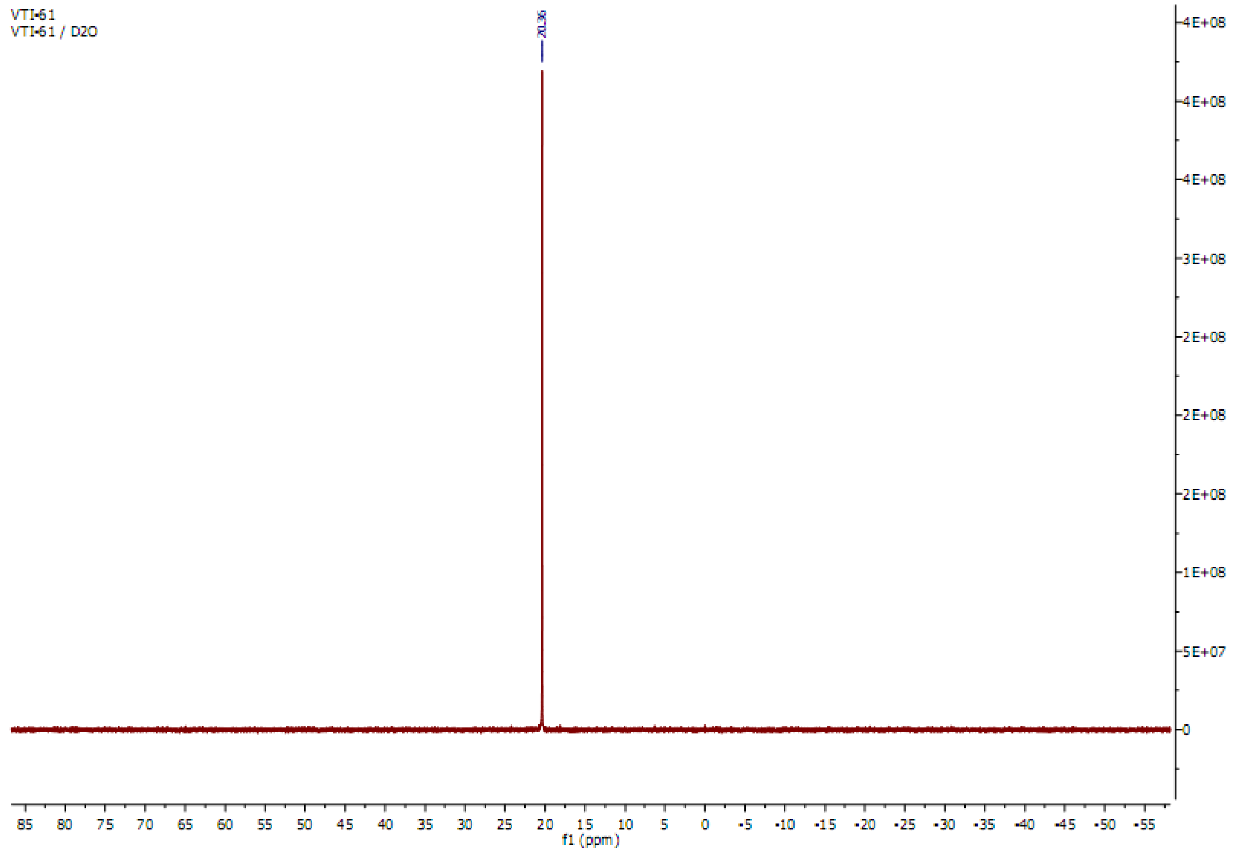

^{1}H NMR, ^{31}P NMR for compound **1l**

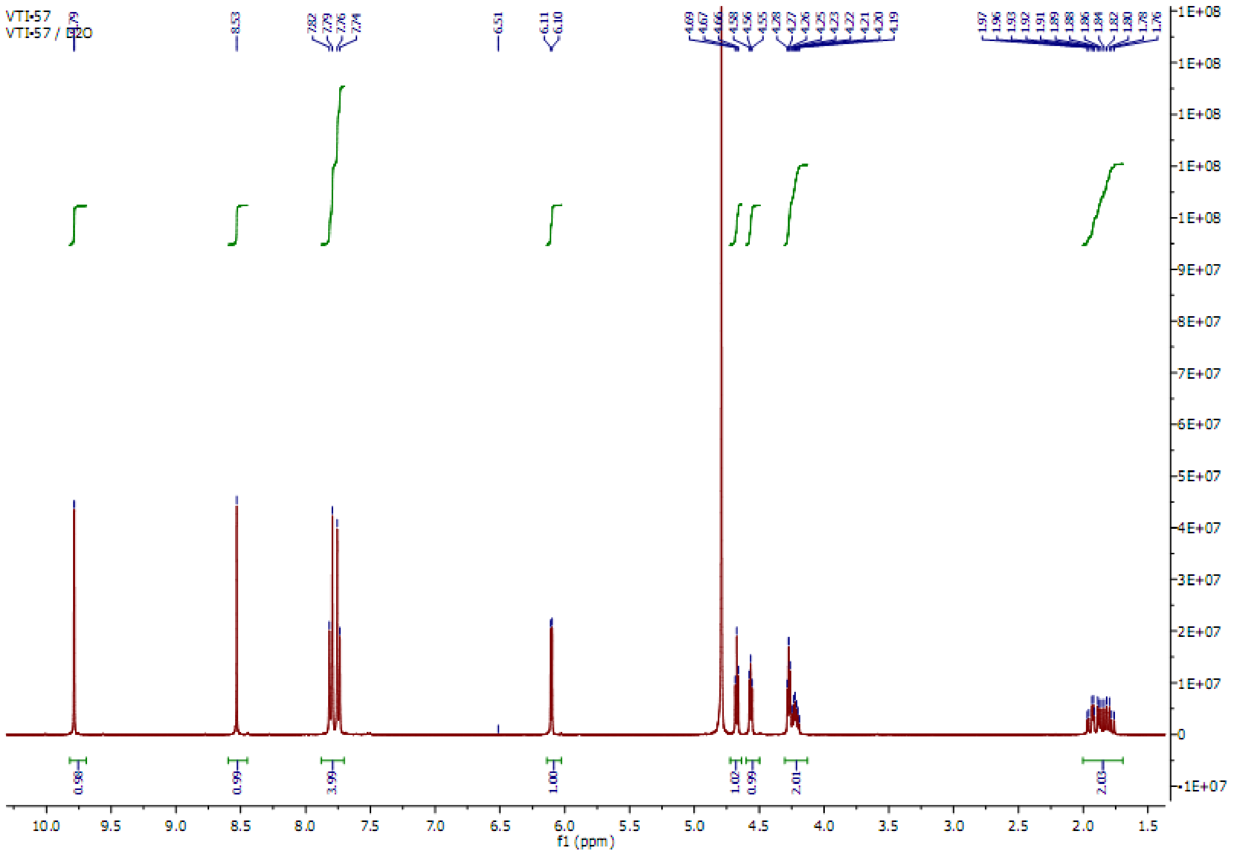

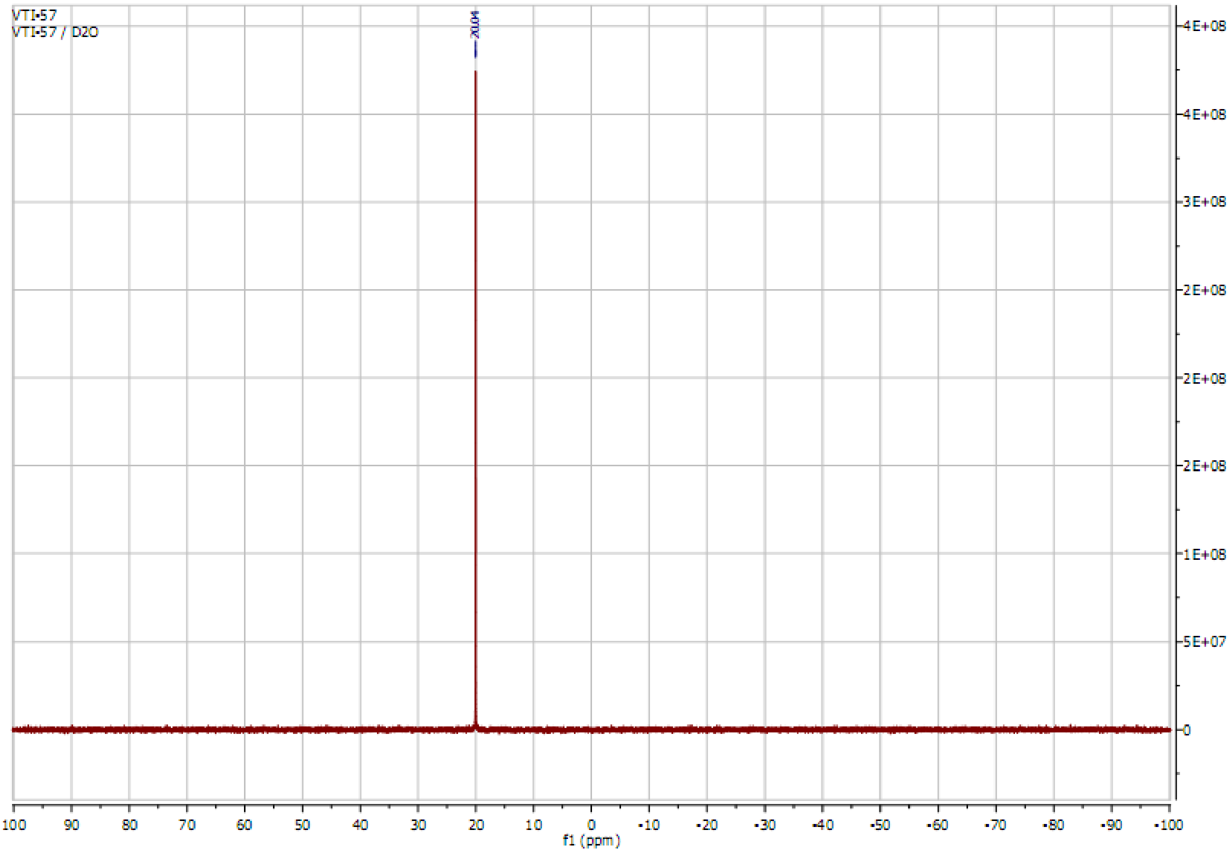

^{1}H NMR, ^{31}P NMR for compound **1m**

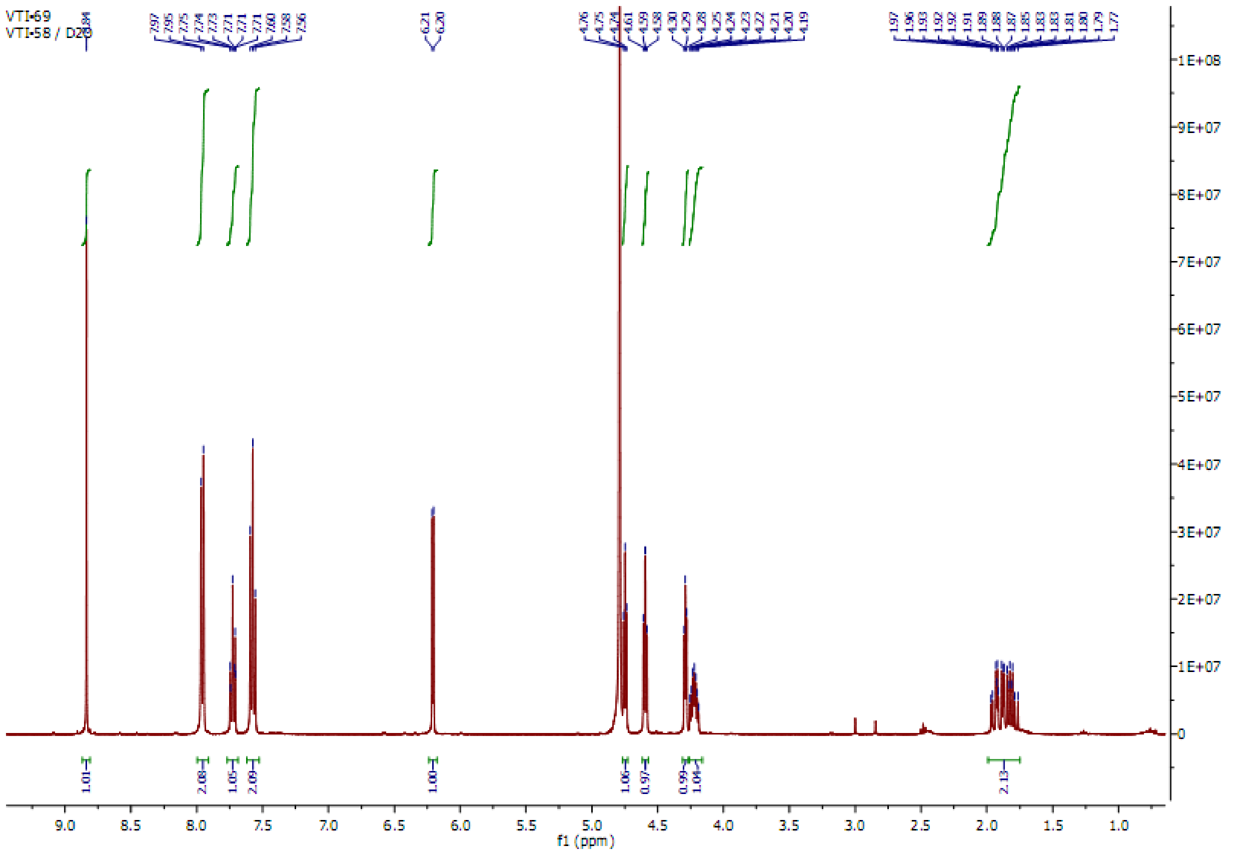

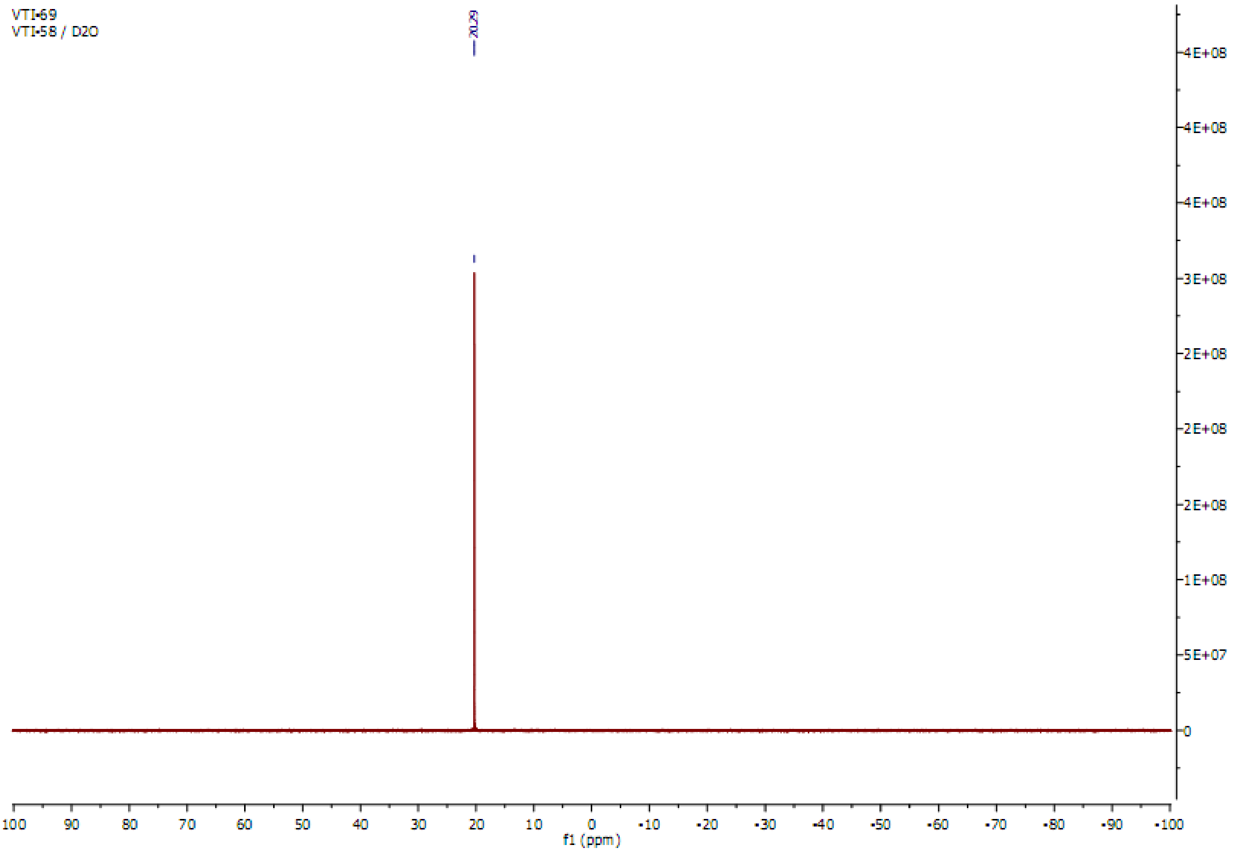

^{1}H NMR, ^{31}P NMR for compound **1n**

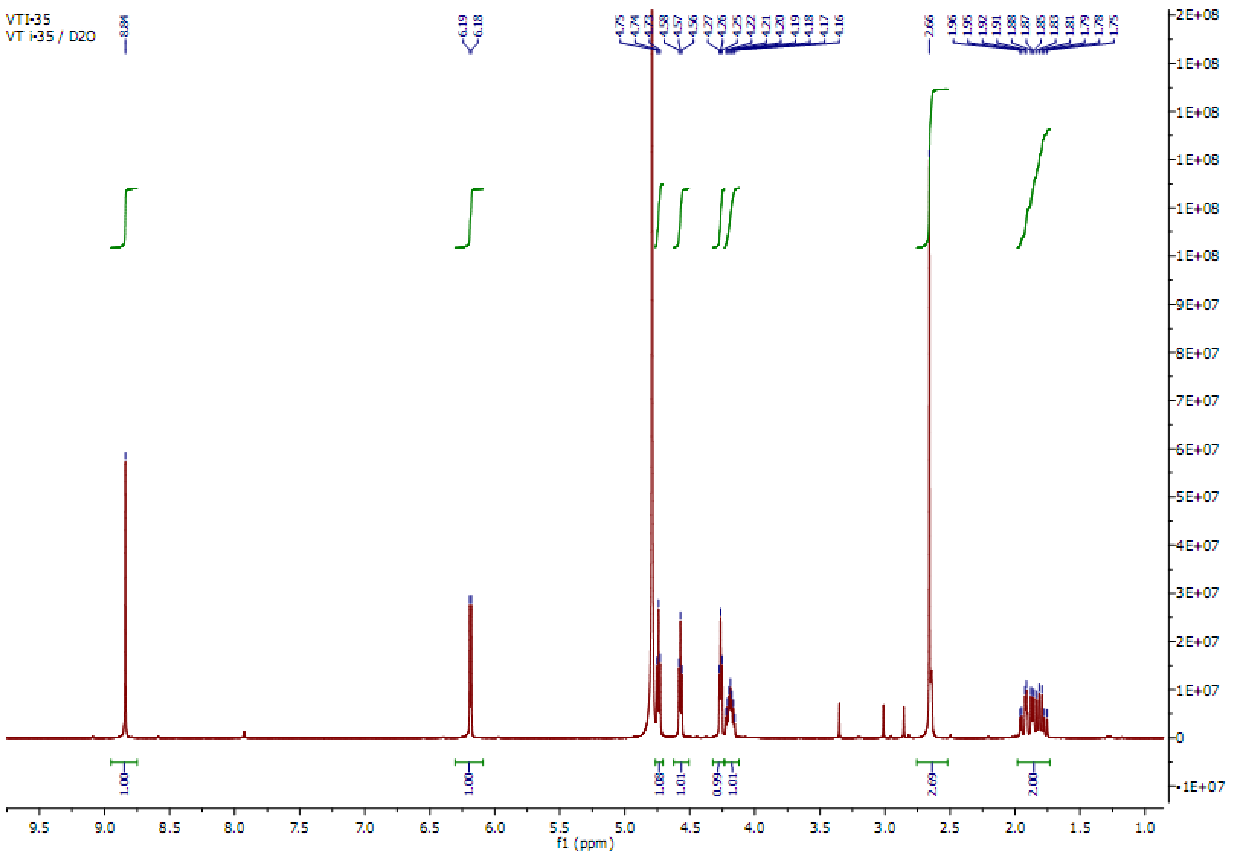

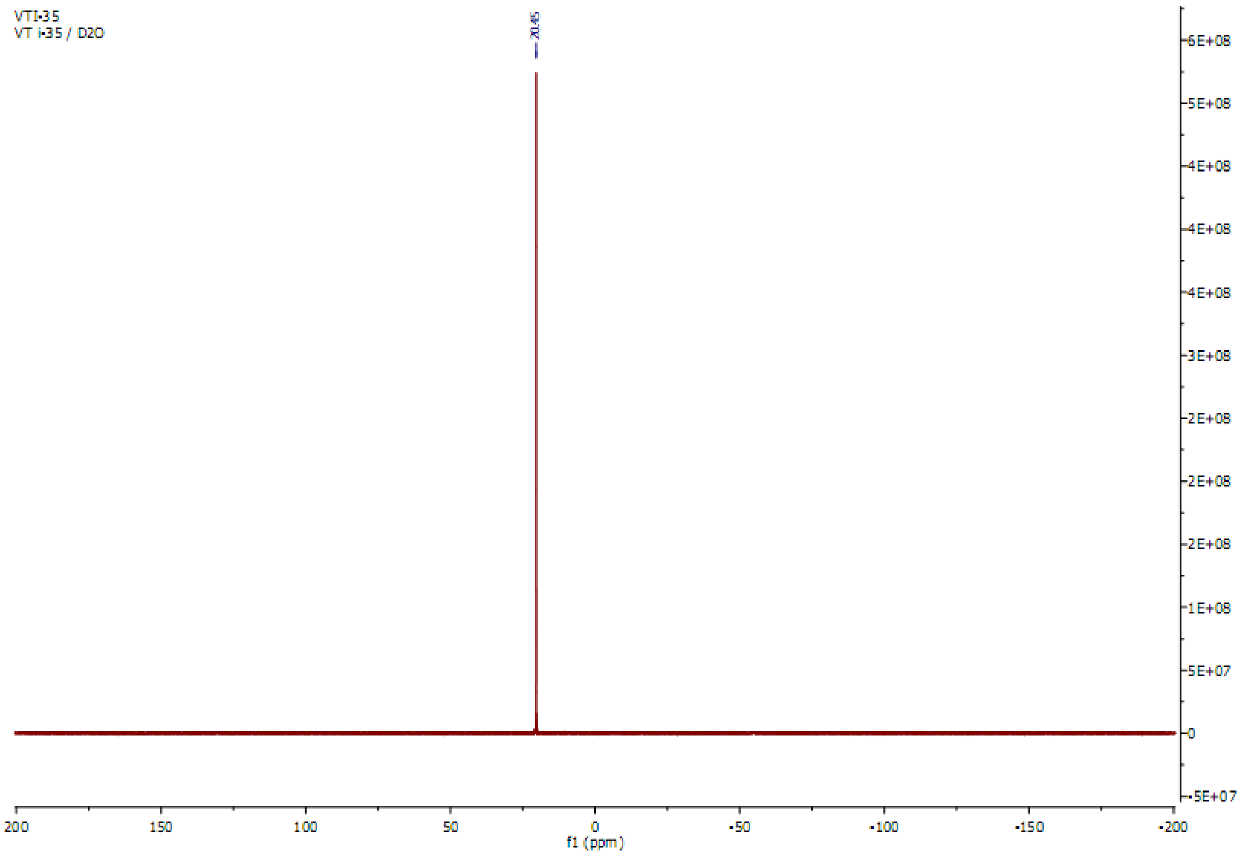

^{1}H NMR, ^{31}P NMR for compound **1o**

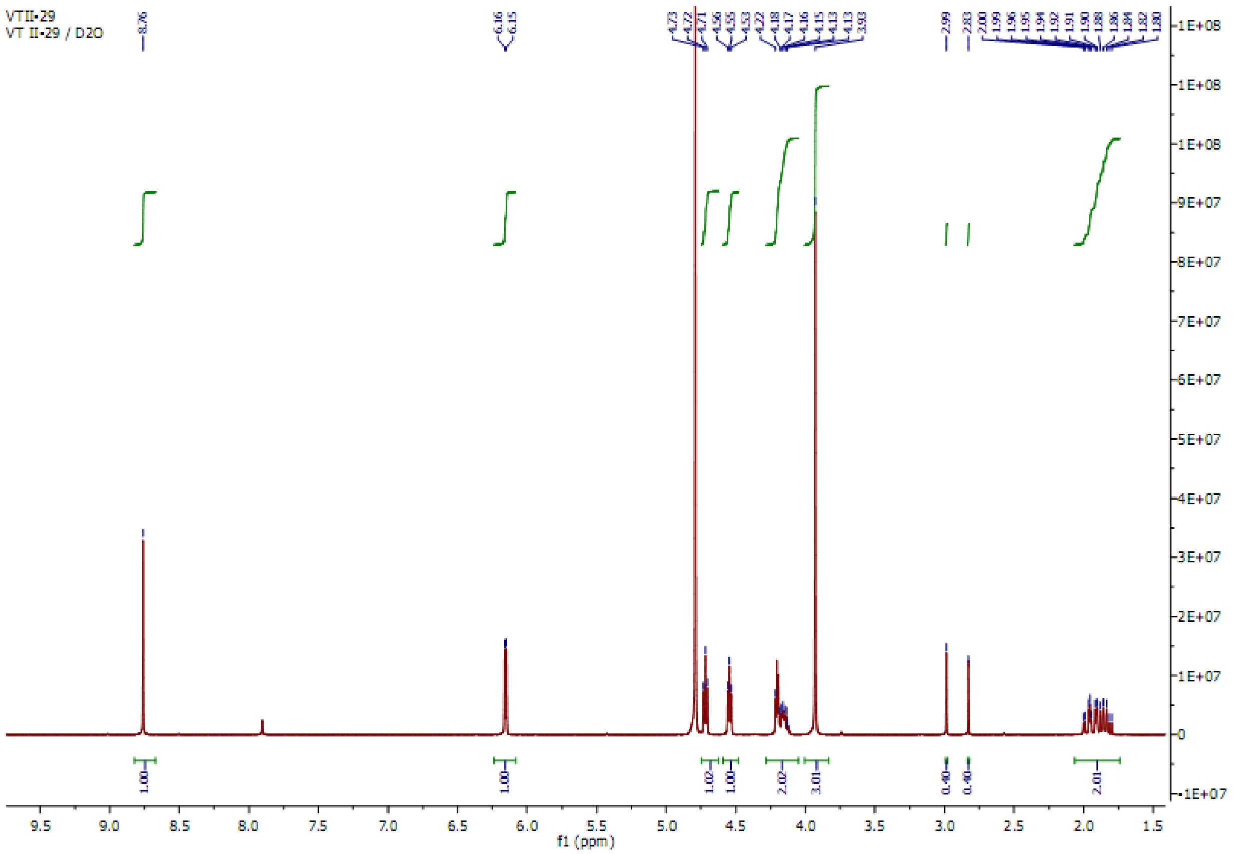

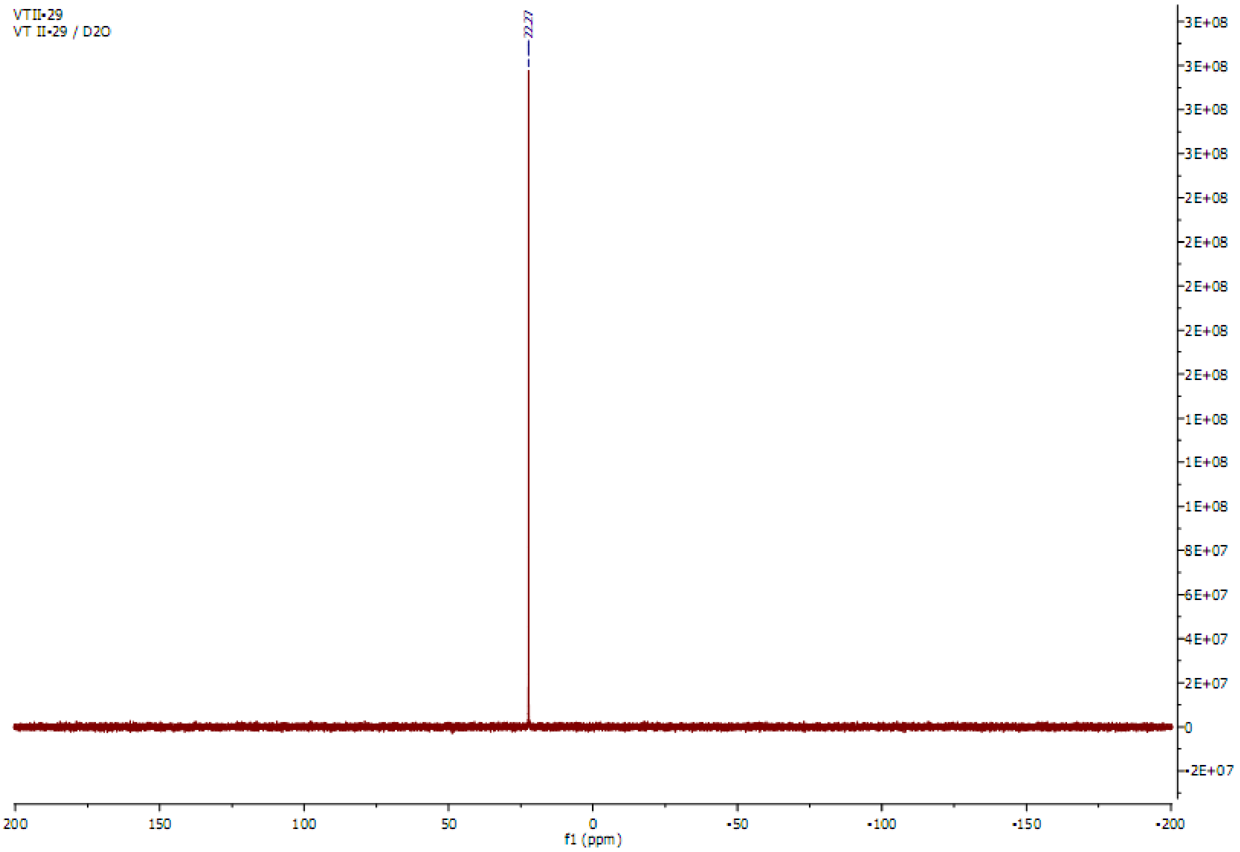

^{1}H NMR, ^{31}P NMR for compound **1p**

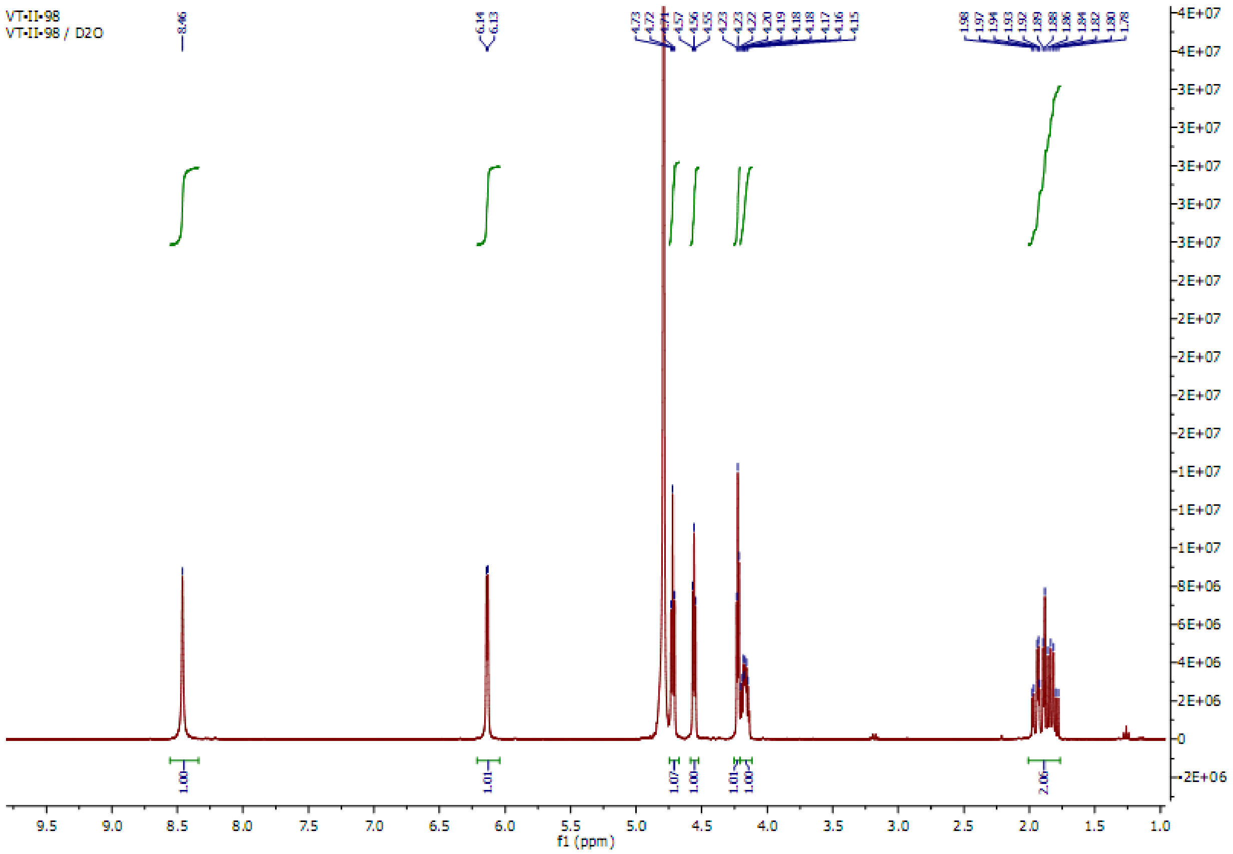

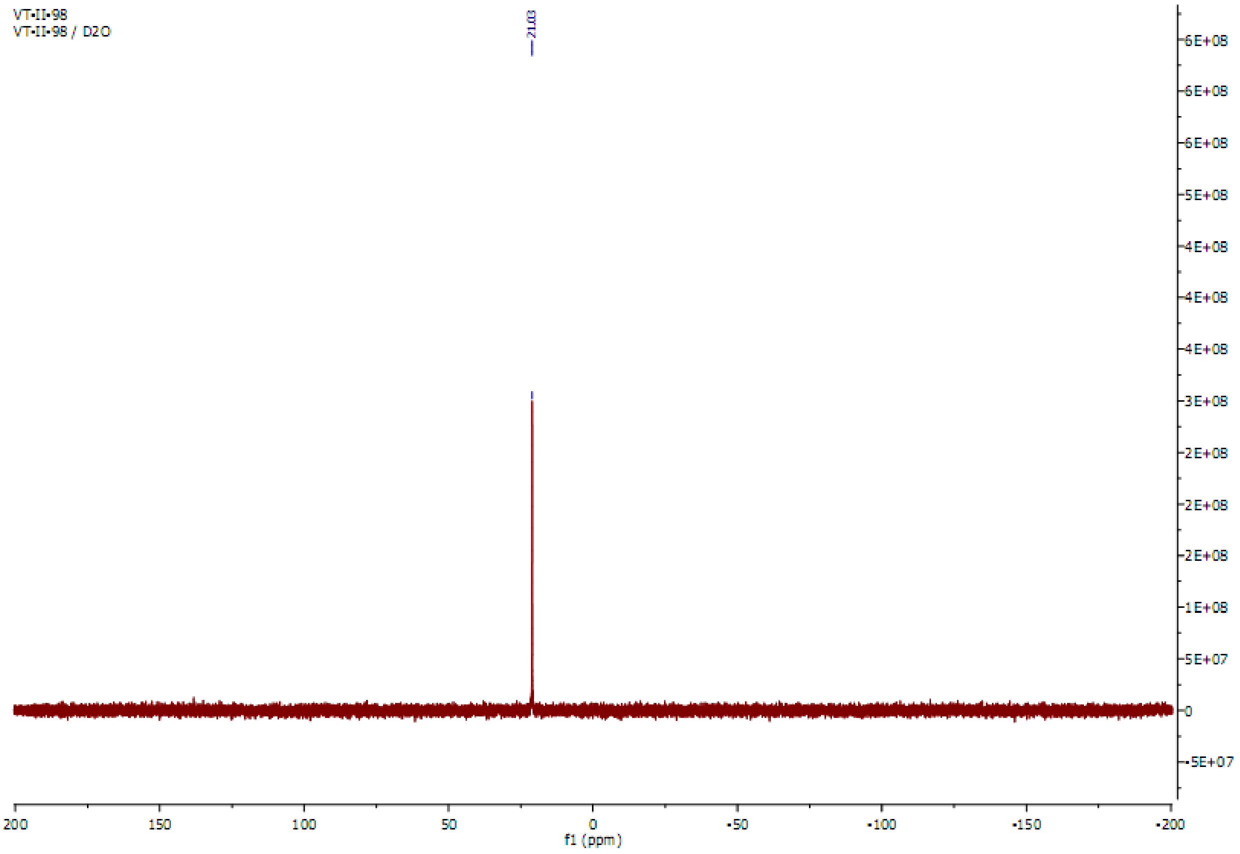

^{1}H NMR, ^{31}P NMR for compound **1q**

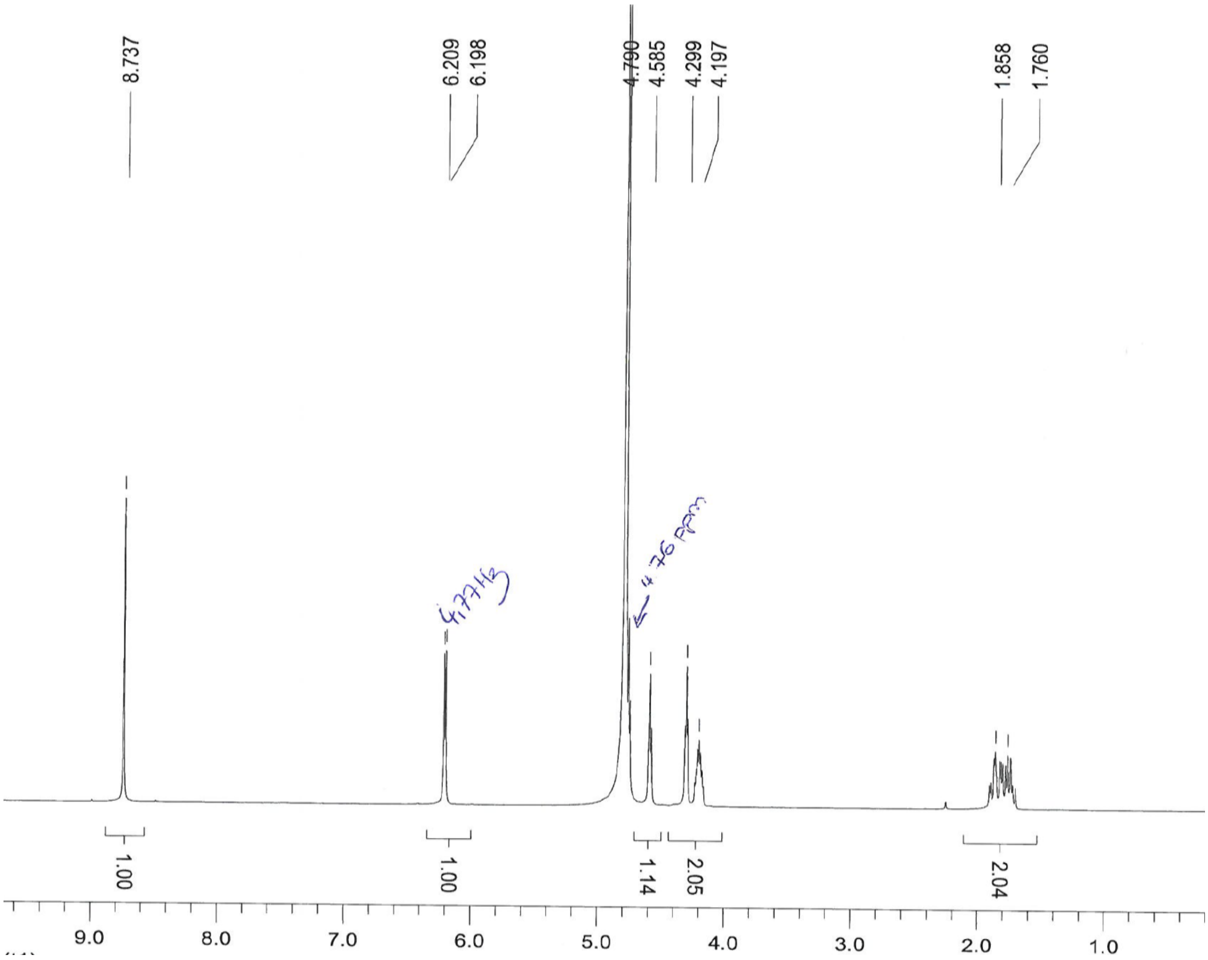

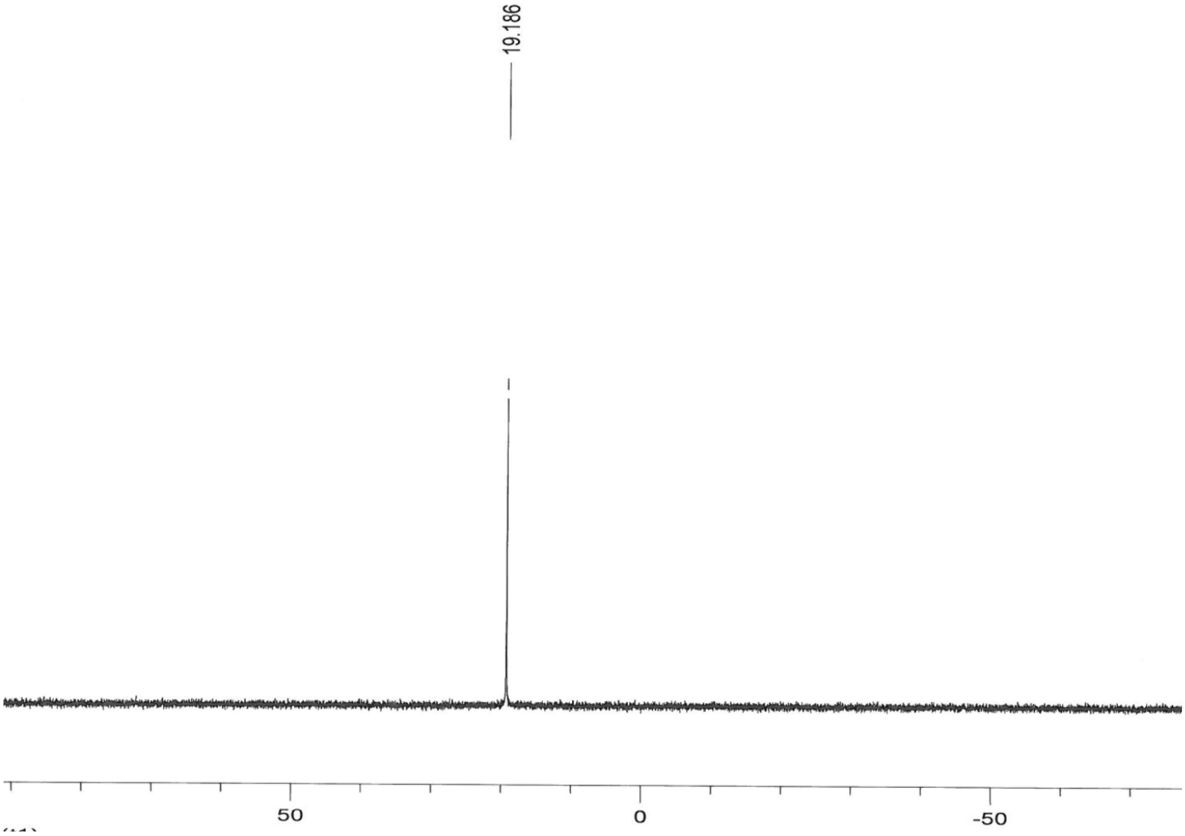

Supplement: File 2 — Copies of spectra for final compounds and NMR studies. [file Beilstein_J_Org_Chem-12-1476-s002.pdf]
